# Comparative effectiveness and acceptability of HIF prolyl-hydroxylase inhibitors *versus* for anemia patients with chronic kidney disease undergoing dialysis: a systematic review and network meta-analysis

**DOI:** 10.3389/fphar.2023.1050412

**Published:** 2023-07-13

**Authors:** Qiong Huang, Minling You, Weijuan Huang, Jian Chen, Qinming Zeng, Longfeng Jiang, Xiuben Du, Xusheng Liu, Ming Hong, Jing Wang

**Affiliations:** ^1^ Department of Nephropathy, Luohu District Traditional Chinese Medicine Hospital, Shenzhen, China; ^2^ Guangzhou University of Chinese Traditional Medicine, Guangzhou, China; ^3^ LuoHu Center for Chronic Disease Control, Shenzhen, China; ^4^ Institute of Advanced Diagnostic and Clinical Medicine, Zhongshan City People’s Hospital, Affiliated Zhongshan Hospital of Sun Yat-sen University, Zhongshan, China

**Keywords:** HIF-PHIs, ESAS, effectiveness, DD-CKD, anemia, acceptability

## Abstract

**Background**: The comparative benefits and acceptability of HIF-PHIs for treating anemia have not been well researched to date. We sought to compare the effectiveness of 6 HIF-PHIs and 3 ESAs for the treatment of renal anemia patients undergoing dialysis.

**Data sources:** Cochrane Central Register of Controlled Trials, PubMed, Embase, Cochrane Library, MEDLINE, Web of Science, and clinicaltrials.gov databases.

**Results:** Twenty-five RCTs (involving 17,204 participants) were included, all of which were designed to achieve target Hb levels by adjusting thee dose of HIF-PHIs. Regarding the efficacy in achieving target Hb levels, no significant differences were found between HIF-PHIs and ESAs in Hb response at the dose-adjusted designed RCTs selected for comparison. Intervention with roxadustat showed a significantly lower risk of RBC transfusion than rhEPO, with an OR and 95% CI of 0.76 (0.56–0.93). Roxadustat and vadadustat had higher risks of increasing the discontinuation rate than ESAs; the former had ORs and 95% CIs of 1.58 (95% CI: 1.21–2.06) for rhEPO, 1.66 (1.16–2.38) for DPO (darbepoetin alfa), and 1.76 (1.70–4.49) for MPG-EPO, and the latter had ORs and 95% CIs of 1.71 (1.09–2.67) for rhEPO, 1.79 (1.29–2.49) for DPO, and 2.97 (1.62–5.46) for MPG-EPO. No differences were observed in the AEs and SAEs among patients who received the studied drugs. Results of a meta-analysis of gastrointestinal disorders among AEs revealed that vadadustat was less effect on causing diarrea than DPO, with an OR of 0.97 (95% CI, 0.9–0.99). Included HIF-PHIs, were proven to be more effective than ESAs in reducing hepcidin levels and increasing TIBC and serum iron level with OR of −0.17 (95% CI, −0.21 to −0.12), OR of 0.79 (95% CI, 0.63–0.95), and OR of 0.39 (95% CI, 0.33–0.45), respectively.

**Conclusion:** HIF-PHIs and ESAs have their characteristics and advantages in treating anemia undergoing dialysis. With the selected dose-adjusted mode, some HIF-PHIs appeared to be a potential treatment for DD-CKD patients when ompared with rhEPO, due to its effectiveness in decreasing the risk of RBC transfusion rate or regulating iron or lipid metabolism while achieving target Hb levels.

**Systematic Review Registration:** https://www.crd.york.ac.uk/PROSPERO/display_record.php?RecordID=306511; Identifier: CRD42022306511

## 1 Introduction

Chronic kidney disease (CKD) is a growing worldwide problem. It affects the health of approximately 10% of adults, and in approximately 20 years, it will be the fifth leading cause of human death (Kalantar-Zadeh et al., 2021). As a common complication of CKD, anemia is associated with increased morbidity and mortality in patients (Coresh et al., 2007). As renal function decreases, the prevalence of anemia increases; this prevalence tends to be higher in CKD patients undergoing haemodialysis than in those who do not receive dialysis (Akizawa et al., 2018). Studies have highlighted the shortcomings of this common therapeutic approach to treating anemia in CKD patients, including insensitivity and inconvenience. However, ESA therapy targeting higher haemoglobin levels leads to increased risks for vascular and fatal events compared with therapy targeting lower levels (Palmer et al., 2010). A high dose of ESAs leads to an increased risk of vascular access thrombosis, cardiovascular and cerebrovascular events, and cancer-associated mortality (Szczech et al., 2008; Koulouridis et al., 2013). Therefore, it is meaningful to set the ‘Hb target’ to aim at ESA and to a more individualized approach, thus balancing the risk and benefits of ESA therapy (McMurray et al., 2012).

The mechanisms underlying how cells and tissues monitor and respond to oxygen levels remained unclear until the late 20th century. In 2019, three scientists (Willam Kaelin, Peter Ratcliffe, and Gregg Semenza) shared the Nobel Prize in Medicine or Physiology for discovering the hypoxia-inducible factor (HIF) oxygen-sensing pathway, which influences the erythropoietic response to hypoxia (Hurst, 2016; Fandrey et al., 2019). Cellular responses to hypoxia are regulated by the HIF-prolyl hydroxylase domain (PHD) pathway, which is related to the pathophysiology of multiple diseases, such as ischaemic diseases, anemia, polycythaemia, cancer, and pulmonary arterial hypertension (Semenza, 2011). HIF-PHIs are a new class of drugs that can activate HIF transcription factors and may have broad therapeutic potential in clinical practice (Schödel and Ratcliffe, 2019).

Because they are effective in activating erythropoietin and regulating iron metabolism, roxadustat (FG-4592), daprodustat (GSK-1278863), vadadustat (AKB-6548), enarodustat (JTZ-951), molidustat (BAY 85–3,934) and desidustat (ZYAN1) are approved for the treatment of anemia in CKD. Among these HIF-PHIs, the first four compounds are approved for marketing in Japan, and roxadustat is also approved for clinical usage in China, South Korea, the UK, Europe, and Chile. Desidustat received its first approval in India in March 2022, and enarodustat has progressed into clinical development in the United States and South Korea (Dhillon, 2019; Dhillon, 2020; Markham, 2020; Markham, 2021; Dhillon, 2022; Miao et al., 2022). Recently, FDA approval of Daprodustat for dialysis patients after al least 4 months of treatment in February 2023. Some meta-analyses have found that HIF stabilizers, such as roxadustat, vadadustat and daprodustat, can increase Hb levels and regulate iron metabolism in nondialysis-dependent (NDD) and dialysis-dependent (DD) patients (Fu et al., 2022; Huang et al., 2022; Wang et al., 2022). A more systemic network meta-analysis including all relevant drugs (roxadustat, daprodustat, vadadustat, enarodustat, molidustat, desidustat, ESAs) is necessary to analyse the comparative effects of HIF-PHIs and ESAs for treating renal anemia. One study reported that all HIF-PHIs have the same effect on clinical treatment as ESAs (EPO or DPO) in NDD-CKD patients (Zheng et al., 2020). However, NDD-CKD and DD-CKD should be evaluated separately due to the differences between the trajectories of Hb levels in each condition and in the haemodynamic and metabolic milieus and the different mechanisms of cardiac failure events (Parfrey, 2021). We conducted a systematic review and network meta-analysis (NMA) of RCTs to compare the efficacy in achieving target Hb levels and acceptability of different HIF-PHIs and ESAs for treating anemia in CKD patients undergoing dialysis.

## 2 Methods

### 2.1 Data sources and searches

An extensive literature search was performed in PubMed, Cochrane Library, Web of Science, Embase, clinicaltrials.gov databases, and MEDLINE up to 1 June 2022. In addition, the reference lists of all identified publications, drug manufacturers’ websites, and relevant meta-analyses were also mined for further relevant data (search terms and strategies are detailed in Supplementary Table S1). Our *a priori* inclusion criteria included 1) anaemic patients with DD-CKD treated with HIF-PHD inhibitors or ESAs (rhEPO, DPO, MPG-EPO), 2) intervention of rhEPO means epoetin alfa and beta or both of them, 3) reported efficacy outcomes and tabulated data on discontinuation outcomes, 4) control groups that were treated with ESAs, and 5) all interventions were designed to be dose-adjusted for the purpose of maintaining target Hb levels.

### 2.2 Study selection

Two authors independently screened citations against the following predefined selection criteria. Eligible papers were RCTs that compared the effects and safety of different anemia treatments in CKD patients undergoing dialysis. All superiority, phase II and III, nonblinded, single-blinded, noninferiority, and double-blinded trials were included. Interventions of interest included 6 HIF-PHIs (roxadustat, vadadustat, daprodustat, enarodustat, molidustat, and desidustat) and 3 ESAs (rhEPO, DPO and MPG-EPO). The exclusion criteria were as follows: 1) participants in the study had primary anemia or anemia resulting from blood loss, cancer, or infectious diseases, 2) only one drug for treating CKD with anemia was studied (for example, comparisons of different doses or dosing frequency of the same drug), 3) participants were <18 years old, 4) outcome data could not be sourced from authors, 5) the control group was a placebo, or 6) ESAs were unknown or EPO and DPO combinations were used.

### 2.3 Data extraction and quality assessment

Three authors (Xiuben Du and Minlin You) independently extracted data from the original trial reports using a standardized method and then verified the extraction. Disagreements were resolved *via* discussion among reviewers. We contacted the corresponding authors and sponsoring pharmaceutical companies of the included trials to request missing data. Data extraction was performed using a self-designed data extraction method (Longfeng Jiang). The main study characteristics included the year of publication, authors, clinical trial number, type of study, population characteristics, sample size, dosage of drugs, treatment duration, control treatment, race, gender, mean age, baseline Hb level, type of replacement therapy, iron supplement, definition of Hb response, and primary and secondary outcomes. We assessed sources of bias using the Cochrane Collaboration’s risk-of-bias tool, which addresses 9 domains (Higgins, 2008). Two authors (Ming Hong and Qinming Zeng) independently completed the assessments, and discrepancies were discussed with one author (Jing Wang) and resolved by consensus. Additionally, the GRADE guidelines were used to assess the quality of evidence contributing to each estimated network (Higgins et al., 2014).

### 2.4 Outcome definition

The primary outcome included Hb response associated with interventions. The drug acceptability profile was assessed according to the reported rate of discontinuation, AEs and SAEs.

The secondary outcomes included the main reason for discontinuation, such as AEs, death, refusal of treatment, content withdrawal and kidney transplantation. In addition, the rate of RBC transfusion, iron-related parameters included hepcidin levels, ferritin, transferrin saturation (TSAT), TIBC, serum iron levels, C-reactive protein (CRP) level, low-density lipoprotein (LDL) levels, high-density lipoprotein (HDL) levels, total cholesterol levels, MACE and all-cause mortality.

### 2.5 Cochrane risk-of-bias tool

The risk of bias was assessed per the Cochrane Handbook and PRISMA guidelines for Systematic Reviews of Interventions. Additionally, we analyzed the certainty of evidence contributing to network estimates of the main outcomes with the GRADE framework (Salanti et al., 2014; Brignardello-Petersen et al., 2020).

### 2.6 Statistical analyses

The results are presented in network plots, in which the node size is proportional to the patient number, while the line thickness between the nodes is proportional to the trial number. The differential contributions of direct comparisons to the network summary effect are presented in contribution plots (Nikolakopoulou et al., 2019). Using random-effects pairwise meta-analysis and network meta-analysis in a frequentist environment, the summary odds ratios (ORs) are also calculated for binary outcomes and standardized mean differences for continuous variables. All comparisons were two-tailed, and a *p*-value <0.05 denoted statistical significance. The synthesis of study effect sizes is conducted *via* a random-effects network meta-analysis model. The surface under the cumulative ranking (SUCRA) presents the probability (unit: %) of superior effectiveness for each intervention compared with a theoretical ideal intervention. Thus, interventions were ranked by the SUCRA index.

Heterogeneity was examined by generating forest plots, including summary effects with 95% CIs and 95% prediction intervals for all comparisons. Prediction intervals represent CIs of the approximate predictive distribution of the future trial, considering heterogeneity (Higgins et al., 2009). Via a loop-specific approach, the potential inconsistencies were evaluated between the direct and indirect evidence within the network (Veroniki et al., 2013), and local inconsistencies were also calculated using the node-splitting method (White, 2015). Further, the design-by-treatment model was used to evaluate global inconsistencies of the whole NMA.

**FIGURE 1:**
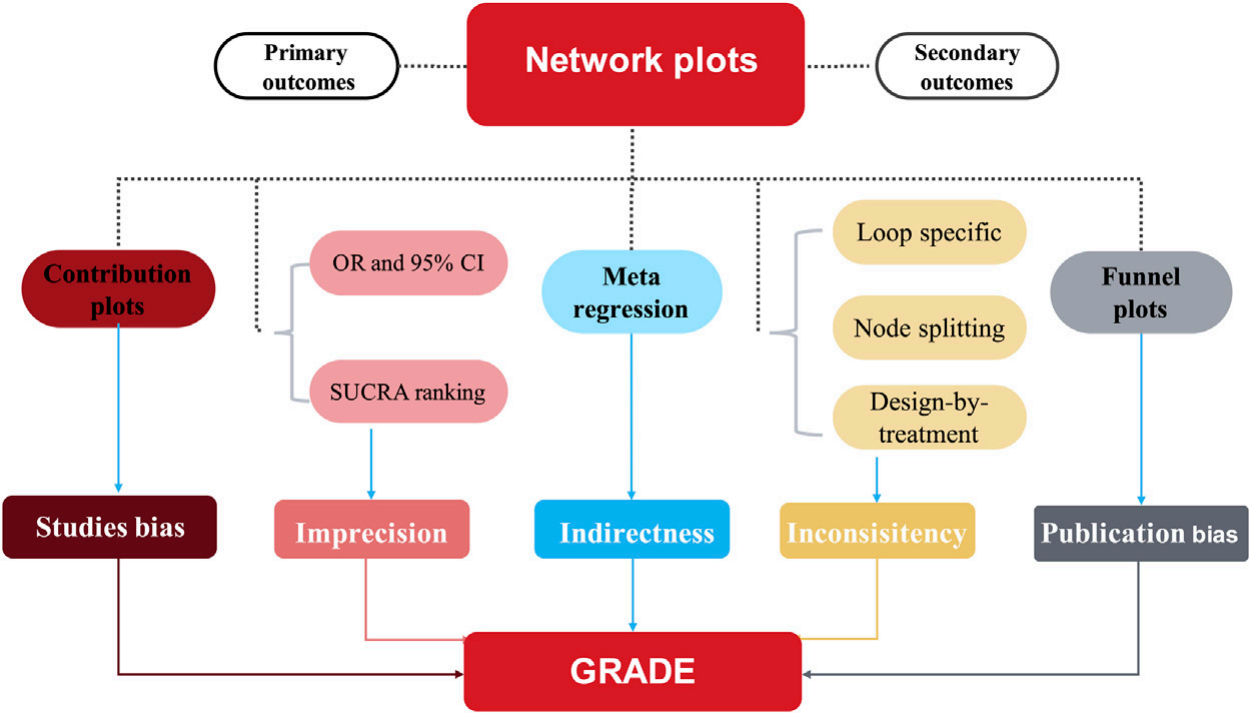
Flow chart of the Statistical analyses.

Finally, we used comparison-adjusted funnel plots and Egger’s regression to evaluate potentially small study effects and publication bias (Egger et al., 1997). We used meta-regression to assess the impact of study characteristics (effect modifiers) on the results. Subgroup analyses were conducted according to mean age (>60 or ≤60 years), the period of treatment (>24 or ≤24 weeks), Hb baseline level (>10.5 or ≤10.5 g/L), race (US or non US), sample size (>500 or ≤500 participants), follow-up (with or without), the target hemoglobin levels (10–12 or ≥11 g/dl) and dialysis method (hemodialysis or renal replacement therapy with both hemodialysis and peritoneal dialysis). All analyses were conducted using the network and network graph packages of Stata (version 15). The meta-analysis was prospectively registered with the PROSPERO international prospective register of systematic reviews (CRD42022306511) and reported by following the Preferred Reporting Items for Systematic Reviews and Meta-analyses extension for network meta-analyses (Hutton et al., 2015). (Figure 1)

### 2.7 Role of the funding source

The study founder had no role in the study design, data collection, analysis, interpretation, writing of the report, or the decision to submit.

## 3 Results

### 3.1 Study characteristics

**FIGURE 2:**
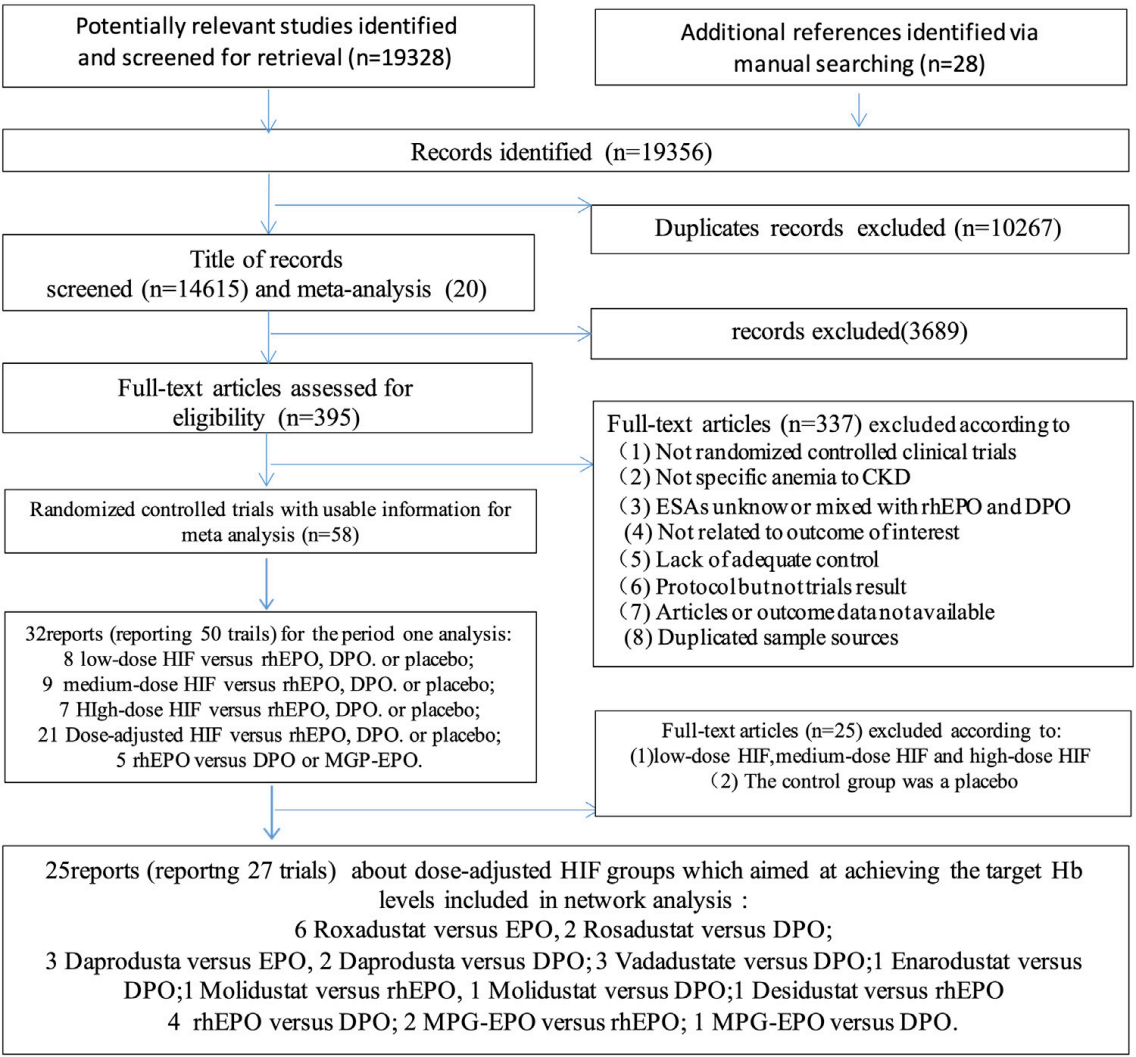
Flowchart of the current network meta-analysis.

Twenty-six eligible articles involving 17,204 patients published between 1990 and 2022 were included for pooled analyses (Nissenson et al., 2002; Vanrenterghem et al., 2002; Carrera et al., 2010; Bernieh et al., 2014; Brigandi et al., 2016; Holdstock et al., 2016; Provenzano et al., 2016; Akizawa et al., 2017; Chen et al., 2017; Akizawa et al., 2019; Bailey et al., 2019; Chen et al., 2019; Macdougall et al., 2019; Meadowcroft et al., 2019; Sinha et al., 2019; Akizawa et al., 2020a; Akizawa et al., 2020b; Akizawa et al., 2021a; Nangaku et al., 2021a; Akizawa et al., 2021b; Nangaku et al., 2021b; Charytan et al., 2021; Csiky et al., 2021; Eckardt et al., 2021; Provenzano et al., 2021; Singh et al., 2021). The literature search process is shown in Figure 2. These trials studied 9 different anti-anemia agents, including 6 different HIF-PHIs and 3 commonly used ESAs. There were 27 experiments included in these 25 articles, which contained the following comparisons: HIF-PHIs vs EPO (n = 11), HIF-PHIs vs DPO (n = 9), MPG-EPO vs rhEPO (n = 2), MPG-EPO vs DPO (n = 1), and rhEPO vs DPO (n = 4). Notably, one of the studies reported on two experiments that used different controls: HIF-PHI vs EPO and HIF-PHI vs DPO. One other study reported on two experiments that included incident and prevalent DD-CKD controls. The mean age of the patients ranged from 46.9 to 81 years, and the time of intervention varied from 7 to 104 weeks. The baseline characteristics of the included RCTs are provided in the Supplementary Material (Supplementary Table S2).

### 3.2 Network meta-analysis results

**FIGURE 3:**
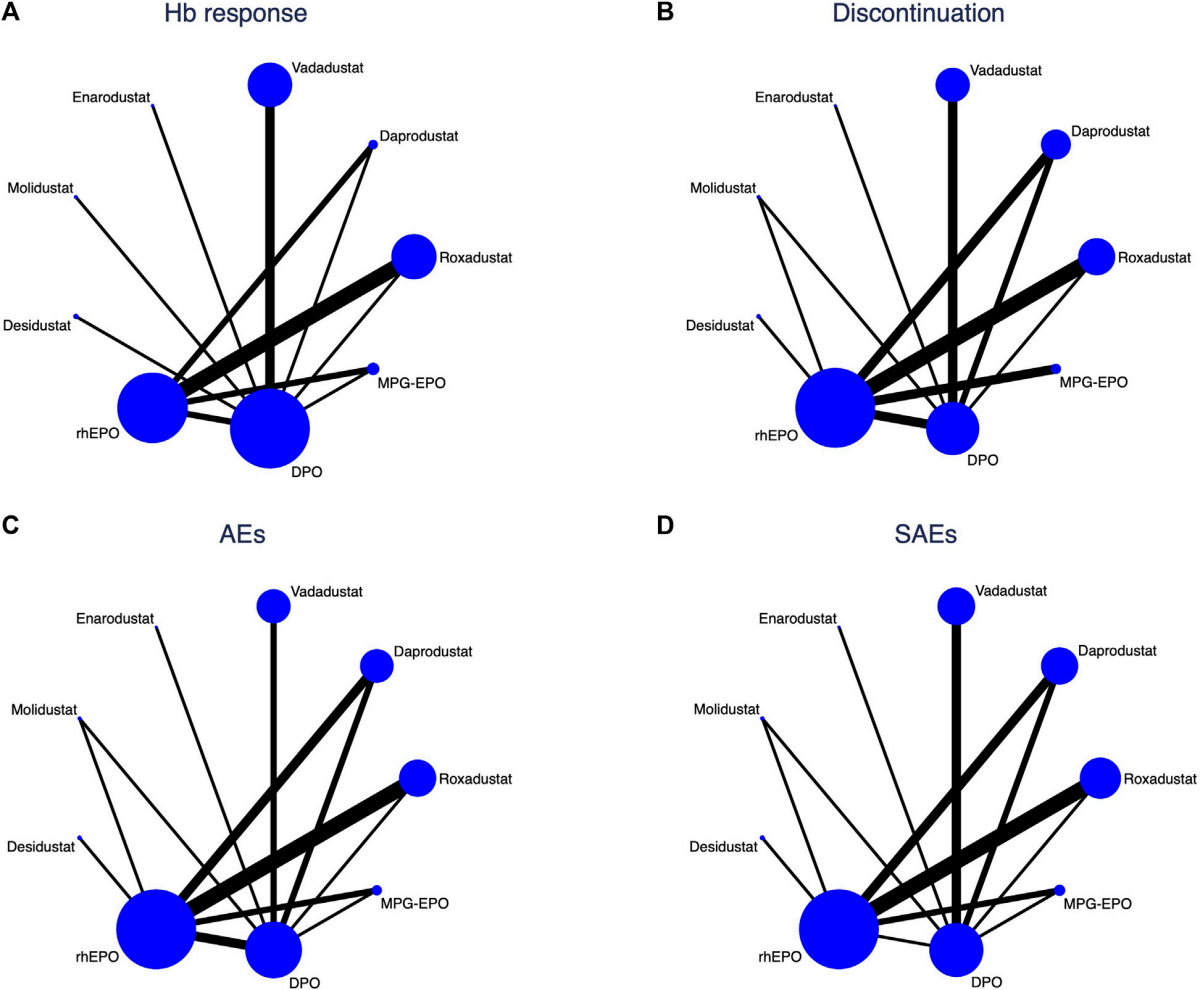
Network analysis of eligible comparisons for **(A)** Hb response **(B)** Discontinuation, **(C)** AEs, and **(D)** SAEs. The circle nodes present the patient numbers assigned to the trials, and the widths of lines denote the number of direct head-to-head comparisons available in contributing trials. When no head-to-head comparison was conducted, there is no line between the two nodes.

We assessed the efficacy and safety of 9 anti-anemia agents for use in anaemic CKD dialysis patients, including six different HIF-PHIs, rhEPO, DPO and MPG-EPO. Figure 3 shows the network of eligible comparisons for Hb response, discontinuation, AEs and SAEs.

### 3.3 Efficacy of Hb response and RBC transfusion

Regarding Hb response, our network meta-analysis included 20 RCTs involving the administration of 6 HIF-PHIs, rhEPO, DPO and MPG-EPO in 12,861 DD-CKD patients with anemia. No significant differences were found between HIF-PHIs and ESAs in reaching the Hb foreseen target. A meta-regression using restricted maximum likelihood estimators was exploited to assess the potential effects of duration, mean age, baseline Hb levels, race, sample size, follow-up, target haemoglobin levels and dialysis method on the change in Hb levels. The results of this meta-regression revealed that mean age had a significant effect on Hb response (Supplementary Table S4). No incoherence was observed between direct and indirect estimates.

Fifteen trials involving 10,314 participants were RCTs with dose-adjusted design that contributed to the analysis of the use of RBC transfusion as a rescue therapy (Supplementary Figure S1). Intervention with roxadustat showed a significantly lower risk of RBC transfusion than rhEPO, with an OR and 95% CI of 0.72 (0.56–0.93) (Table 1). No differences were found in the RBC transfusion rate between the included HIF-PHIs. Via the same moderating variables of this meta-regression, the results did not show a significant effect on RBC transfusion (Supplementary Table S4). No incoherence was observed between direct and indirect estimates.

**TABLE 1:** Network estimates of treatment comparisons for AEs and SAEs. The figure provides a summary of the estimates of odds ratios and 95% confidence intervals. For AEs the odds ratio is for the row treatment compared to the column treatment. For SAEs. the odds ratio is for the column treatment *versus* the row treatment.

Roxadustat	0.87 (0.61,1.24)	0.81 (0.55,1.19)	1.93 (0.45,8.40)	1.45 (0.79,2.68)	0.93 (0.44,2.00)	0.93 (0.79,1.11)	0.94 (0.68,1.32)	0.93 (0.66,1.31)
1.10 (0.72,1.67)	Daprodustat	0.93 (0.65,1.35)	2.23 (0.51,9.67)	1.67 (0.89,3.13)	1.08 (0.49,2.37)	1.08 (0.82,1.41)	1.09 (0.78,1.51)	1.07 (0.72,1.58)
1.69 (0.97,2.96)	1.54 (0.91,2.61)	Vadadustat	2.39 (0.57,10.11)	1.79 (0.98,3.28)	1.15 (0.51,2.63)	1.15 (0.81,1.64)	1.17 (0.97,1.41)	1.15 (0.79,1.67)
1.07 (0.40,2.88)	0.97 (0.36,2.63)	0.63 (0.23,1.71)	Enarodustat	0.75 (0.16,3.50)	0.48 (0.09,2.49)	0.48 (0.11,2.08)	0.49 (0.12,2.04)	0.48 (0.11,2.08)
1.32 (0.45,3.88)	1.20 (0.40,3.59)	0.78 (0.25,2.39)	1.23 (0.31,4.95)	Molidustat	0.64 (0.25,1.67)	0.64 (0.36,1.16)	0.65 (0.37,1.15)	0.64 (0.34,1.20)
1.02 (0.58,1.80)	0.93 (0.50,1.74)	0.60 (0.29,1.27)	0.96 (0.32,2.88)	0.78 (0.24,2.53)	Desidustat	1.00 (0.48,2.10)	1.01 (0.45,2.25)	0.99 (0.44,2.22)
1.09 (0.86,1.37)	0.99 (0.69,1.41)	0.64 (0.38,1.09)	1.02 (0.38,2.69)	0.83 (0.29,2.39)	1.06 (0.64,1.78)	rhEPO	1.01 (0.75,1.36)	0.99 (0.73,1.35)
1.44 (0.98,2.10)	1.31 (0.88,1.94)	0.85 (0.57,1.25)	1.34 (0.54,3.35)	1.09 (0.38,3.11)	1.41 (0.76,2.60)	1.32 (0.94,1.85)	DPO	0.98 (0.71,1.35)
1.52 (0.91,2.53)	1.38 (0.80,2.39)	0.90 (0.48,1.67)	1.42 (0.51,3.99)	1.15 (0.37,3.58)	1.49 (0.74,2.98)	1.40 (0.88,2.23)	1.06 (0.65,1.71)	MPG-EPO

### 3.4 Acceptability profile of discontinuation, AEs and SAEs

Twenty-four trials (15,794 participants) contributed to the analyses of discontinuation. Roxadustat and vadadustat had higher risks of increasing the discontinuation rate than ESAs; the former had ORs and 95% CIs of 1.58 (95% CI: 1.21–2.06) for rhEPO, 1.66 (1.16–2.38) for DPO, and 1.76 (1.70–4.49) for MPG-EPO, and the latter had ORs and 95% CIs of 1.71 (1.09–2.67) for rhEPO, 1.79 (1.29–2.49) for DPO, and 2.97 (1.62–5.46) for MPG-EPO. Except for enarodustat and desidustat, other HIF-PHIs and rhEPO led to a significantly higher risk of discontinuation than MPG-EPO, with ORs and 95% CIs of 1.76 (1.70–4.49) for roxadustat, 2.13 (1.27–3.58) for daprodustat, 2.97 (1.62–5.46) for vadadustat, 2.92 (1.11–7.68) for molidustat, and 1.74 and (1.15–2.65) for rhEPO (Table 1).

There were five main reasons for discontinuation: adverse events (AEs), death, subjects’ refusal of treatment, conforming to drug withdrawal contents, and kidney transplantation. Twenty trials (14,998 participants) were involved in the analysis of AEs, 13 trials (9,939) involved death, 14 trials (11,897) involved subjects refusing treatment, 19 trials (14.171) involved content withdrawal, and 11 trials (7,972) involved kidney transplantation. Roxadustat was associated with a higher risk of AEs that led to discontinuation than ESAs (rhEPO and DPO), with ORs and 95% CIs of 1.61 (95% CI: 1.19–2.17) for EPO and 2.06 (95% CI: 1.20–3.54) for DPO. Vadadustat had a higher risk of increasing AEs, subjects’ refusal to treatment, conforming to drug withdrawal contents, and rate than DPO, with ORs and 95% CIs of 2.04 (95% CI: 1.34–3.11), 2.22 (1.75–2.80), and 2.97 (1.50–5.88), respectively. No differences were found between the included interventions in the reasons for discontinuation, such as death and kidney transfusion (Supplementary Tables S8-S10). Via the moderating variables of this meta-regression, the results did not show a significant effect on the indicators of acceptability (Supplementary Table S4). No incoherence was observed between direct and indirect estimates (Supplementary Table S3).

Twenty-five trials (16,166 participants) and Twenty-four trials (15,144 participants) contributed to the analyses of AEs and SAEs, respectively. No differences were found in safety between HIF-PHIs, EPO, and DPO as determined by the AEs and SAEs (Table 1).The results did not reveal a significant effect on AEs and SAEs when the moderating variables duration, mean age, baseline Hb levels, race, sample size, follow-up, target haemoglobin levels and dialysis method were used (Supplementary Table S4). No incoherence was observed between direct and indirect estimates. (Supplementary Table S3). Meta-analysis results of HIF-PHIs *versus* ESAs for gastrointestinal disorders and some other common AEs revealed that vadadustat was superior to DPO in reducing the risk of diarrhea, with an OR of 0.97 (95% CI, 0.95–0.99); I2 = 0%. There was no difference between HIF-PHIs and rhEPO or DPO in increasing the risk of nausea, vomiting, hyperkalemia, and arteriovenous fistula thrombosis (Supplementary Figure S10).

### 3.5 Ranking of primary outcomes

**FIGURE 4:**
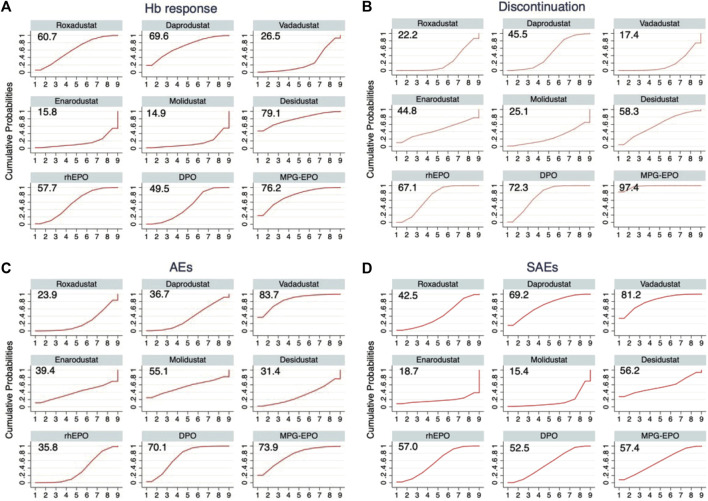
Drug ranking curves for **(A)** Hb response **(B)** Discontinuation, **(C)** AEs, and **(D)** SAEs. The cumulative probabilities of the intervention ranking are denoted by the curves for each outcome from the best to the worst, and the peak indicates the ranking with the highest probability for the corresponding intervention. The surface under the cumulative ranking (SUCRA) value is shown for each treatment and each outcome.

In the context of a dose-adjusted design randomized controlled trial selected for comparation, we used the previously calculated p-scores to rank the efficacy and acceptability of the drugs included in our study. A higher p-score indicated better efficacy or acceptability (Figure 4). Among the included drugs, desidustat ranks highest in the efficiency, with p-scores of 0.791 for Hb response at the doses selected for comparison. Since the top three at low discontinuation rates are all ESAs, they were associated with a higher acceptability when compared with HIF-PHIs (p-score = 0.974 for MPG-EPO, p-score = 0.723 for DPO, and p-score = 0.671 for rhEPO). Vadadustat was associated with the highest safety (p-score = 0.837 in AEs, and p-score = 0.812 in SAEs). Roxadustat ranked last in safety with p-score = 0.239 in AEs.

### 3.6 Secondary outcome: iron-related parameters and CRP

Overall, fourteen of the twenty trials (11,199 participants) reported data on hepcidin levels, 12 trials (11,143 participants) reported data on ferritin levels, 10 trials (7,233 participants) reported TIBC data, 12 trials (11,125 participants) reported TSAT data, and 9 trials (7,096 participants) reported serum iron levels were analysed. All the included HIF-PHIs, were proven to be more effective than ESAs in reducing hepcidin levels, and increasing TIBC and serum iron level with OR of −0.17 (95% CI, −0.21 to −0.12), OR of 0.79 (95% CI, 0.63–0.95), and OR of 0.39 (95% CI, 0.33–0.45), respectively. No differences in the effects on TSAT were found between the included HIF-PHIs and ESAs with OR of 0.04 (95% CI, −0.06–0.14) (Supplementary Table S7). Unlike rhEPO, included HIF-PHIs did not showed differ from DPO in reducing ferritin levels with OR of −0.17 (95% CI, −0.08 to −0.21) (Supplementary Table S7).

• It is well known that patients with the highest CRP levels require substantially higher ESA doses to achieve comparable Hb levels than those with lower CRP levels (Bradbury et al., 2009). While inflammation suppresses the haemoglobin response to ESAs, it does not appear to affect the dose of roxadustat (Chen et al., 2019). A connected network could not be generated to assess CRP levels under the premise that dose-adjusted design RCTs were selected for comparison. Nevertheless, a pairwise meta-analysis revealed that there was no evidence that roxadustat and rhEPO had different effects on decreasing CRP levels (OR, 0.87 [95% CI, 0.67–1.13]; I2 = 69.9%) (Supplementary Figure S8).

### 3.7 Secondary outcome: lipid metabolism, MACE and all-cause mortality

A connected network could not be generated to assess lipid metabolism. A pairwise meta-analysis revealed that roxadustat reduced LDL and total cholesterol levels more than rhEPO, with an OR of −0.55 (95% CI, −0.96 to −0.14); I2 = 93.9% and OR, −0.66 (95% CI, −0.86 to −0.14); I^2^ = 72.5%, respectively (Supplementary Figure S9). There were inadequate data to examine the coherence between estimates derived from direct and indirect evidence regarding LDL levels, HDL levels, and total cholesterol levels (Supplementary Figure S3).

Thirteen trials (13,095 participants) contributed to the analysis of MACEs and twenty-three trials (15,991 participants) contributed to the analysis of all-cause mortality (Supplementary Figure S1). There were no detectable differences in the odds of MACEs and all-cause mortality among the included interventions (Supplementary Figure S9 and Supplementary Figure S12). Via the moderating variables of this meta-regression, the results did not show a significant effect on MACEs and all-cause mortality (Supplementary Table S4).

### 3.8 Risk of bias and GRADE

We found that 25.92% (7/27 items), 59.25% (16/27 items), and 14.81% (4/27 items) of the included studies had an overall low, unclear, and high risk of bias, respectively. The blinding of outcome assessment and concealing procedure after randomization mainly contributed to the unclear risks of bias. The results of the risk of bias assessment of the trials are shown in Supplementary Table S5.

General symmetry was observed in the plots used to assess publication bias across the included studies. The results of Egger’s test indicated no significant asymmetry that might suggest publication bias among the articles included in this NMA (Supplementary Figure S7). In general, inconsistencies concerning either local inconsistency as assessed using the loop-specific approach and the node-splitting method or global inconsistency as determined using the design-by-treatment were not observed in this NMA (Supplementary Figure S3, Supplementary Figure S4, Supplementary Table S4). The GRADE evaluation is presented in Supplementary Table S6, which ranged from moderate to very low in the comparisons.

## 4 Discussion

### 4.1 Effectiveness and acceptability

Several meta-analyses have demonstrated that some HIF-PHIs are significantly superior to placebo in raising haemoglobin levels, and ESAs are superior to placebo in preventing blood transfusion (Palmer et al., 2014; Zheng et al., 2021a; Zheng et al., 2021b). However, most RCTs only showed that some HIF-PHIs were noninferior to some ESAs in the control group with respect to correction and maintenance of haemoglobin concentrations. To date, the specific difference between the various HIF-PHIs and ESAs has not been clarified. Our study is the first network meta-analysis that compared the effectiveness and acceptability of all available HIF-PHIs and ESAs for treating anemia in CKD patients undergoing dialysis. The comparison of results from the RCTs showed some interesting findings. First, all the included RCTs were designed to achieve the target level of Hb by adjusting the dose of the interventions. In this context, there are no significant differences between HIF-PHIs and ESAs in Hb response. Second, roxadustat achieves higher Hb levels than rhEPO and reduces the risk of red blood cell transfusion. An important finding pertains to RBC transfusions, which may result in nonimmunological complications and immunologically mediated transfusion reactions (Klein et al., 2007; McMurray et al., 2012) (Szczech et al., 2008; Palmer et al., 2010). According to the SUCRA analysis, roxadustat was at the top for reducing the risk of RBC transfusions when dose-adjusted design RCTS were selected for comparison. Apparently, treatment with it can reduce the risk of transfusion reactions and preserve the opportunity for future transplantation. Third, with regard to acceptability, although our results showed that in DD-CKD patients, the proportions of AEs and SAEs associated with the different HIF-PHIs were no differ from ESAs with the selected dose-adjusted mode, roxadustat and vadadustat showed a higher risk of increasing the discontinuation rate than ESAs, which ranged from rhEPO and DPO to MPG-EPO. On the other hand, these three ESAs ranked in the top three for reducing the risk of discontinuation, which was higher than all HIF-PHIs. Notably, the convenience of one monthly intravenous injection of MPG-EPO may account for its best performance in acceptability. There are five main reasons for discontinuation. Among them, roxadustat showed a significantly higher risk than rhEPO in terms of increased risk of AEs and subjects’ refusal of treatment. Additionally, there were significant differences between vadadustat and DPO in terms of increased risk of AES, subjects refusing treatment, and meeting required discontinuation, and vadadustat was higher than DPO. Last, with the selected dose-adjusted mode, although roxadustat were not significantly different in increasing the risk of AEs than others, it ranked worst in the AEs, which is one of the safety indicators, among the included interventions. To date, the incidence of most gastrointestinal disorders did not differ significantly between oral HIF-PHIs and SC/IV ESAs, however, surprisingly, oral vadadustat had less effect on causing diarrhea than DPO. Therefore, future research needs to explore the specific AEs and SAEs of HIF-PHIs when compared ESAs, extend this NMA to combine aggregate and individual patient data. Such an analysis will allow the prediction of personalized clinical outcomes, such as discontinuation due to some specific side effects, and the estimate of comparative efficacy at multiple time points. This can provide a more specific reference for clinical practice.

### 4.2 Effects on iron and lipid metabolism

HIF-PHIs regulate iron metabolism through five known mechanisms, some of which require further confirmation. Hepcidin is the main regulator of body iron homeostasis in patients with CKD. Elevated hepcidin levels may interfere with iron mobilization by impairing the mobilization of stored iron and iron absorption from the small intestine due to the degradation of ferroportin (FPN). First, HIF-PHIs can lead to the activation of erythropoiesis, and increased erythropoiesis reduces serum iron concentration because producing new RBCs is associated with a large demand for iron. Second, a mediator named erythroferrone (ERFE) derived from erythroblasts in the bone marrow and spleen can suppress hepcidin production. Thus, HIF-PHIs can downregulate hepcidin levels by reducing serum iron concentrations and promoting the production and secretion of ERFE (Kautz et al., 2014; Ueda and Takasawa, 2017; Hanudel et al., 2018; Hanudel et al., 2021). The excessive expression of hepcidin may result from inflammation and decreased renal clearance (Agarwal, 2021). However, HIF-PHIs have shown anti-inflammatory effects in several disease models, such as sepsis and acute ischaemic injury (Eltzschig et al., 2014; Taylor et al., 2016), which indicated that the HIF system is involved in the processes regulating inflammation and the immune response since hypoxia is a trigger for the inflammatory response and can possibly slow the recovery process (Vanderhaeghen et al., 2020). The benefit of HIF-PHIs in some studies shows that no dose increases are observed for the management of patients with high CRP levels randomized to HIF-PHI at variance with patients treated with ESA who required a higher dose to maintain Hb levels (Nangaku et al., 2021b; Charytan et al., 2021; Eckardt et al., 2021; Provenzano et al., 2021; Fishbane et al., 2022). This study has shown that most of the investigated HIF-PHIs could decrease hepcidin levels while also increasing TIBC levels better than rhEPO with the selected dose-adjusted mode. A few RCTs have reported changes in CRP levels, indicating that more evidence from RCTs in humans is needed to demonstrate whether this anti-inflammatory benefit of HIP-PHIs can directly lead to elevated haemoglobin levels and whether HIP-PHIs can reduce progression to ESRD or the slope of eGFR. HIF-PHIs such as vadadustat and molideustat can improve kidney function in mice with CKD. The improvement of CKD progression may be the fourth method through which HIF-PHIs reduce hepcidin levels (Wheeler and Clinkenbeard, 2019; Ikeda, 2021; Noonan et al., 2021). The final mechanism through which HIF-PHIs modulate iron metabolism involves the improvement of mineral and bone disorders. Under conditions of iron deficiency, the transcription of fibroblast growth factor 23 (FGF-23) is increased. High FGF-23 levels lead to enhanced urinary phosphate wasting and lower 1,25-dihydroxy vitamin D levels, resulting in decreased serum calcium levels, which causes increased parathyroid hormone levels. Parathyroid hormone has direct or indirect effects on erythropoietin release and shortens red blood cell survival (Meytes et al., 1981; Kimata et al., 2005). HIF-PHIs can suppress FGF-23 expression (Noonan et al., 2021). Five studies with roxadustat reported a lower need for iron (Chen et al., 2019; Charytan et al., 2021; Csiky et al., 2021; Provenzano et al., 2021; Fishbane et al., 2022), while other HIF-PHIs seem to have less impact on iron use and iron doses. However, whether this difference can be ascribed directly to a distinctive effect of various HIF-PHIs on iron requirements or indirectly to a different iron need induced by different Hb increases remains unclear. In conclusion, in addition to increasing endogenous erythropoietin production, HIF-PHIs may reduce the need for intravenous IV) iron supplementation by regulating iron metabolism (Haase, 2021). The benefits of regulating iron metabolism suggest that HIF-PHIs are a promising new class of orally administered drugs for treating anemia associated with CKD.

Although roxadustat and daprodustat are superior to epoetin alfa in lowering high LDL cholesterol and total cholesterol levels, which present a major risk factor for cardiovascular diseases among CKD patients (Holdstock et al., 2016; Charytan et al., 2021; Singh et al., 2021), no data from recent studies have shown that the cholesterol-lowering effect of roxadustat was associated with a lower incidence of MACEs, nor was rosuvastatin (Davidson, 2009). Several studies showed that statins are less efficacious in reducing cardiovascular disease risk in patients with CKD, particularly in patients on dialysis therapy (Massy and Zeeuw, 2013). Recent study shows the reason for the less effective of statins motioned above may be due, at least in part, to a higher intracellular cholesterol production by hyperphosphatemia, possibly *via* a lower membrane LDL receptor expression (Massy et al., 2022). Therefore, more research is needed on the role of HIF-PHIs in regulating lipid metabolism and its benefits in dialysis patients. With respect to all-cause mortality although there was no significant difference between interventions in mortality, roxadustat ranked last in reducing the risk of all-cause mortality. Thus, more large and high-quality studies are needed to confirm the safety profiles of roxadustat.

### 4.3 Potential use of different HIF-PHIs

There appeared to be between-drug differences in the effects on changes in Hb levels and discontinuation. Although roxadustat was associated with higher odds of discontinuation with the selected dose-adjusted mode, it was more effective than rhEPO in reducing the risk of RBC transfusion, which may be related to the higher haemoglobin levels obtained with roxadustat. This advantage will alleviate the imbalance between the supply and demand of its transfusion needs in some special periods and situation. Therefore, it is worthwhile to identify and reduce the side effects that lead to drug withdrawal, as it can improve tolerance while maintaining efficiency. Desidustat was developed in Asian populations. There was no significant difference between desidustat and ESAs in the incidence of discontinuation, and desidustat ranked the highest in Hb reaction and risk of drug discontinuation among HIF-PHIs, showing better efficacy and tolerance. In addition, vadadustat ranked highest in safety and was less effective at causing than DPO. Roxadustat, desidustat and vadadustat may be a potential option based on their effectiveness and acceptability, especially in cases where patients have a personal preference for oral therapy. Regarding the iron parameters, with the selected dose-adjusted mode, included HIF-PHIs were showed superior to ESAs in reducing hepcidin and ferritin levels and improving TIBC and serum iron levels. In addition, the synergistic effect of HIF-PHIs on erythropoietin release and iron/cholesterol metabolism may potentially reduce the need for iron supplementation and lipid-lowering drugs. Therefore, limiting the use of these prescriptions may decrease the occurrence of drug-related side effects, such as frequent gastrointestinal discomfort or even rhabdomyolysis.

### 4.4 Limitations

Based on direct and indirect evidence, our study provides a preliminary comprehensive ranking of the investigated drugs in terms of their effects on reaching Hb target levels and RBC transfusion, as well as their acceptability based on reported discontinuation; these findings provide a basis for future clinical research. However, our study has some limitations. First, since various HIF-PHI types are currently in phase II or III clinical trials, this study lacked direct head-to-head comparisons between different HIF-PHIs. More indirect comparisons between different agents resulted in reduced accuracy of the results. Second, the number of HIF-PHI RCTs differed for each drug. Thus, the results still need to be further verified by more studies (ClinicalTrials.gov numbers, NCT03400033, NCT03457701, NCT04134026, NCT04899661, *etc.*). Third, the evidence is limited on the effect of these treatments on changes in VEGF, CRP, lipid metabolism levels and changes in iron requirements in dialysis patients, and studying these parameters allows for a more comprehensive analysis of HIF-PHI characteristics. The efficacy and acceptability indicators of these drugs should be summarized and analysed in more studies.

## 5 Conclusion

This network meta-analysis compared all currently available HIF-PHIs for treating anemia in CKD patients undergoing dialysis. HIF-PHIs and ESAs have their own characteristics and advantages. ESAs have better performance than HIF-PHIs in terms of acceptability. With the selected dose-adjusted mode, some HIF-PHIs appeared to be a potential treatment for DD-CKD patients when compared with rhEPO, due to its effectiveness in decreasing the risk of RBC transfusion rate or regulating iron or lipid metabolism while achieving target Hb levels (Table 2).

**TABLE 2:** Network estimates of treatment comparisons for Hb response and discontinuation. The figure provides a summary of the estimates of odds ratios and 95% confidence intervals. For Hb response, the odds ratio is for the row treatment compared to the column treatment. For discontinuation, the odds ratio is for the column treatment *versus* the row treatment.

Roxadustat	0.77 (0.53,1.14)	1.08 (0.67,1.75)	0.81 (0.24,2.77)	1.06 (0.43,2.60)	0.67 (0.34,1.33)	0.63 (0.48,0.82)	0.60 (0.42,0.86)	0.36 (0.22,0.59)
0.89 (0.43,1.85)	Daprodustat	1.39 (0.86,2.25)	1.05 (0.31,3.58)	1.37 (0.56,3.35)	0.87 (0.43,1.76)	0.82 (0.60,1.12)	0.78 (0.55,1.10)	0.47 (0.28,0.79)
1.54 (0.72,3.29)	1.72 (0.73,4.09)	Vadadustat	0.75 (0.22,2.55)	0.98 (0.40,2.39)	0.62 (0.29,1.35)	0.59 (0.37,0.92)	0.56 (0.40,0.77)	0.34 (0.18,0.62)
2.37 (0.64,8.78)	2.66 (0.67,10.48)	1.54 (0.42,5.60)	Enarodustat	1.31 (0.31,5.49)	0.83 (0.21,3.25)	0.78 (0.23,2.62)	0.74 (0.23,2.40)	0.45 (0.12,1.61)
2.39 (0.68,8.43)	2.68 (0.71,10.08)	1.56 (0.45,5.37)	1.01 (0.20,5.17)	Molidustat	0.63 (0.22,1.87)	0.60 (0.25,1.43)	0.57 (0.25,1.30)	0.34 (0.13,0.90)
0.73 (0.25,2.10)	0.81 (0.26,2.54)	0.47 (0.17,1.33)	0.31 (0.07,1.35)	0.30 (0.07,1.28)	Desidustat	0.94 (0.50,1.77)	0.89 (0.45,1.80)	0.54 (0.25,1.15)
1.02 (0.69,1.53)	1.15 (0.61,2.14)	0.67 (0.33,1.34)	0.43 (0.12,1.54)	0.43 (0.13,1.45)	1.41 (0.51,3.90)	rhEPO	0.95 (0.71,1.28)	0.57 (0.38,0.87)
1.13 (0.64,1.97)	1.26 (0.63,2.53)	0.73 (0.44,1.22)	0.47 (0.15,1.55)	0.47 (0.15,1.45)	1.55 (0.63,3.82)	1.10 (0.69,1.76)	DPO	0.60 (0.36,1.00)
0.83 (0.41,1.66)	0.93 (0.41,2.12)	0.54 (0.24,1.21)	0.35 (0.09,1.33)	0.35 (0.10,1.26)	1.14 (0.38,3.43)	0.81 (0.45,1.47)	0.74 (0.39,1.38)	MPG-EPO

## Data Availability

The original contributions presented in the study are included in the article/Supplementary Material, further inquiries can be directed to the corresponding author.
