# Supplementary material for: Comparative effectiveness and acceptability of HIF prolyl-hydroxylase inhibitors versus for anemia patients with chronic kidney disease undergoing dialysis: a systematic review and network meta-analysis
Source: Front Pharmacol. 2023 Jul 13;14:1050412. doi: 10.3389/fphar.2023.1050412 (PMC10374033; doi:10.3389/fphar.2023.1050412)
Supplement: Supplementary file 1 [file DataSheet1.docx]

**Comparative Effectiveness and Safety of HIF Prolyl-Hydroxylase Inhibitors** **for Anemia Patients with Chronic Kidney Disease Undergoing Dialysis: A Systematic Review and Network Meta-analysis: Supplement Materials**

Qiong Huang M.D. ^1,3*^, Minling You M.D. ^1^, Weijuan Huang M.D. ^1^,

Jian Chen M.D. ^1^, Qinming Zeng M.D. ^1^, Longfeng Jiang M.D. ^1^, Xiuben Du M.D. ^2^, Xusheng Liu Ph.D^3^, Ming Hong Ph.D.^4*^, Jing Wang Ph.D^1*^

1. Department of nephropathy, Luohu district traditional Chinese medicine hospital, No. 16 Xian Tong Road, Shenzhen 518000, Guangdong province, China.

2. LuoHu Center for Chronic Disease Control, No. 11 Jinhu Road, Luohu District, Shenzhen

3. Guangzhou University of Chinese Traditional Medicine, No. 111 Dade Road, Guangzhou 510405, China.

4. Institute of Advanced Diagnostic and Clinical Medicine, Zhongshan City People’s Hospital, Affiliated Zhongshan Hospital of Sun Yat-sen University, No. 2 Sun Wen dong Road, Zhongshan 528400, Guangdong, China.

*Corresponding author:

Qiong Huang. Tel.: +86-(760)-82311523, Email address: 53394728@qq.com

Jing Wang, Tel.: +86-(760)-82311212, Email address: [1034952286@qq.com](mailto:1034952286@qq.com)

Ming Hong, Tel.: +86-(760)-88823566, Email address: 3227374597@126.com

**Figure S1: Network plots for secondary outcomes**


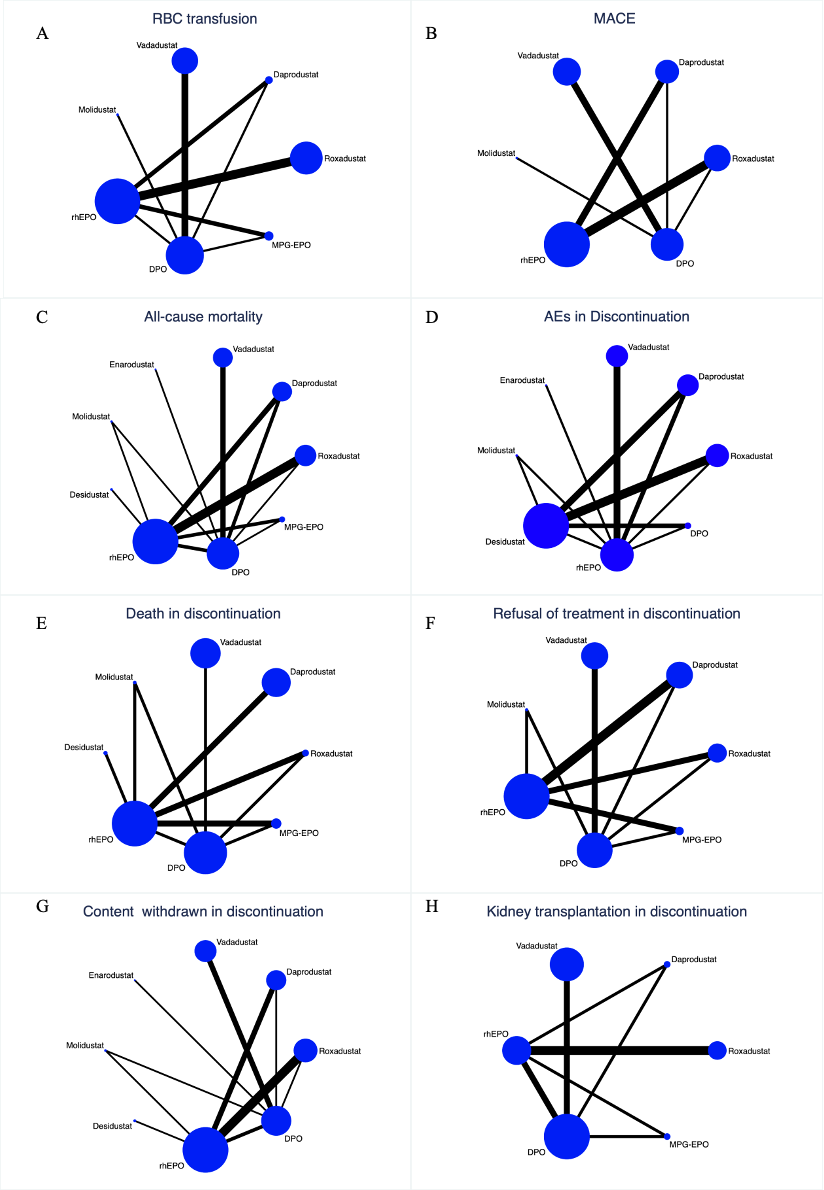


**Figure S2: Summary risk of bias assessment weighted by contribution of direct and indirect evidence to each pairwise comparison**


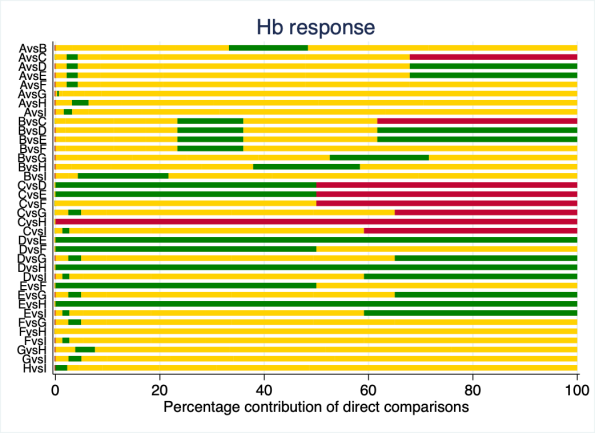

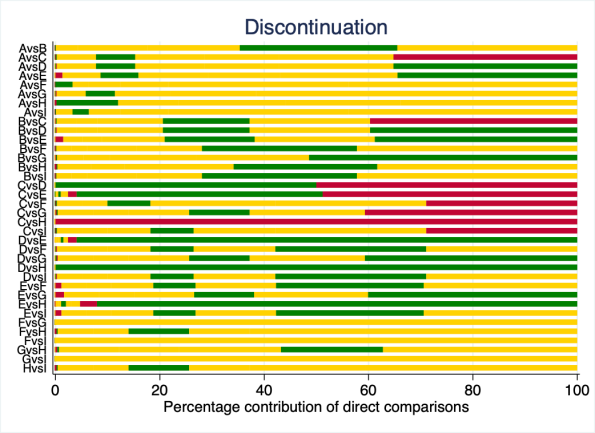

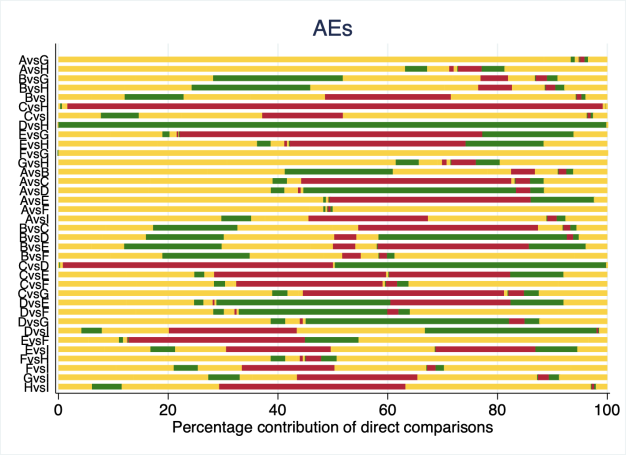

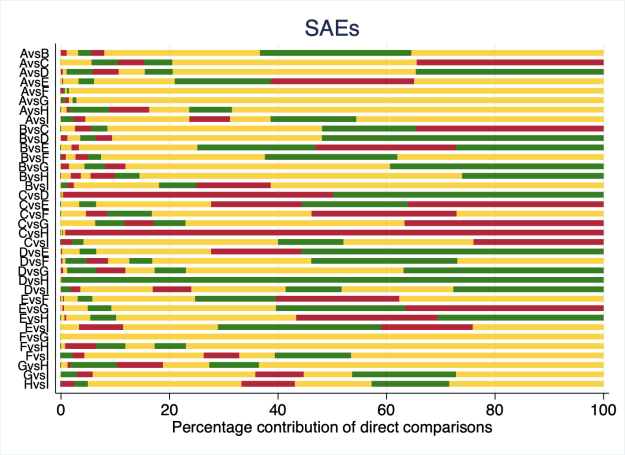

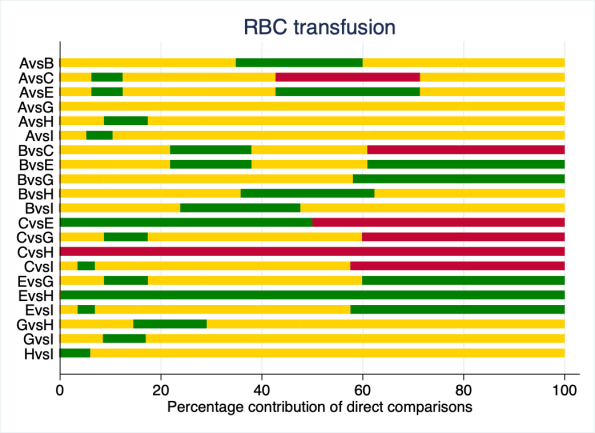

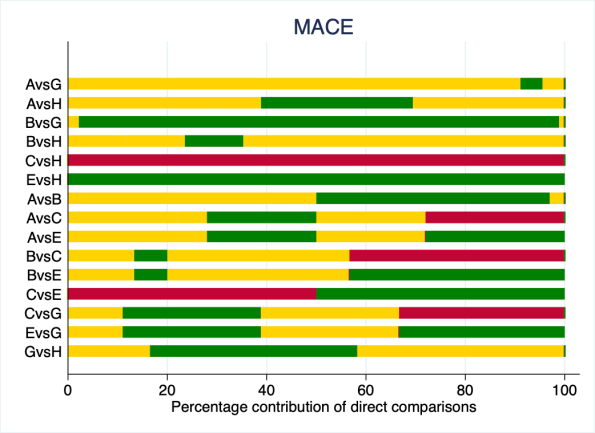

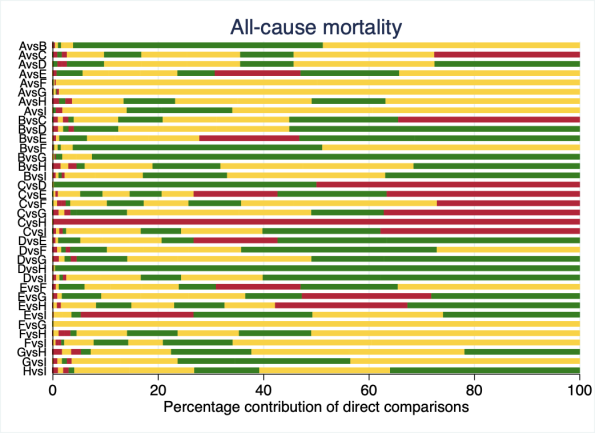

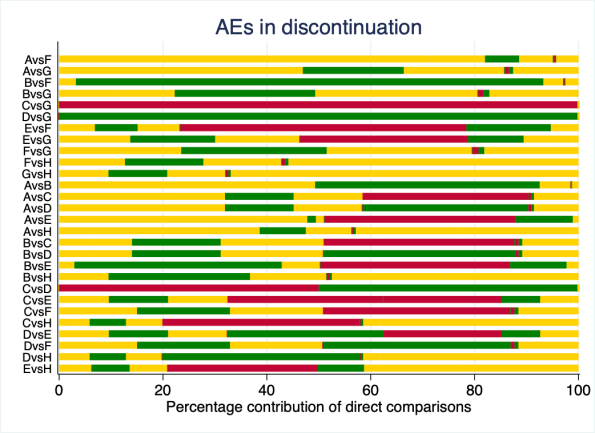


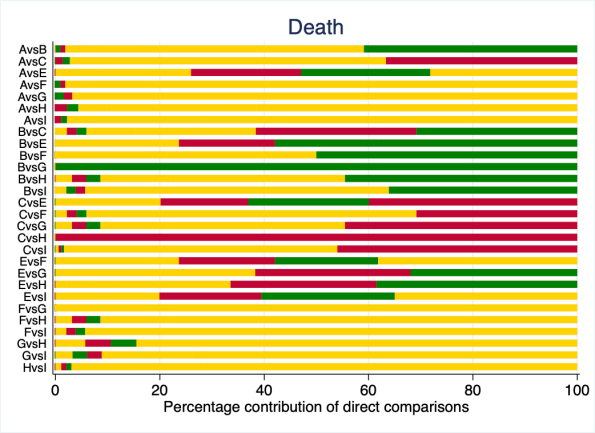

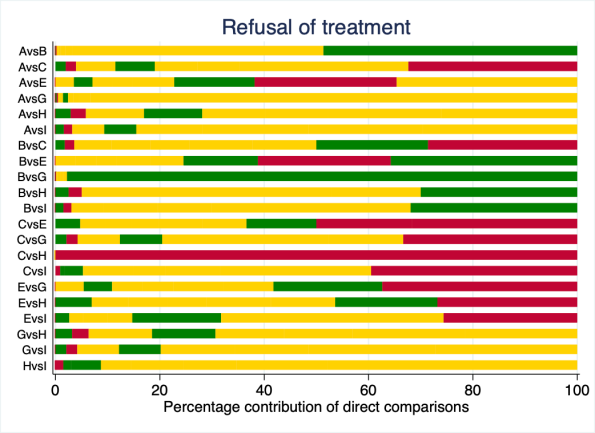


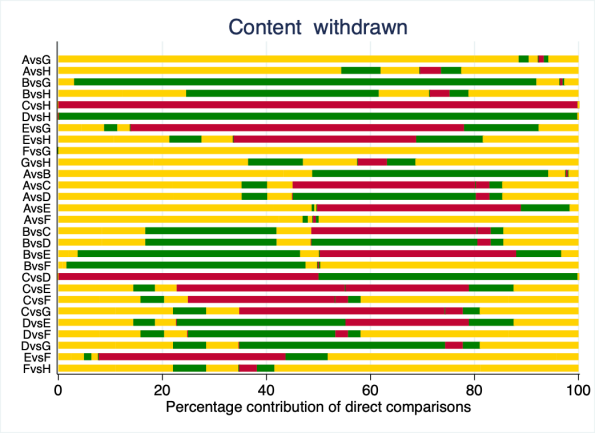

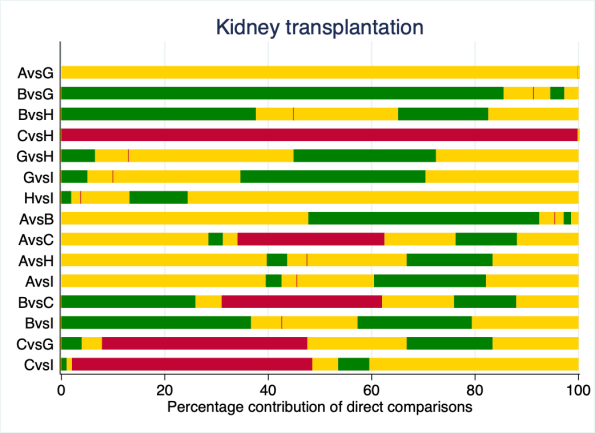


**High risk**

**Low risk**

**Moderate risk**

Treatment comparisons are restricted to the HIF-PHIs and ESAs compared to each other or PBO. First, the risk of bias in all trials within a treatment comparison were adjudicated using the Cochrane risk of bias tool. The overall contribution of each study to the overall network estimate was then calculated using a contribution matrix. The relative contribution of estimates based on low moderate and high risk of bias was determined based on the risk individual studies and their contribution to the network estimate.

Abbreviations: A=Roxadustat; B=Daprodustatt; C=Vadadustate; D=Enarodustat; E=Molidustat; F=Desidustat; G=rhrhEPO; H=DPO;I=MPG-EPO.

**Figure S3: Inconsistency of different intervention: loop inconsistency**


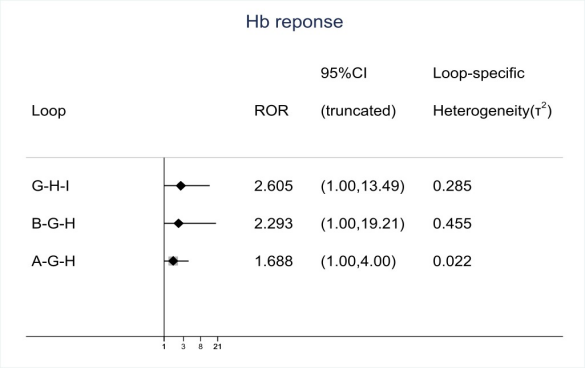

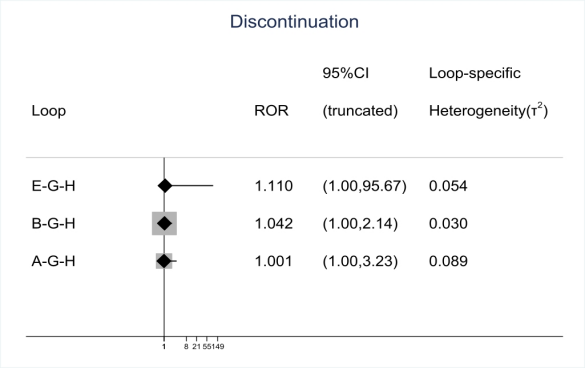

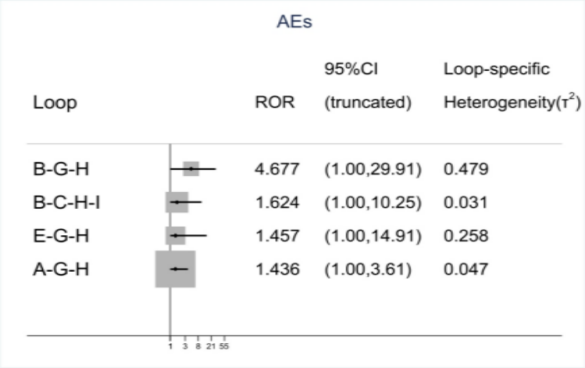

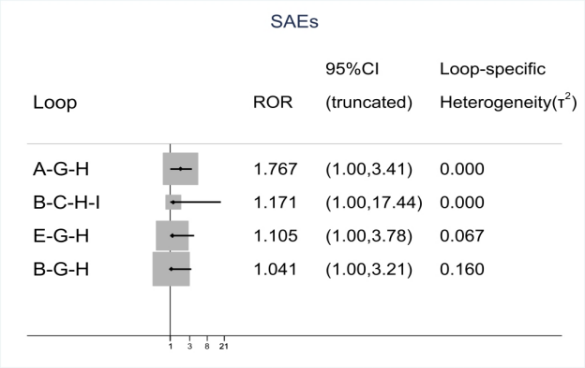

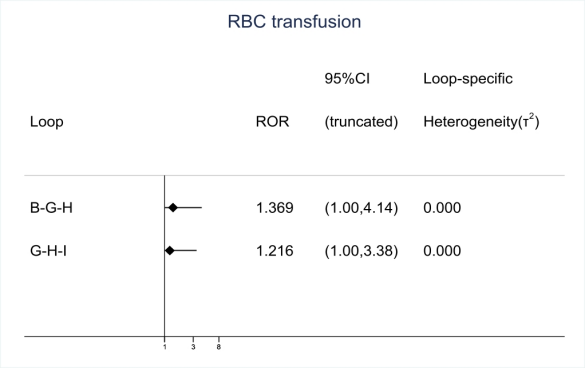

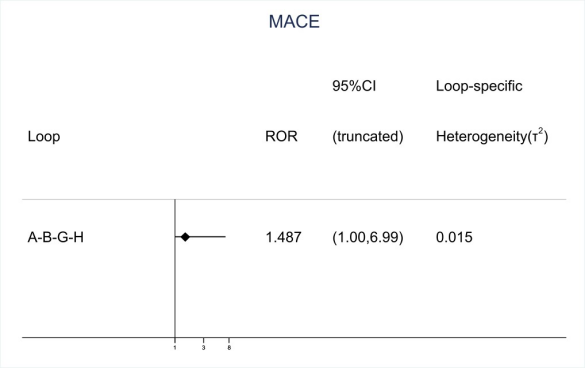

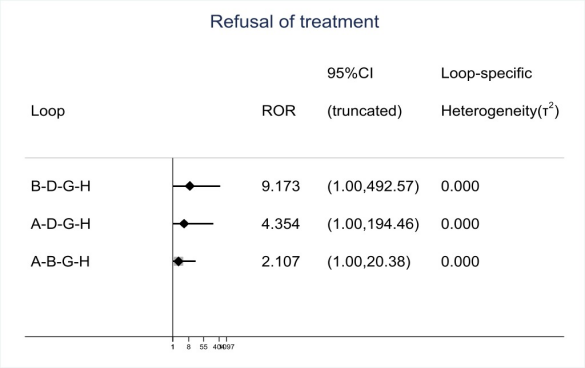

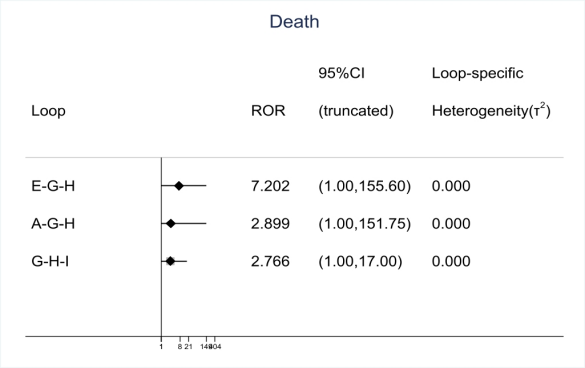


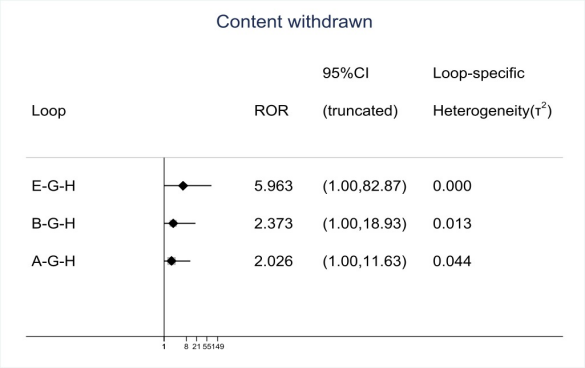

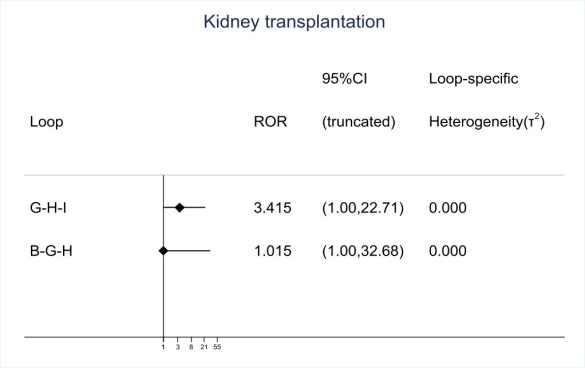


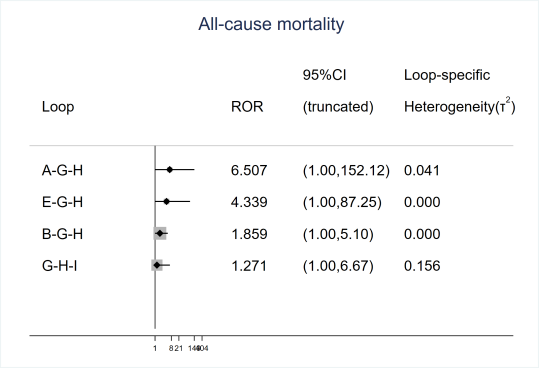


The ratio of odds ratios for direct and indirect estimates with their 95% confidence intervals.

Abbreviations: A=Roxadustat; B=Daprodustatt; C=Vadadustate; D=Enarodustat; E=Molidustat; F=Desidustat; G=rhEPO; H=DPO;I=MPG-EPO. A confidence interval including 1 indicates there is no statistically detectable inconsistency between direct and indirect treatment estimates. The network for MACE and RBC translation did not have any loops of evidence.

**Figure S4: Inconsistency of different intervention: design-by-treatment**


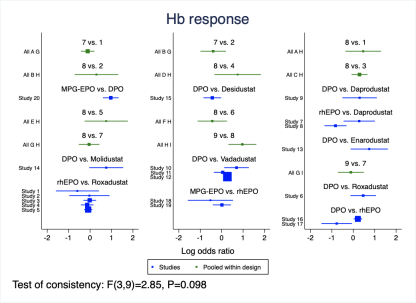

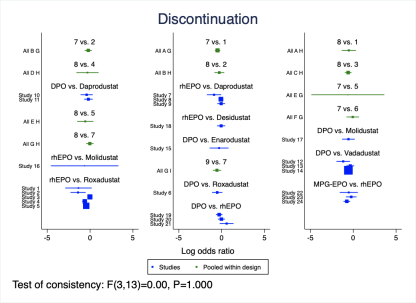

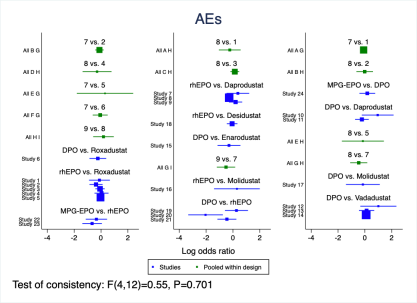

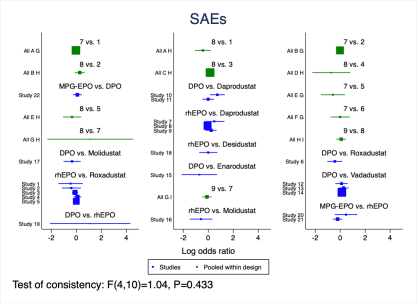

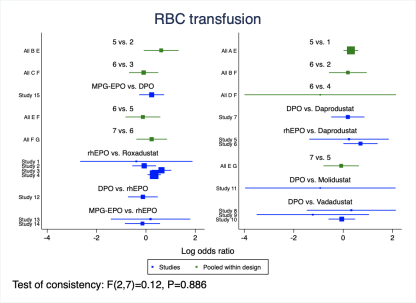

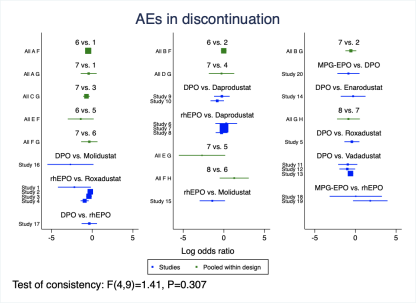


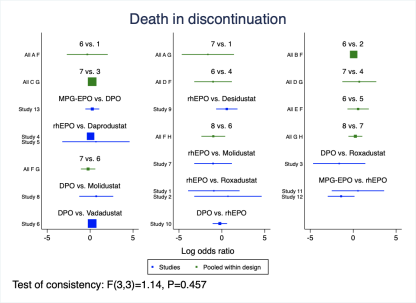

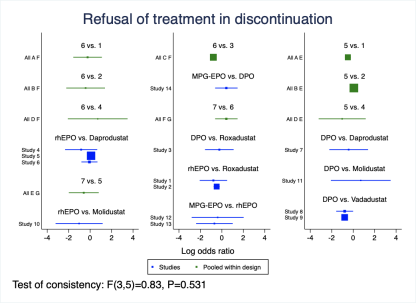

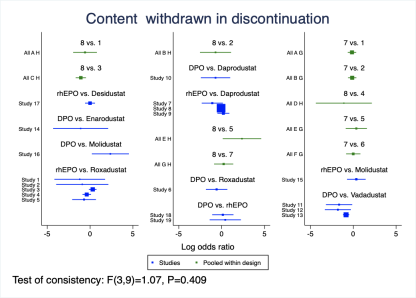

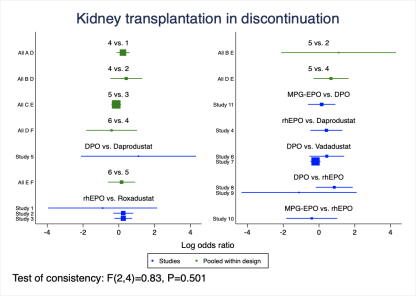

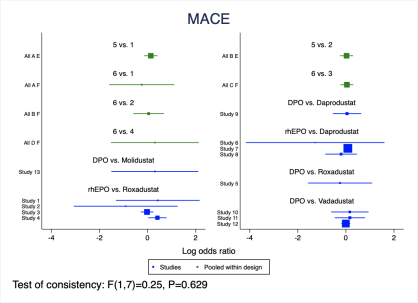

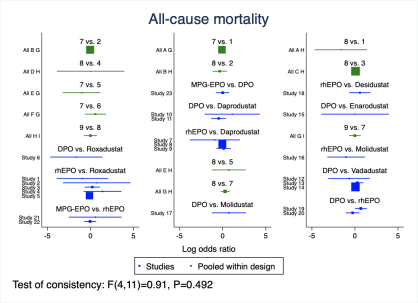


**Table S5: Treatment rankings using Surface Under the Cumulative Ranking (SUCRA) Curve**


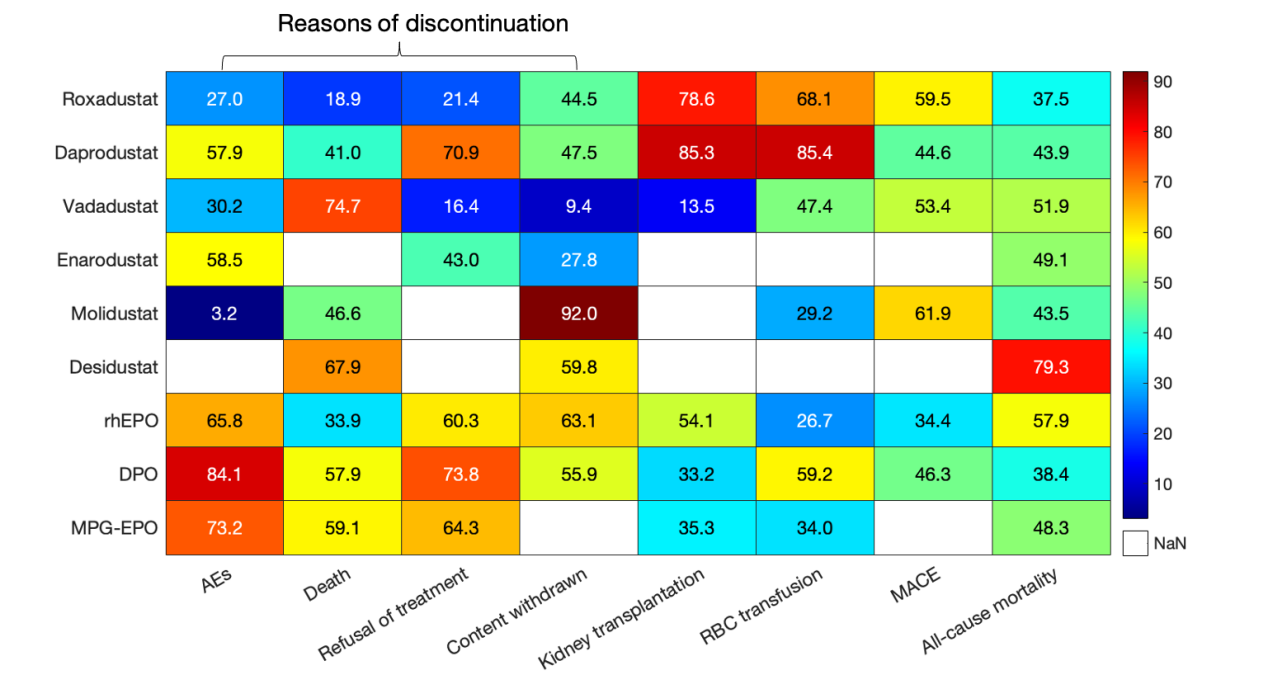


Abbreviations: CI = confidence interval; PrI = prediction interval. A 95% prediction interval is an estimate of the interval in which a future observation will fall, with a 95% probability. This indicates the interval in which a treatment estimate for a network meta-analysis including a future as yet unavailable trial will likely fall with 95% probability. If an estimate falls to the right side of 1, then this means that the treatment effect is favorable for the intervention in the left column versus the comparator

**Figure S6: Comparison-adjusted funnel plots**


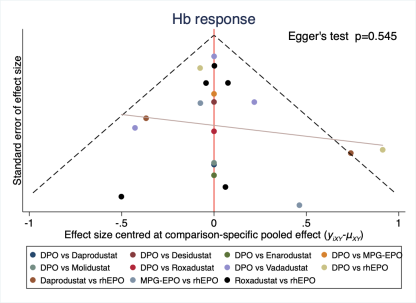

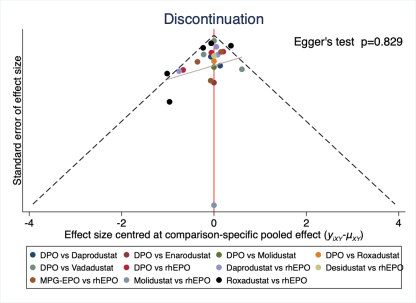

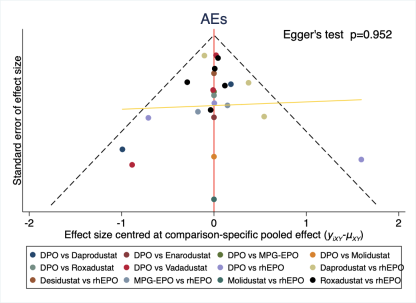

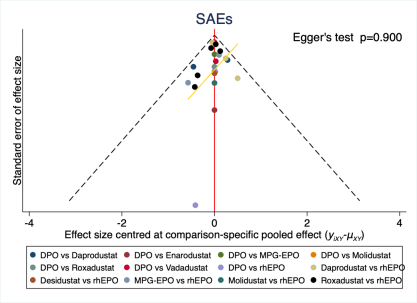

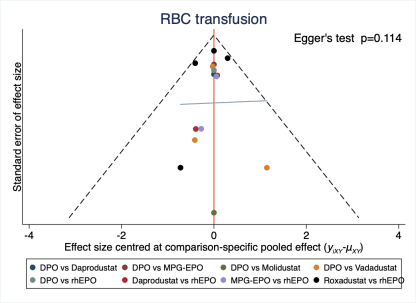

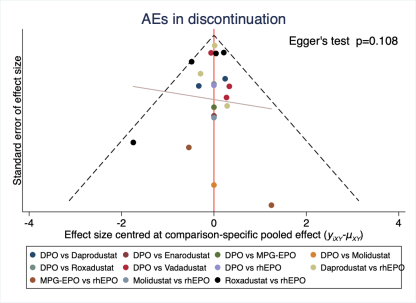

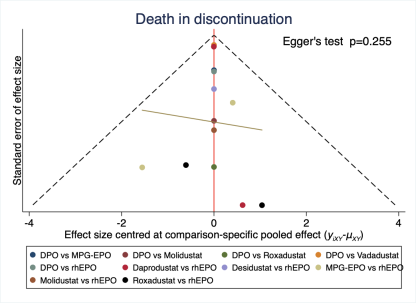

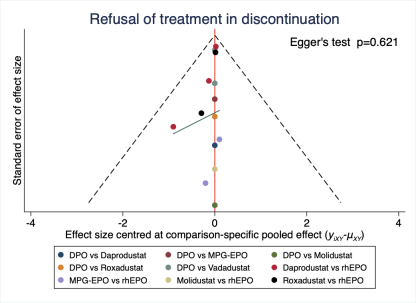

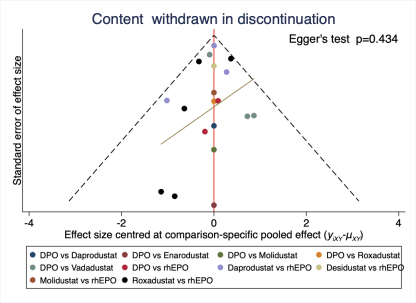

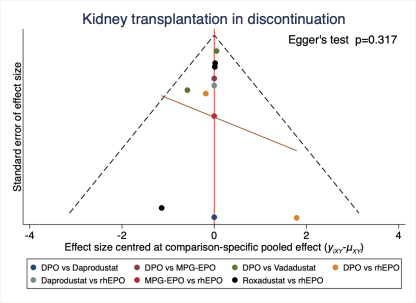

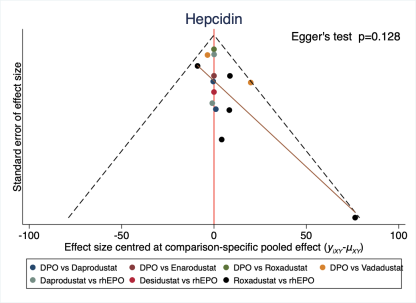

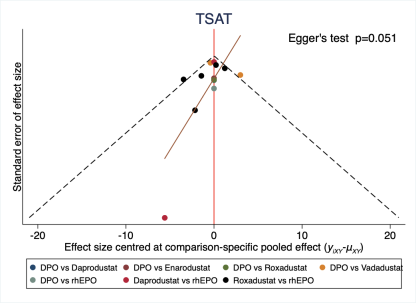

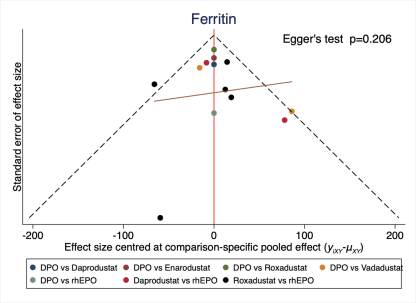

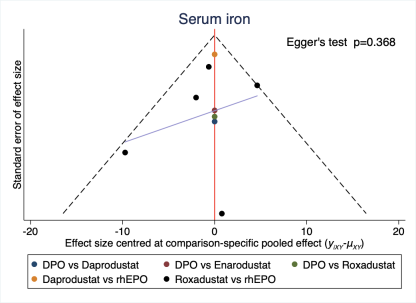

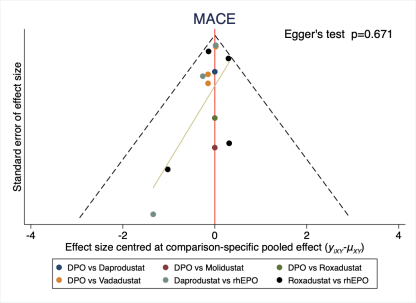

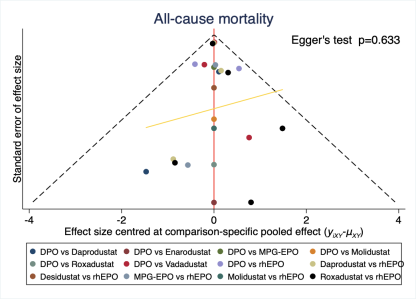


**Figue S7: Meta-analysis results of HIF-PHIs versus rhEPO and DPO for iron-related parameters**


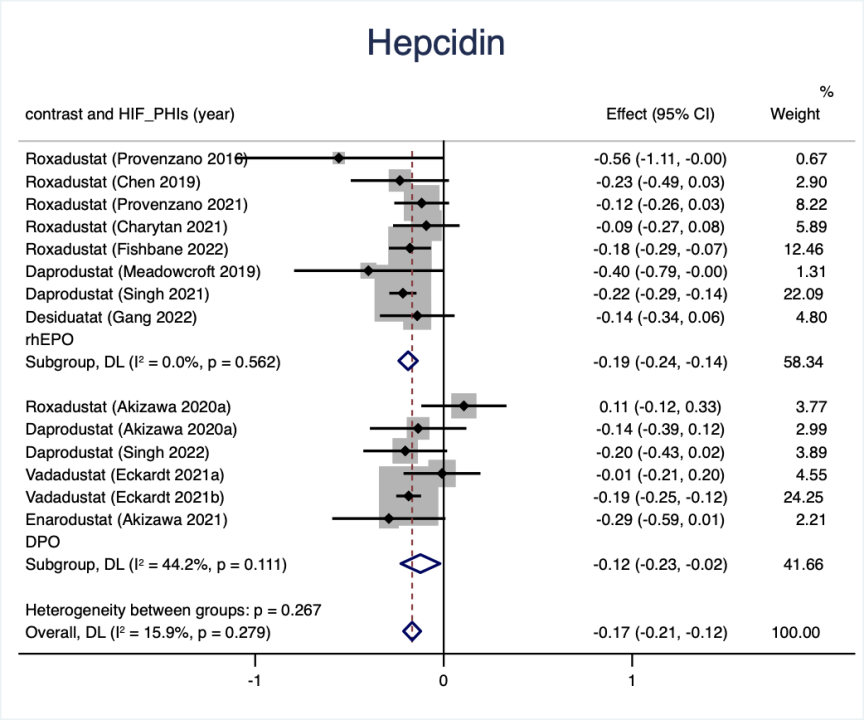


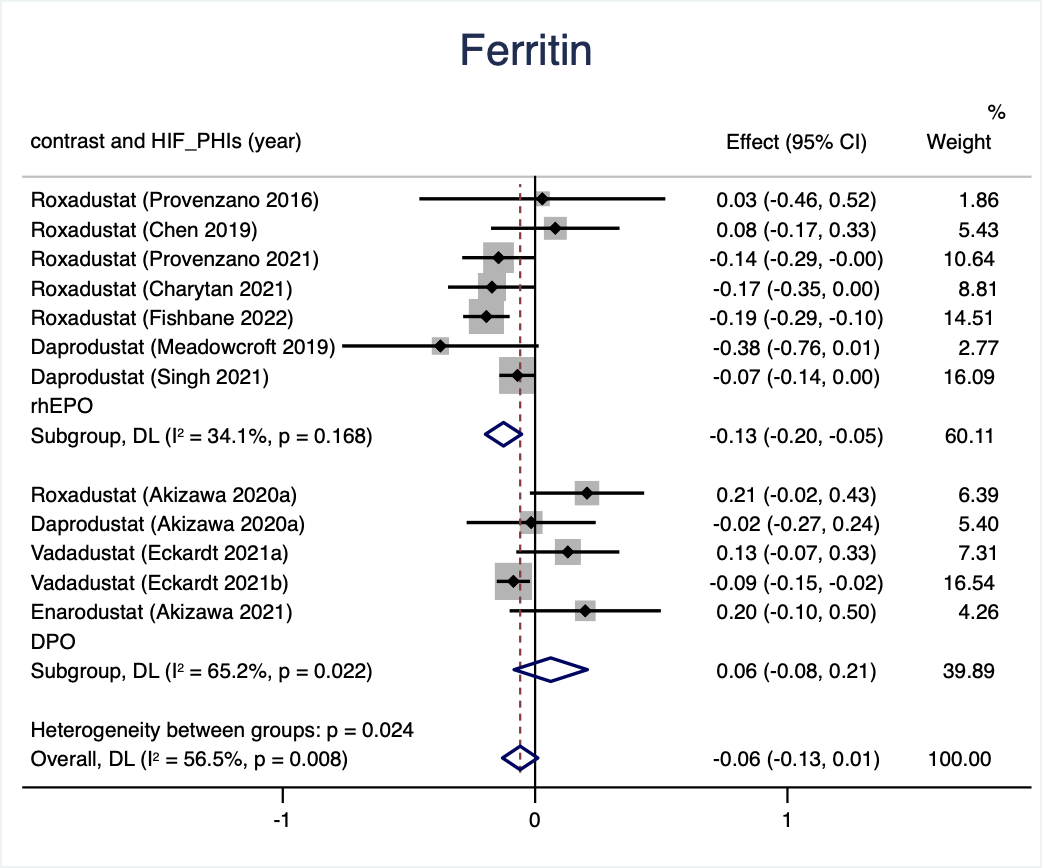


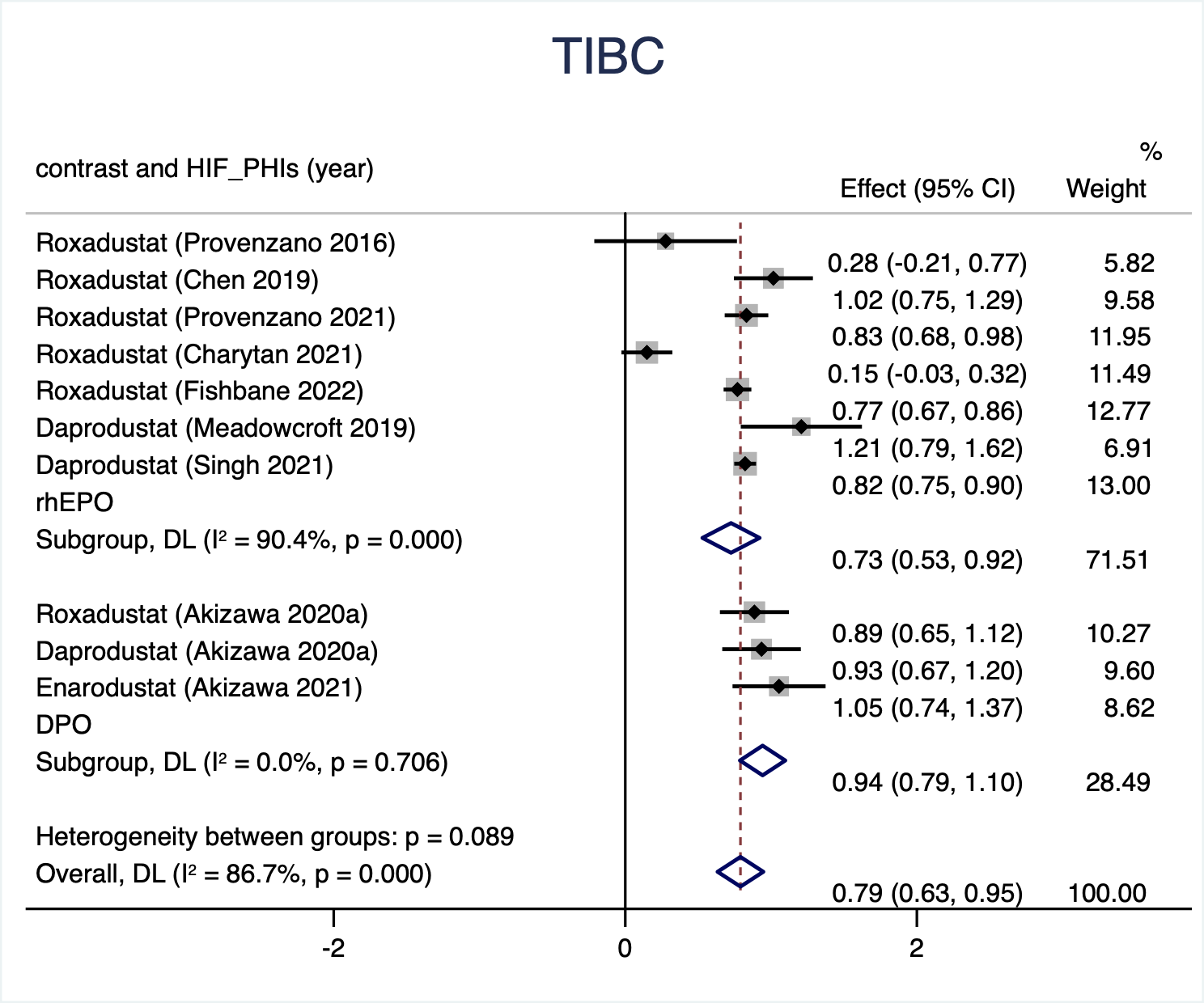

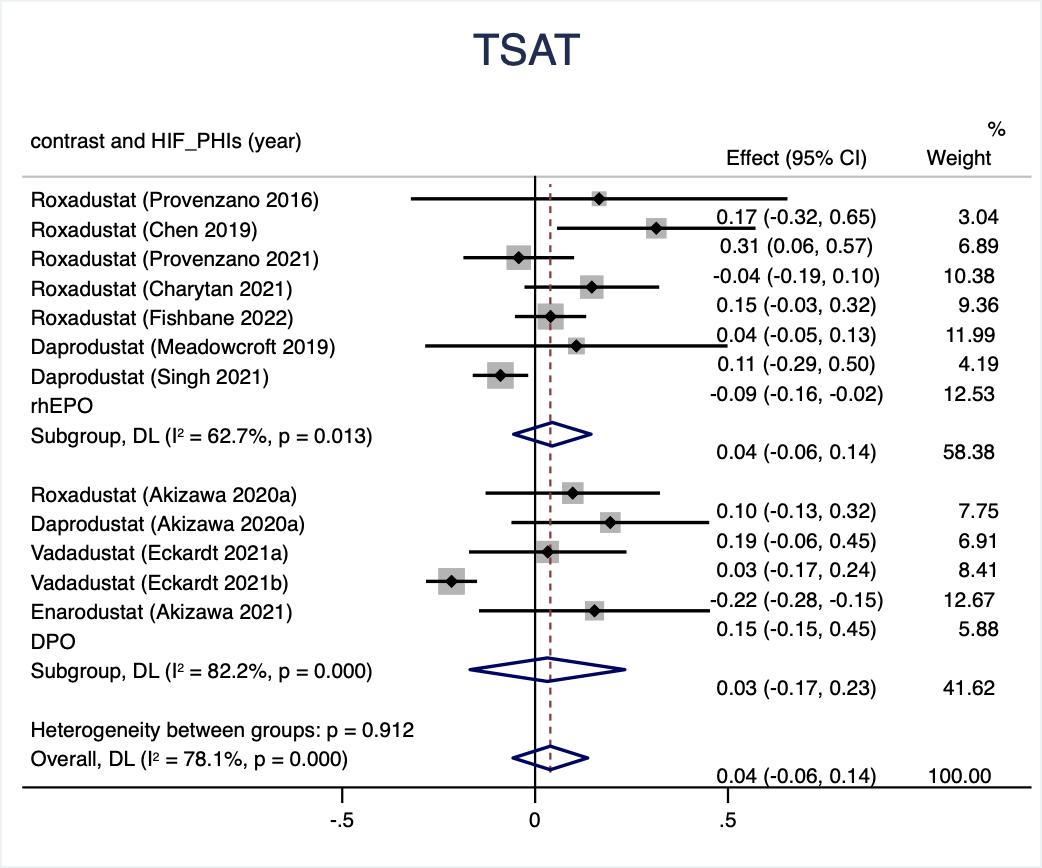

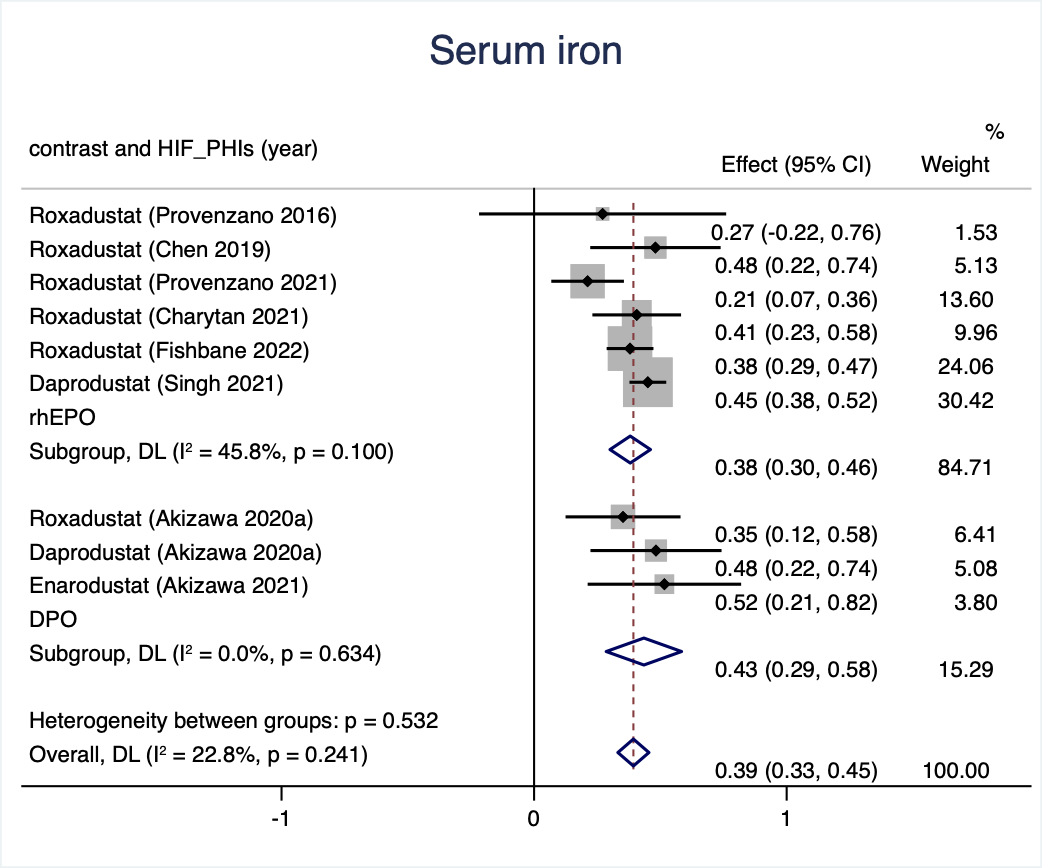


**Figue S8: Meta-analysis results of roxadustat versus rhEPO for CRP**


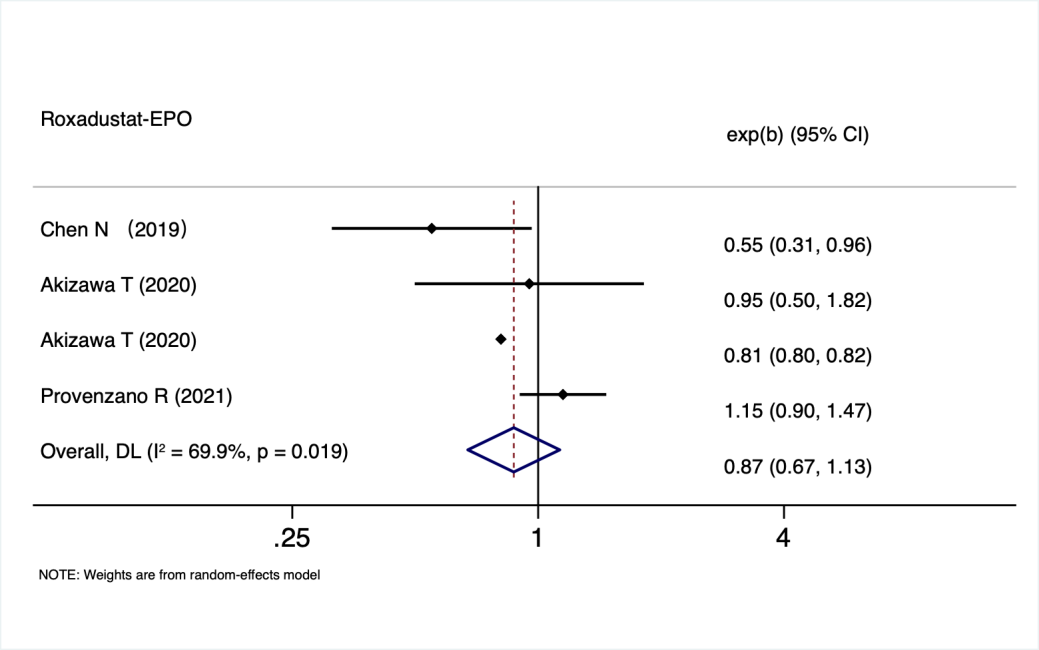


**Figue S9: Meta-analysis results of roxadustat versus rhEPO for LDL and total cholesterol**


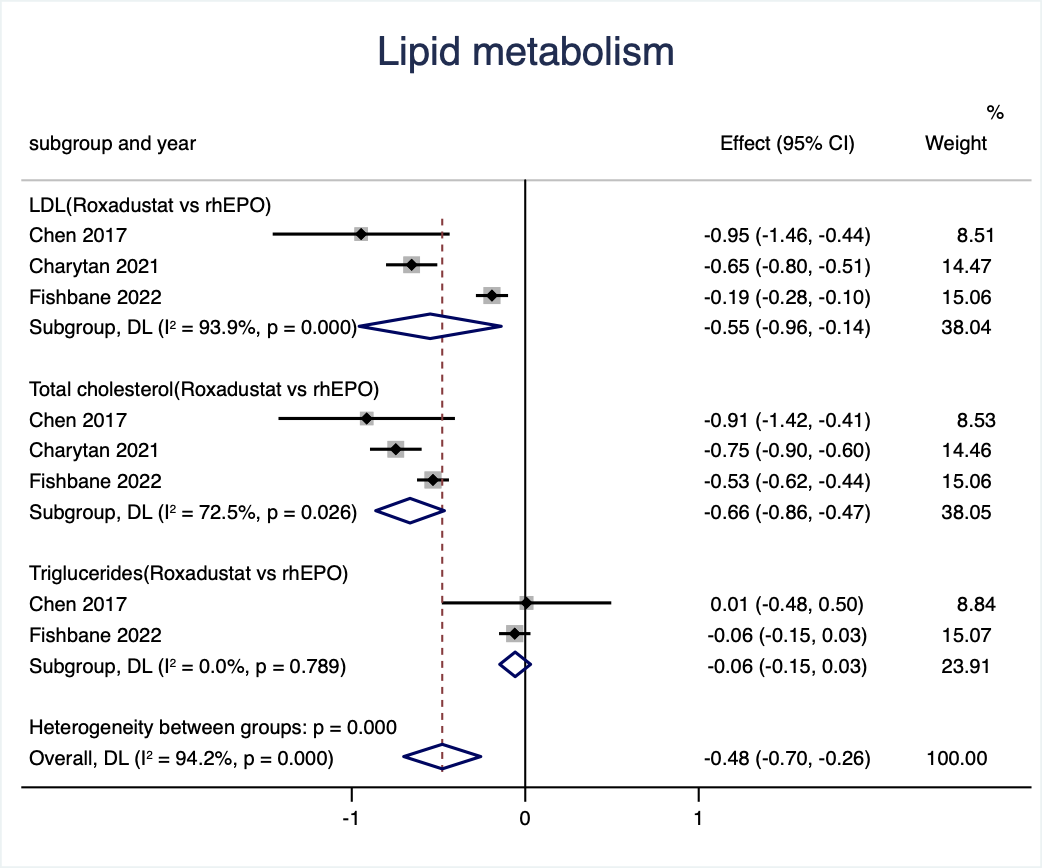


**Figue S10: Meta-analysis results of HIF-PHIs versus ESAs for gastrointestinal disorders and some other common AEs**


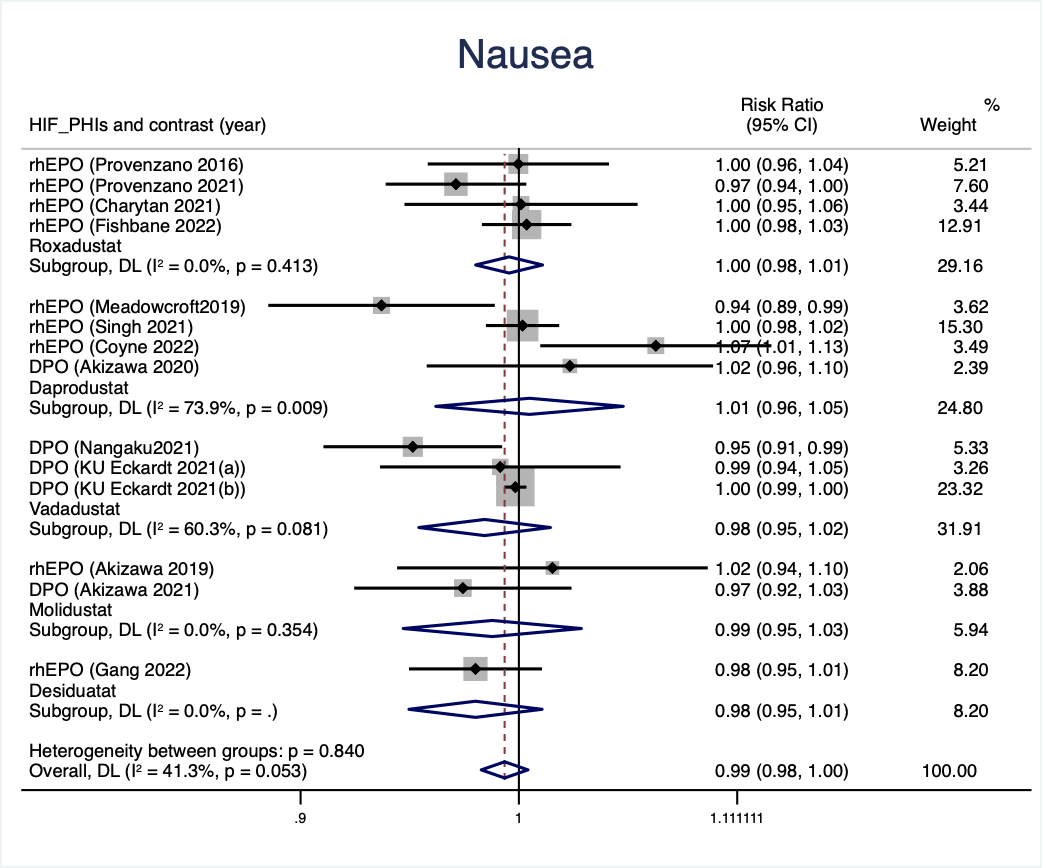

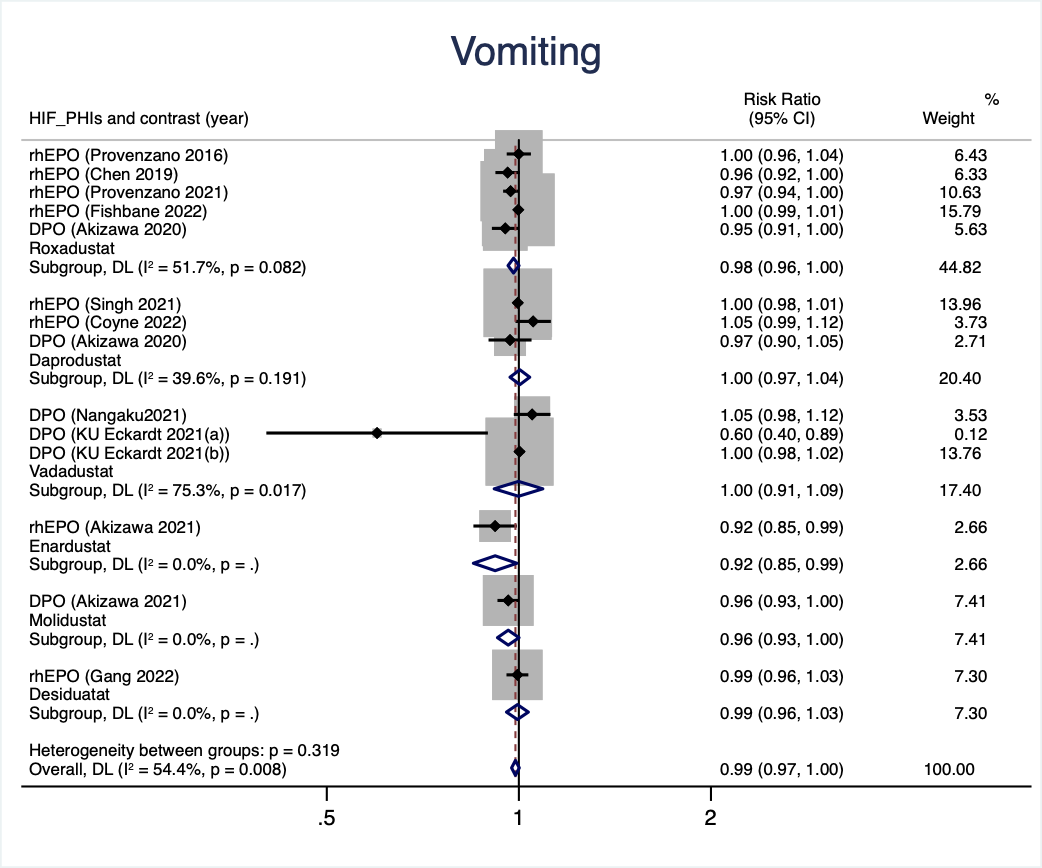


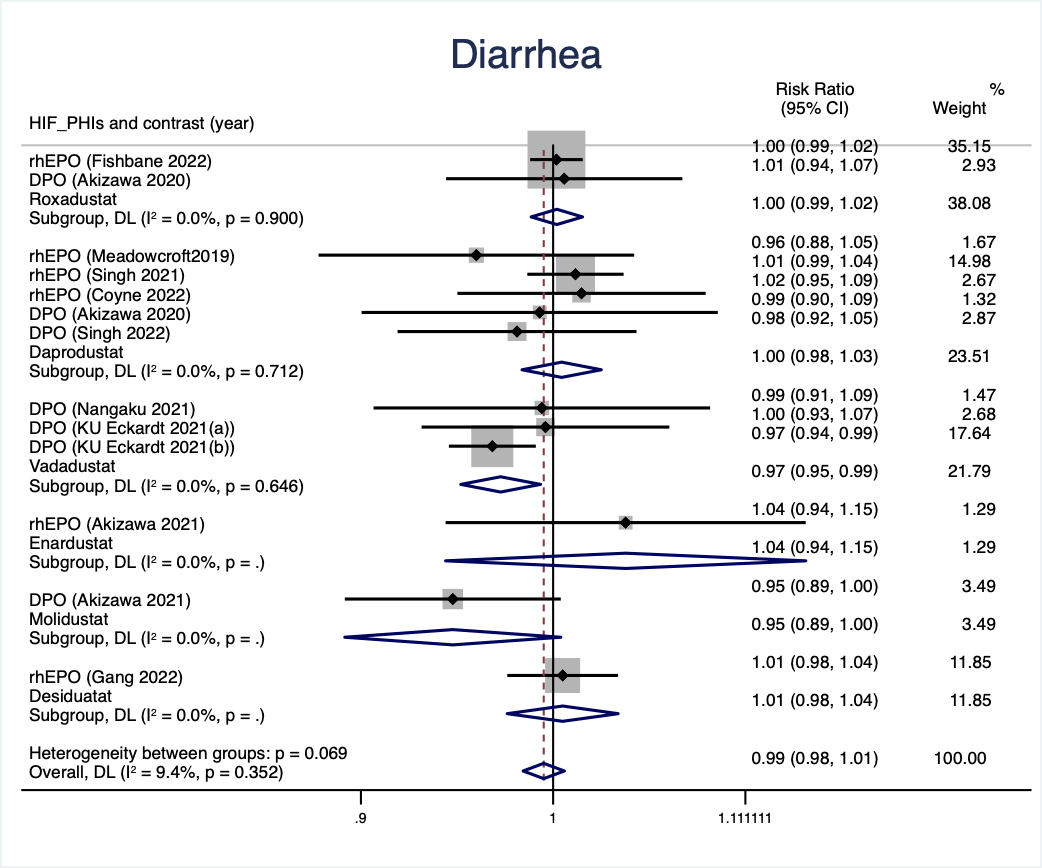

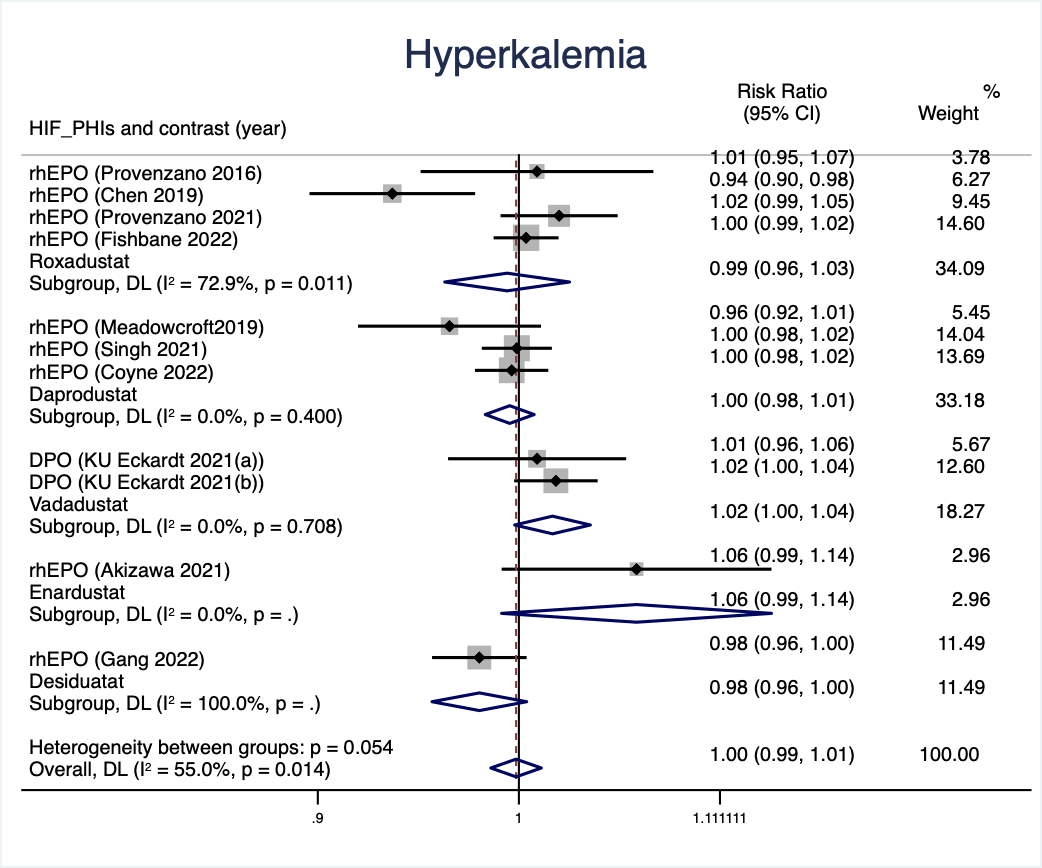


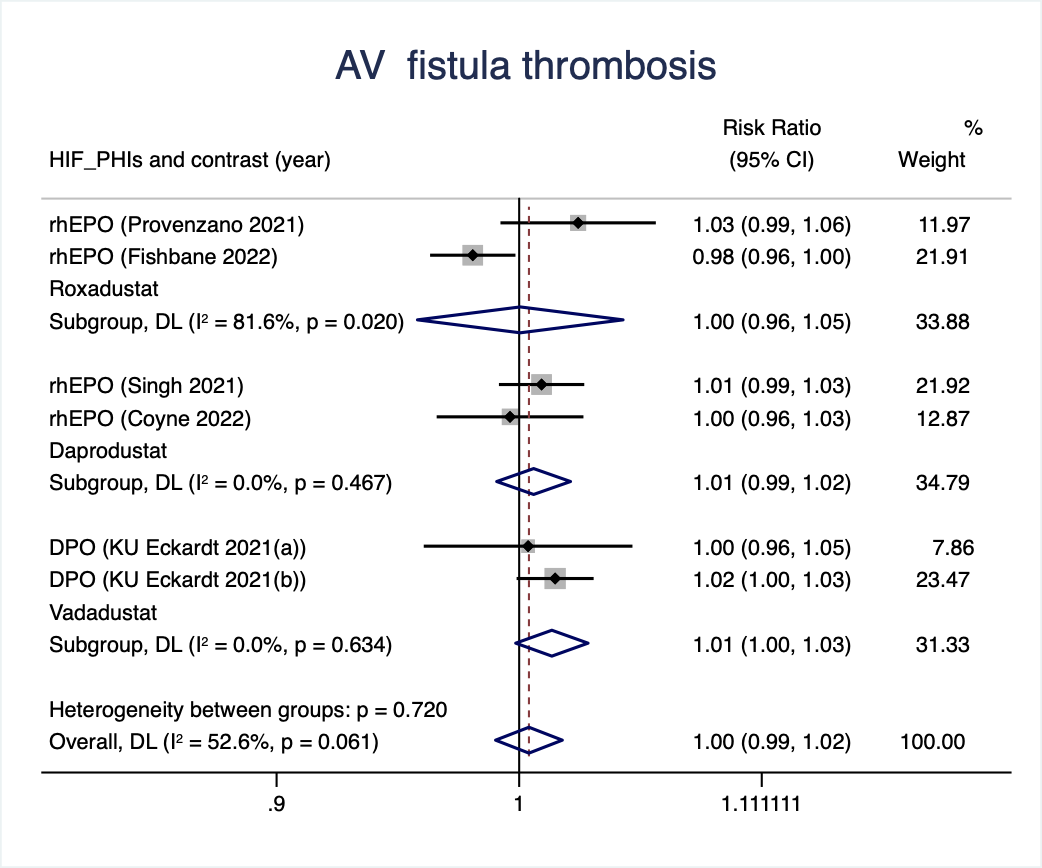


**Table S1: Search strategies (CENRAL, Cochrane Central Register of Controlled Trials, MEDLINE, Web of Science, Embase, PubMed, clinicaltrials.gov)**

| Database |  | Search term |
| --- | --- | --- |
| **CENTRAL <1990 to 2022 June 1>** | | |
|  | 1 | (kidney next (disease* or failure)) |
|  | 2 | ("chronic kidney" or "chronic renal" or CKD) |
|  | 3 | (renal next (impairment or insufficiency)) |
|  | 4 | "renal replacement therapy" |
|  | 5 | "End-Stage Kidney Disease ":ti,ab,kw |
|  | 6 | (hemodialysis or haemodialysis or dialysis) |
|  | 7 | Anemia or secondary Anemia |
|  | 8 | Prolyl-Hydroxylase Inhibitors or Proline Dioxygenase Inhibitor |
|  | 9 | or #1 to #8 |
|  | 10 | (Roxadustat or FG-4592):ti,ab,kw |
|  | 11 | (Daprodustatt or GSK1278863):ti,ab,kw |
|  | 12 | (Vadadustat AKB-6548):ti,ab,kw |
|  | 13 | (Molidustat or BAY 85-3934):ti,ab,kw |
|  | 14 | (desidustat or ZYAN1):ti,ab,kw |
|  | 15 | (enarodustat or JTZ-951):ti,ab,kw |
|  | 16 | (Erythropoietin or rhEPOgen or Eprex or Procrit or ormethoxy polyethylene glycol-epoetin beta or C.E.R.A.):ti,ab,kw |
|  | 17 | (darbepoetin alfa or NESP or Aranest or KRN 321):ti,ab,kw |
|  | 18 | or # 10 to #17 |
|  | 19 | randomly or Allocation, Random |
|  | 20 | randomized controlled trial |
|  | 21 | Controlled Clinical Trial |
|  | 22 | or # 19 to #21 |
|  | 23 | #9 and #18 and #22 with Publication Year from 1990 to 2022, in Trials |
| **Cochrane Central Register of Controlled Trials<1990 to 2022 June 1>** | | |
|  | 1 | (kidney next (disease* or failure)) |
|  | 2 | ("chronic kidney" or "chronic renal" or CKD) |
|  | 3 | (renal next (impairment or insufficiency)) |
|  | 4 | "renal replacement therapy" |
|  | 5 | "End-Stage Kidney Disease ":ti,ab,kw |
|  | 6 | (hemodialysis or haemodialysis or dialysis) |
|  | 7 | Anemia or secondary Anemia |
|  | 8 | or #1 to #8 |
|  | 9 | (Roxadustat or FG-4592):ti,ab,kw |
|  | 10 | (Daprodustatt or GSK1278863):ti,ab,kw |
|  | 11 | (Vadadustat AKB-6548):ti,ab,kw |
|  | 12 | (Molidustat or BAY 85-3934):ti,ab,kw |
|  | 13 | (desidustat or ZYAN1):ti,ab,kw |
|  | 14 | (enarodustat or JTZ-951):ti,ab,kw |
|  | 15 | (Erythropoietin or rhEPOgen or Eprex or Procrit or ormethoxy polyethylene glycol-epoetin beta or C.E.R.A.):ti,ab,kw |
|  | 16 | (darbepoetin alfa or NESP or Aranest or KRN 321):ti,ab,kw |
|  | 17 | or # 10 to #17 |
|  | 18 | #9 and #18 with Publication Year from 1990 to 2022, in Trials |
| **Web of Science<1990 to 2022 June 1>** | | |
|  | 1 | Kidney Diseases/ |
|  | 2 | Renal Insufficiency/ |
|  | 3 | exp Renal Insufficiency, Chronic/ |
|  | 4 | Renal Dialysis/ |
|  | 5 | Renal Replacement Therapy/ |
|  | 6 | exp Renal Dialysis/ |
|  | 7 | (chronic kidney or chronic renal or CKD).tw. |
|  | 8 | (hemodialysis or haemodialysis or dialysis).tw. |
|  | 9 | Anemia or secondary Anemia |
|  | 10 | Prolyl-Hydroxylase Inhibitors |
|  | 11 | End-Stage Kidney Disease |
|  | 12 | or 1-11 |
|  | 13 | (Roxadustat or FG-4592) |
|  | 14 | (Daprodustatt or GSK1278863) |
|  | 15 | (Vadadustat AKB-6548) |
|  | 16 | (Molidustat or BAY 85-3934) |
|  | 17 | (desidustat or ZYAN1) |
|  | 18 | (enarodustat or JTZ-951) |
|  | 19 | (Erythropoietin or rhEPOgen or Eprex or Procritor ormethoxy polyethylene glycol-epoetin beta or C.E.R.A.) |
|  | 20 | (darbepoetin alfa or NESP or Aranest or KRN 321) |
|  | 21 | or 13-20 |
|  | 22 | PBO.ab. |
|  | 23 | clinical trials as topic/ |
|  | 24 | double-blind procedure |
|  | 25 | or 22-24 |
|  | 26 | 12 and 21 and 25 |
| **MEDLINE <1990 to 2022 June 1>** | | |
|  | 1 | Kidney Diseases/ |
|  | 2 | Renal Insufficiency/ |
|  | 3 | exp Renal Insufficiency, Chronic/ |
|  | 4 | Renal Dialysis/ |
|  | 5 | Renal Replacement Therapy/ |
|  | 6 | exp Renal Dialysis/ |
|  | 7 | (chronic kidney or chronic renal or CKD).tw. |
|  | 8 | (hemodialysis or haemodialysis or dialysis).tw. |
|  | 9 | Anemia or secondary Anemia |
|  | 10 | Prolyl-Hydroxylase Inhibitors |
|  | 11 | End-Stage Kidney Disease |
|  | 12 | or 1-11 |
|  | 13 | (Roxadustat or FG-4592) |
|  | 14 | (Daprodustatt or GSK1278863) |
|  | 15 | (Vadadustat AKB-6548) |
|  | 16 | (Molidustat or BAY 85-3934) |
|  | 17 | (desidustat or ZYAN1) |
|  | 18 | (enarodustat or JTZ-951) |
|  | 19 | (Erythropoietin or epogen or Eprex or Procritor ormethoxy polyethylene glycol-epoetin beta or C.E.R.A.) |
|  | 20 | (darbepoetin alfa or NESP or Aranest or KRN 321) |
|  | 21 | or 13-20 |
|  | 22 | randomized controlled trial.pt. |
|  | 23 | controlled clinical trial.pt. |
|  | 24 | pragmatic clinical trial.pt. |
|  | 25 | randomi#ed.tw. |
|  | 26 | randomly.ab. |
|  | 27 | (crossover or cross-over).tw. |
|  | 28 | Cross-over Studies/ |
|  | 29 | trial.ti. |
|  | 30 | or/22-28 |
|  | 31 | animals/ not (humans/ and animals/) |
|  | 32 | 12 and 21 and 30 |
| **Embase <1990 to 2022 June 1>** | | |
|  | 1 | Kidney Disease |
|  | 2 | Kidney Failure |
|  | 3 | exp Chronic Kidney Failure |
|  | 4 | End Stage Renal Failure |
|  | 5 | Mild Renal Impairment |
|  | 6 | Moderate Renal Impairment |
|  | 7 | Renal Replacement Therapy-Dependent Renal Disease |
|  | 8 | Severe Renal Impairment |
|  | 9 | exp Renal Replacement Therapy |
|  | 10 | (chronic kidney or chronic renal or CKD) |
|  | 11 | (hemodialysis or haemodialysis or dialysis) |
|  | 12 | Anemia or secondary Anemia |
|  | 13 | Proline Hydroxylase Inhibitors |
|  | 14 | End-Stage Kidney Disease |
|  | 15 | or 1-14 |
|  | 16 | (Roxadustat or FG-4592) |
|  | 17 | (Daprodustatt or GSK1278863) |
|  | 18 | (Vadadustat AKB-6548) |
|  | 19 | (Molidustat or BAY 85-3934) |
|  | 20 | (desidustat or ZYAN1) |
|  | 21 | (enarodustat or JTZ-951) |
|  | 22 | (Erythropoietin or rhEPO or Eprex or Procritor ormethoxy polyethylene glycol-epoetin beta or C.E.R.A.) |
|  | 23 | (darbepoetin alfa or NESP or Aranest or KRN 321) |
|  | 24 | or 16-23 |
|  | 25 | randomized controlled trial/ |
|  | 26 | crossover procedure/ |
|  | 27 | double-blind procedure/ |
|  | 28 | single-blind procedure/ |
|  | 29 | or 25- 28 |
|  | 30 | 15 and 24 and 29 |
| **clinicaltrials.gov <1990 to 2022 June 1>** | | |
|  | 1 | Kidney Disease |
|  | 2 | Kidney Failure |
|  | 3 | exp Chronic Kidney Failure |
|  | 4 | End Stage Renal Failure |
|  | 5 | Mild Renal Impairment |
|  | 6 | Moderate Renal Impairment |
|  | 7 | Renal Replacement Therapy-Dependent Renal Disease |
|  | 8 | Severe Renal Impairment |
|  | 9 | exp Renal Replacement Therapy |
|  | 10 | (chronic kidney or chronic renal or CKD) |
|  | 11 | (hemodialysis or haemodialysis or dialysis) |
|  | 12 | Anemia or secondary Anemia |
|  | 13 | Proline Hydroxylase Inhibitors |
|  | 14 | End-Stage Kidney Disease |
|  | 15 | or 1-14 |
|  | 16 | (Roxadustat or FG-4592) |
|  | 17 | (Daprodustatt or GSK1278863) |
|  | 18 | (Vadadustat AKB-6548) |
|  | 19 | (Molidustat or BAY 85-3934) |
|  | 20 | (desidustat or ZYAN1) |
|  | 21 | (enarodustat or JTZ-951) |
|  | 22 | (Erythropoietin or rHurhEPO or Eprex or Procritor ormethoxy polyethylene glycol-epoetin beta or C.E.R.A.) |
|  | 23 | (darbepoetin alfa or NESP or Aranest or KRN 321) |
|  | 24 | or 16-23 |
|  | 25 | PBO |
|  | 26 | double or blind |
|  | 27 | singl or blind |
|  | 28 | randomized controlled trial |
|  | 29 | randomly |
|  | 30 | random |
|  | 31 | or 25-30 |
|  | 32 | 15 and 24 and 31 |

| **Table S2: Trials included in the systematic review.** | | | | | | | | | | | | | | | |
| --- | --- | --- | --- | --- | --- | --- | --- | --- | --- | --- | --- | --- | --- | --- | --- |
| Study ID | **Trial registration** | **Study duration** | **Phase of study,country (no. of centers)** | **Study type** | **Type of patient** | **Treatment** | **Comparison** | **No. of participants(Treatment/ Comparison)** | **Gender(%male)** | **RACE** | **Mean age(SD)** | **baseline Hb** | **Dose** | **Iron supplement** | **Hb response rate** |
| Provenzano 2016 | NCT01147666 | 19 weeks | Phase 2, - | Randomized, open-label active comparator,dose-ranging study | Stable HD | roxadustat | rhEPO | 61/22 | 67%/61% | Global | 56.9 ± 12.1/ 57.0 ± 11.6 | 11.2 ± 0.7/ 11.2 ± 1.0 | with oral roxadustat doses fixed at 1.0, 1.5, 1.8, or 2.0 mg/kg tiw. | Allowed oral iron, i.v. iron (Rescue therapy) | achieving a hemoglobin level ≥11 g/dL |
| Chen 2019 | NCT02652806 | 26 weeks | Phase 3,- | Randomized,open-label | Stable HD+PD | roxadustat | rhEPO | 204/101 | 61.8%/58.0% | Asians | 47.6±11.7/ 51.0±11.8 | 10.4 ± 0.7/ 10.5 ± 0.7 | Initial dose: 100 mg (weight 45–60 kg) or 120 mg (weight ≥60 kg) tiw.Dose adjustments every 4 weeks | Allowed oral iron, i.v. iron (Rescue therapy) | achieving a hemoglobin level ≥10 g/dL |
| Akizawa 2020 | NCT02952092 | 24 weeks | Phase 3, Japan (58) | Randomized, 2-arm parallel, double-blind | Stable HD | roxadustat | DPO | 151/152 | 67.3%/70.9% | Asians | 64.6±11.7/ 64.9±10.1 | 11.02 ± 0.56/ 11.01 ± 0.60 | Initial dose: 70 mg or 100 mg tiw. Dose adjustments every 4 weeks | Allowed oral iron, i.v. iron (Rescue therapy) | Hb within target range(10–12 g/dl) |
| Crisky 2021(a) | NCT02278341 | 52–104 weeks | Phase 3, worldwide (150) | Randomized, open-label | Stable HD+PD  Stable HD+PD | roxadustat | rhEPO | 256/257 | 57.8%/53.3% | Global | 61.0±13.6/ 61.9±14.0 | 10.78± 0.63/ 10.80 ± 0.61 | Initial dose: 100 mg, 150 mg or 200 mg tiw. Dose adjustments every 4 weeks | Encouraged oral iron, i.v. iron (Rescue therapy) | Hb within target range(10–12 g/dl) |
| Crisky 2021(b) |  |  |  |  |  | roxadustat | DPO | 158/163 | 61.4%/60.1% | Global | 61.1±14.3/61.8±12.6 | 10.68± 0.59/ 10.73 ± 0.61 |  |  |  |
| Provenzano 2021 | NCT02052310 | 52 weeks- 4 years | Phase 3, worldwide (113) | Randomized, open-label | Incident HD+PD | roxadustat | rhEPO | 552/521 | 59.2%/58.9% | Global | 58.3±14.7/54.3±14.6 | 8.43 ± 1.044/ 8.46 ± 0.964 | Initial dose: 70 mg (weight <70 kg) or 100 mg (weight ≥70 kg) tiw | Encouraged oral iron, i.v. iron (Rescue therapy) | achieving a hemoglobin level ≥11 g/dL |
| Charytan 2021 | NCT02273726 | 52 weeks | Phase 3, US-based sites（76） | Randomized, open-label, | Stable HD+PD | roxadustat | rhEPO | 370/371 | 50.5%/58.0% | Global | 57.6±13.6/58.4±13.3 | 10.30± 0.66/ 10.30± 0.66 | 70, 100, 150,or 200mg tiw | Encouraged oral iron, i.v. iron (Rescue therapy) | Hb within target range(10–12g/dl) |
| Fishbane S 2022 | NCT02174731 | 52 weeks | Phase 3, worldwide (197) | Randomized, open-label,active-control | Stable HD+PD | roxadustat | rhEPO | 1068/1065 | 59.5%/59.3% | Global | 53.5±15.3/54.5±15.0 | 10.20± 1.18/ 10.30± 1.33 | 70, 100, 150,or 200mg tiw | Encouraged oral iron, i.v. iron (Rescue therapy) | Hb within target range(10–12g/dl) |
| Meadowcroft2019 | NCT01977482 | 24 weeks | Phase 2, Global study(107) | Randomized,double-blinded, dose-ranging | Stable HD | daprodustat | rhEPO | 171/39 | 63%/67% | Global | 59.6 ± 13.3/ 59.7 ± 18.7 | 10.39 ± 0.66/ 10.55 ± 0.94 | a fixed-dose of 4, 6, 8 10,12 mg, dose adjustments every 4 weeks | Encouraged receiving IV iron (Rescue therapy) | Hb within target range(10–11.5g/dl) |
| Akizawa T 2020 | NCT02969655 | 52 weeks | Phase 3，Japan (50) | Randomized,double-blind,active-control | Stable HD | daprodustat | DPO | 136/135 | 67%/66% | Asians | 64.1±10.3/ 63.5 ±10.54 | 10.94±0.77/ 10.82 ± 0.73 | 1, 2, 4, 6, 8, 12, 18, and 24 mg once daily | Encouraged receiving IV iron (Rescue therapy) | Hb within target range(10–12g/dl) |
| Singh 2021 | NCT02879305 | 52 weeks | Phase 3, worldwide ( 431) | Randomized, open-label (sponsor blind),active-control | Stable HD+PD | daprodustat | rhEPO | 1487/1477 | 57.2%/57.3% | Global | 58±14.07/ 59±15.56 | 10.35±0.97/ 10.39 ± 0.98 | 1, 2, 4, 6, 8, 10, 12, 16, and 24 mg once daily | Encouraged receiving IV iron (Rescue therapy) | Hb within target range(10–11g/dl) |
| Singh 2022 | NCT03029208 | 52 weeks | Phase 2, Global study(18) | Randomized, open-label,active-control | Incident HD+PD | daprodustat | DPO | 157/155 | 62%//61% | Global | 52±13.3/ 52±16.3 | 9.5±1.0/ 9.5±1.0 | 1, 2, 4, 6, 8, and 10mg once daily Dose adjustments through scheme | Supplemental iron therapy if ferritin ≤100 ng/mL or TSAT ≤20% | Hb within target range(10–11g/dl) |
| Coyne 2022 | NCT03400033 | 53 weeks | Phase 3, 13 countries（90） | Randomized,double-blinded, active-controlled, double-dummy, parallel-group, | Stable HD | daprodustat | rhEPO | 270/137 | 55%/59% | Global | 60.0±14.07/ 56.0±12.59 | 10.44 ±0.83/ 10.59± 0.93 | 2-48mg tiw | Supplemental iron therapy if ferritin ≤100 ng/mL or TSAT ≤20% | Hb within target range(10–11.5g/dl) |
| Nangaku2021 | NCT03439137 | 52 weeks | Phase 3 Japan，multicenter | Randomized, double blind | Stable HD | vadadustat | DPO | 162/161 | 64.2%/67.7% | Asians | 66.0±11.3/ 64.7 ± 11.7 | 10.73 ±0.7/ 10.73± 0.7 | Starting dose: 300 mg q.d. Maintenance dose:150–600 mg qd. | Iron supplementation was utilized to maintain a se_100 ng/mL or TSAT of 20%. | Hb within target range(10–12g/dl) |
| Eckardt 2021(a) | NCT02865850 | 52 weeks | Phase 3 Global study | Randomized, open label,active-control | Incident HD+PD | vadadustat | DPO | 181/188 | 59.1%/60.1% | Global | 56.5±14.8/ 55.6±14.6 | 9.4±1.1/ 9.4± 1.1 | Starting dose: 300 mg q.d. Maintenance dose:150–600 mg qd. | Iron supplementation was utilized to maintain a serum ferritin level of 100 ng/mL or TSAT of 20%. | Hb within target range(10–12g/dl) |
| Eckardt 2021(b) | NCT02892149 | 52 weeks | Phase 3 Global study | Randomized, open label,active-control | Stable HD+PD | vadadustat | DPO | 1777/1777 | 55.7%/56.5% | Global | 57.9±13.9/ 58.4±13.8 | 10.6±0.9/ 10.2±0.8 | Starting dose: 300 mg q.d. Maintenance dose:150–600 mg qd. | Iron supplementation was utilized to maintain a serum ferritin level of 100 ng/mL or TSAT of 20%. | Hb within target range(10–12g/dl) |
| Akizawa 2021 | JapicCTI-152881 | 24 weeks | Phase 3 Japan | Randomized, double-blind，active-controlled | Stable HD | enarodustat | DPO | 86/86 | 70.9%/70.9% | Asians | 63.2±10.8/ 64.8 ±10.3 | 10.79±0.65/ 10.87±0.7 | Initial doses were 4 mg/day | Iron supplementation was utilized to maintain a serum ferritin level of 100 ng/mL or TSAT of 20%. | Hb within target range(10–12g/dl) |
| Akizawa 2019 | NCT02064426 | 52 weeks | Phase 3, United States and Japan | a controlled, parallel group, open-label | Stable HD | molidustat | rhEPO | 57/30 | 58%/77% | Global | 61.0±12/ 59.0±9 | 10.4±0.7/ 10.5±0.5 | 15, 25,50, 75, 100, and 150 mg once daily | Encouraged oral iron, i.v. iron | Hb within target range(10–12g/dl) |
| Akizawa 2021 | NCT03543657 | 52 weeks | Phase 3, Japan(53) | Randomized, double blinded, double-dummy | Stable HD | molidustat | DPO | 153/76 | 59.5%/64.5% | Asians | 66.2±10.3/ 64.8±10.6 | 10.77±0.64/ 10.84±0.65 | a starting dose of 75 mg/day. multiple doses of 5, 12.5, 25, and 50 mg once daily | Encouraged receiving IV iron (Rescue therapy) | Hb within target range(10–12g/dl) |
| Gang2022 | CTRI/2019/12/022312 | 24 weeks | Phase 3, India(38) | Randomized, open label,active-control | Stable HD | desidusttat | rhEPO | 196/196 | 68.8%/68.4% | Asians | 51.0±14.0/ 50.9±13.5 | 9.61±1.0/ 9.55± 1.4 | 100 mg tiw. dose could be adjusted anytime | Encouraged receiving IV iron (Rescue therapy) | Hb within target range(10–12g/dl) |
| Vanrenterghem 2002 | - | 52 weeks | Phase 3，European and Australian | Randomized, double-blind | Stable HD+PD | rhEPO | DPO | 374/175 | 54%/57% | Global | 60.1(11-88)/ 60.9(22-87) | 11(9.5-12.5)/ 11(9.5-12.5) | Epo tiw or biw to once weekly of darbepoetin alfa(200 IU EPO =1 ug darbepoetin alfa) | Encouraged oral iron, i.v. iron | Hb within target range(9.5–12.5 g/dL) |

**Table S3: Inconsistency of different intervention: side-splitting inconsistency**

| **Hb response** | | | | | | | | |
| --- | --- | --- | --- | --- | --- | --- | --- | --- |
| A G | -0.1265827 | 0.2033563 | 0.6870724 | 0.5435285 | -0.8136552 | 0.5811505 | 0.161 | 0.3726173 |
| A H | 0.4638212 | 0.481431 | -0.3498467 | 0.3255321 | 0.8136679 | 0.58116 | 0.161 | 0.3726188 |
| B G | -0.3554453 | 0.3486325 | 0.4672713 | 0.6077951 | -0.8227166 | 0.6992353 | 0.239 | 0.3761765 |
| B H | 0.296571 | 0.5566703 | -0.5261468 | 0.4231415 | 0.8227178 | 0.6992357 | 0.239 | 0.3761762 |
| C H | 0.3103191 | 0.2502917 | -0.5084961 | 365.9342 | 0.8188152 | 365.9343 | 0.998 | 0.3847642 |
| D H | 0.7456877 | 0.5877567 | -0.9449937 | 633.5239 | 1.690681 | 633.5245 | 0.998 | 0.3847642 |
| E H | 0.7547051 | 0.5584761 | -0.9547279 | 630.2449 | 1.709433 | 630.2454 | 0.998 | 0.3847648 |
| F H | -0.4382202 | 0.4392479 | 0.2390719 | 630.8455 | -0.6772921 | 630.8458 | 0.999 | 0.3847646 |
| G H | -0.158316 | 0.3462075 | 0.0093619 | 0.3552106 | -0.1676779 | 0.4985095 | 0.737 | 0.4204469 |
| G I | -0.0630945 | 0.2001032 | 1.169666 | 0.2084135 | -1.23276 | 0.2889247 | 0 | 4.04E-08 |
| H I | 0.9673957 | 0.1862348 | -0.2653648 | 0.2208939 | 1.23276 | 0.2889247 | 0 | 3.81E-07 |
| **RBC transfusion** | | | | | | | | |
| A G | 0.3233544 | 0.1300476 | 0.1502153 | 95.11367 | 0.1731391 | 95.11378 | 0.999 | 0.1199338 |
| B G | 0.6210907 | 0.3485858 | 0.3823948 | 0.4630736 | 0.2386959 | 0.579497 | 0.68 | 0.1460398 |
| B H | 0.1907847 | 0.3730404 | 0.4294798 | 0.4434612 | -0.2386951 | 0.5794972 | 0.68 | 0.1460399 |
| C H | -0.0877175 | 0.2776501 | 0.2434033 | 363.935 | -0.3311208 | 363.9353 | 0.999 | 0.1199335 |
| E H | -0.926143 | 1.560146 | 1.081841 | 632.0474 | -2.007984 | 632.0531 | 0.997 | 0.1199319 |
| G H | -0.1244708 | 0.3412873 | -0.366618 | 0.342701 | 0.2421472 | 0.4836538 | 0.617 | 0.1463744 |
| G I | -0.0824515 | 0.3576148 | 0.0122133 | 0.416855 | -0.0946647 | 0.5492553 | 0.863 | 0.1572519 |
| H I | 0.2312417 | 0.3019411 | 0.1365756 | 0.4588175 | 0.0946661 | 0.5492558 | 0.863 | 0.1572521 |
| **Discontinuation** | | | | | | | | |
| A G | -0.4670106 | 0.1509287 | -0.4738301 | 0.4170636 | 0.0068195 | 0.4394521 | 0.988 | 0.2240808 |
| A H | -0.5180633 | 0.3811144 | -0.5112352 | 0.2188023 | -0.0068281 | 0.4394572 | 0.988 | 0.2240809 |
| B G | -0.198495 | 0.1929797 | -0.2377704 | 0.3221125 | 0.0392754 | 0.371013 | 0.916 | 0.2261259 |
| B H | -0.2734866 | 0.2666664 | -0.234212 | 0.2579913 | -0.0392746 | 0.3710132 | 0.916 | 0.2261258 |
| C H | -0.5849632 | 0.1671061 | -0.4397222 | 365.984 | -0.145241 | 365.9842 | 1 | 0.2068154 |
| D H | -0.3002609 | 0.5996637 | -0.7205504 | 630.8212 | 0.4202895 | 630.822 | 0.999 | 0.2068131 |
| E G | -0.634056 | 2.023184 | -0.509633 | 0.4580639 | -0.124423 | 2.074392 | 0.952 | 0.2076943 |
| E H | -0.5611554 | 0.4318293 | -0.685571 | 2.028904 | 0.1244157 | 2.074351 | 0.952 | 0.2076943 |
| F G | -0.0606152 | 0.3215673 | -0.855678 | 631.5585 | 0.7950628 | 631.5587 | 0.999 | 0.2068137 |
| G H | -0.0335564 | 0.1977704 | -0.0632989 | 0.2562391 | 0.0297425 | 0.3194716 | 0.926 | 0.2256871 |
| G I | -0.5564343 | 0.2136377 | 0.3654743 | 364.0358 | -0.9219086 | 364.0358 | 0.998 | 0.2068129 |
| **AEs in discontinuation** | | | | | | | | |
| A G | -0.5060768 | 0.1679234 | -0.1023995 | 0.5903047 | -0.4036773 | 0.6066225 | 0.506 | 0.1841206 |
| A H | -0.4410561 | 0.5064319 | -0.8447392 | 0.3339629 | 0.4036832 | 0.6066338 | 0.506 | 0.1841211 |
| B G | -0.0146632 | 0.1746598 | -0.5659809 | 0.4850043 | 0.5513177 | 0.5135288 | 0.283 | 0.1698522 |
| B H | -0.5697775 | 0.3421347 | -0.0184594 | 0.3833622 | -0.5513182 | 0.5135302 | 0.283 | 0.1698527 |
| C H | -0.7131137 | 0.2144041 | -0.7343515 | 364.0061 | 0.0212378 | 364.0064 | 1 | 0.171084 |
| D H | -0.2876821 | 0.7979343 | -1.160563 | 633.393 | 0.8728805 | 633.3945 | 0.999 | 0.1710832 |
| E G | -1.419812 | 0.81666 | -2.467637 | 1.482939 | 1.047825 | 1.692941 | 0.536 | 0.1776793 |
| E H | -2.687905 | 1.45802 | -1.64003 | 0.8603862 | -1.047874 | 1.692954 | 0.536 | 0.1776789 |
| G H | -0.3637634 | 0.5228007 | -0.1947319 | 0.3172181 | -0.1690315 | 0.6115127 | 0.782 | 0.1964185 |
| G I | 1.27731 | 0.9107964 | -1.242051 | 0.7547254 | 2.519362 | 1.183157 | 0.033 | 0.1371841 |
| **Death in discontinuation** | | | | | | | | |
| A G | -0.3204872 | 1.24622 | -1.327506 | 1.666223 | 1.007019 | 2.082719 | 0.629 | 0.3672237 |
| A H | -1.629439 | 1.596311 | -0.621929 | 1.337834 | -1.00751 | 2.082788 | 0.629 | 0.367251 |
| B G | 0.067037 | 0.3004919 | -1.426787 | 449.0623 | 1.493824 | 449.0568 | 0.997 | 0.2630961 |
| C H | 0.2347719 | 0.287736 | -2.308345 | 654.6159 | 2.543117 | 654.6159 | 0.997 | 0.2631497 |
| E G | -1.025495 | 1.138318 | 1.217659 | 1.119144 | -2.243153 | 1.596326 | 0.16 | 0.2050785 |
| E H | 0.713211 | 1.030626 | -1.529958 | 1.219042 | 2.243169 | 1.596327 | 0.16 | 0.2050783 |
| F G | 0.5753641 | 0.6874506 | -1.944281 | 634.1022 | 2.519645 | 634.1032 | 0.997 | 0.2631193 |
| G H | -0.2356444 | 0.7329366 | -0.4628093 | 0.9066222 | 0.227165 | 1.165831 | 0.846 | 0.5940036 |
| G I | -0.9988901 | 0.7091027 | 0.1156059 | 0.5781984 | -1.114496 | 0.914953 | 0.223 | 0.000012 |
| H I | 0.2540642 | 0.413449 | -0.860422 | 0.8162113 | 1.114486 | 0.9149539 | 0.223 | 3.41E-06 |
| **Refusal of treatment in discontinuation** | | | | | | | | |
| A G | -0.4976228 | 0.1411542 | 0.242596 | 0.9108089 | -0.7402188 | 0.9216818 | 0.422 | 5.87E-07 |
| A H | -0.2368424 | 0.6809547 | -0.9770738 | 0.6211689 | 0.7402314 | 0.9217104 | 0.422 | 9.75E-08 |
| B G | 0.0638983 | 0.092162 | -0.3638967 | 1.059986 | 0.4277951 | 1.063985 | 0.688 | 2.96E-08 |
| B H | -0.4205044 | 0.92107 | 0.0072855 | 0.5326474 | -0.4277899 | 1.063994 | 0.688 | 2.96E-08 |
| C H | -0.7954439 | 0.1194509 | -0.5141562 | 446.9343 | -0.2812877 | 446.9343 | 0.999 | 0.0000105 |
| E G | -1.025493 | 1.119692 | 0.9955764 | 1.497437 | -2.021069 | 1.86977 | 0.28 | 4.15E-09 |
| E H | 0.7063878 | 1.421233 | -1.314707 | 1.214974 | 2.021095 | 1.869779 | 0.28 | 3.04E-07 |
| G I | -0.5927942 | 0.7122504 | 0.56016 | 0.7540969 | -1.152954 | 1.037285 | 0.266 | 8.39E-07 |
| H I | 0.4180955 | 0.5349757 | -0.7348561 | 0.8886883 | 1.152952 | 1.037288 | 0.266 | 2.59E-08 |
| **Content withdrawn in discontinuation** | | | | | | | | |
| A G | -0.1585636 | 0.2772462 | -0.8531639 | 0.8546455 | 0.6946003 | 0.8951518 | 0.438 | 0.3268533 |
| A H | -0.5872037 | 0.7165796 | 0.1074348 | 0.5365286 | -0.6946385 | 0.8951811 | 0.438 | 0.3268538 |
| B G | -0.1308639 | 0.272502 | -0.9307521 | 1.036363 | 0.7998883 | 1.067532 | 0.454 | 0.336099 |
| B H | -0.7157885 | 0.9370479 | 0.0841098 | 0.5114525 | -0.7998983 | 1.06754 | 0.454 | 0.3361011 |
| C H | -1.088811 | 0.3482939 | 0.8102026 | 365.0449 | -1.899013 | 365.0456 | 0.996 | 0.3247496 |
| D H | -1.098612 | 1.6719 | 0.8201589 | 632.5246 | -1.918771 | 632.5311 | 0.998 | 0.324747 |
| E G | 0.357976 | 0.6259427 | 2.493339 | 1.21367 | -2.135363 | 1.365577 | 0.118 | 0.2901595 |
| E H | 2.370629 | 1.142301 | 0.2352249 | 0.7483086 | 2.135404 | 1.365584 | 0.118 | 0.2901599 |
| F G | 1.27E-09 | 0.440624 | -0.4341099 | 631.6637 | 0.4341099 | 631.664 | 0.999 | 0.3247486 |
| G H | 0.2350218 | 0.5818533 | -0.0556911 | 0.5608296 | 0.290713 | 0.8068983 | 0.719 | 0.338798 |
| **Kidney transplantation in discontinuation** | | | | | | | | |
| A G | 0.2315115 | 0.1880823 | -0.0631848 | 111.7911 | 0.2946963 | 111.7912 | 0.998 | 2.00E-07 |
| B G | 0.4132469 | 0.4540185 | 0.7579147 | 1.692643 | -0.3446678 | 1.752477 | 0.844 | 3.27E-07 |
| B H | 1.098608 | 1.637501 | 0.7539243 | 0.6243796 | 0.3446833 | 1.752501 | 0.844 | 3.64E-08 |
| C H | -0.1470398 | 0.1381298 | 1.333081 | 447.5406 | -1.480121 | 447.5407 | 0.997 | 4.39E-06 |
| G H | 0.6709095 | 0.501284 | -0.31946 | 0.7433086 | 0.9903696 | 0.8965444 | 0.269 | 1.45E-08 |
| G I | -0.4054629 | 0.7287023 | 0.8238178 | 0.6192049 | -1.229281 | 0.9562535 | 0.199 | 2.85E-06 |
| H I | 0.1517591 | 0.3901895 | -1.077529 | 0.8730265 | 1.229288 | 0.9562546 | 0.199 | 3.51E-06 |
| **AEs** | | | | | | | | |
| A G | -0.1003247 | 0.1308197 | 0.0853923 | 0.4214007 | -0.1857169 | 0.4422579 | 0.675 | 0.1910957 |
| A H | -0.2371046 | 0.3718693 | -0.4228199 | 0.2394013 | 0.1857153 | 0.4422666 | 0.675 | 0.1910961 |
| B G | -0.1069314 | 0.182615 | 0.4090338 | 0.3341206 | -0.5159651 | 0.3546765 | 0.146 | 1.29E-01 |
| B H | -0.0072736 | 0.2704875 | -0.5232397 | 0.2570488 | 0.5159661 | 0.3546738 | 0.146 | 1.29E-01 |
| C H | 0.1641914 | 0.1965151 | -0.8901181 | 449.04 | 1.05431 | 449.0406 | 0.998 | 0.1674094 |
| D H | -0.2952293 | 0.4662727 | -0.4315734 | 630.7471 | 0.1363441 | 630.7475 | 1 | 0.1674091 |
| E G | 0.2972504 | 0.885525 | 0.1290993 | 0.6880513 | 0.1681511 | 1.121415 | 0.881 | 0.1709122 |
| E H | -0.1473253 | 0.6654367 | 0.0208218 | 0.9026408 | -0.1681471 | 1.121413 | 0.881 | 0.1709123 |
| F G | -0.0614228 | 0.2626421 | -0.1061504 | 641.1789 | 0.0447276 | 641.1791 | 1 | 0.1674091 |
| G H | -0.4664769 | 0.2791264 | -0.1715654 | 0.2145909 | -0.2949115 | 0.3497526 | 0.399 | 0.1714788 |
| G I | -0.5156745 | 0.2954406 | -0.0253331 | 0.3842475 | -0.4903414 | 0.484953 | 0.312 | 0.1552803 |
| H I | 0.1831224 | 0.3372539 | -0.3072185 | 0.3484813 | 0.4903409 | 0.484953 | 0.312 | 0.1552808 |
| **SAEs** | | | | | | | | |
| A G | -0.0444254 | 0.0850513 | -0.6434781 | 0.3529623 | 0.5990528 | 0.3624087 | 0.098 | 0.087577 |
| A H | -0.4313557 | 0.3185559 | 0.1677062 | 0.1728129 | -0.5990619 | 0.3624116 | 0.098 | 0.0875762 |
| B G | 0.0226941 | 0.1615107 | 0.2646428 | 0.2707787 | -0.2419487 | 0.3363228 | 0.472 | 8.74E-02 |
| B H | 0.2797655 | 0.20181 | 0.0378225 | 0.2918812 | 0.241943 | 0.3363183 | 0.472 | 8.74E-02 |
| C H | 0.128612 | 0.1249087 | -0.0870821 | 455.9122 | 0.2156941 | 455.9124 | 1 | 0.1216822 |
| D H | -0.7175386 | 0.7343031 | 0.7584831 | 631.1464 | -1.476022 | 631.1476 | 0.998 | 0.1216846 |
| E G | -0.5816346 | 0.4803387 | -0.4467533 | 0.4044802 | -0.1348813 | 0.6279567 | 0.83 | 0.1303369 |
| E H | -0.3454045 | 0.3744235 | -0.4802841 | 0.5041194 | 0.1348796 | 0.6279566 | 0.83 | 0.1303367 |
| F G | 3.17E-08 | 0.3884819 | -0.1757051 | 633.0702 | 0.1757051 | 633.0705 | 1 | 0.1216843 |
| G H | 0.7036442 | 0.3604256 | -0.0026584 | 0.1562124 | 0.7063026 | 0.3929712 | 0.072 | 0.1081031 |
| G I | -0.0902495 | 0.1994545 | 0.2729235 | 0.2627377 | -0.363173 | 0.3333849 | 0.276 | 0.0934126 |
| H I | 0.0788537 | 0.207319 | -0.2843191 | 0.2610602 | 0.3631729 | 0.3333671 | 0.276 | 0.0934123 |
| **MACE** | | | | | | | | |
| A G | 0.1353571 | 0.1441597 | -0.263852 | 0.781016 | 0.399209 | 0.7963912 | 0.616 | 0.1270871 |
| A H | -0.2436221 | 0.692679 | 0.155595 | 0.393016 | -0.3992171 | 0.796408 | 0.616 | 0.127086 |
| B G | 0.0251635 | 0.1524197 | 0.4243726 | 0.7794545 | -0.3992091 | 0.7964037 | 0.616 | 0.1270867 |
| B H | 0.0454032 | 0.3270711 | -0.353808 | 0.7261383 | 0.3992112 | 0.7963995 | 0.616 | 0.1270867 |
| C H | 0.0348299 | 0.1326191 | 0.1089233 | 363.57 | -0.0740933 | 363.5698 | 1 | 0.1097049 |
| E H | 0.3011051 | 0.9303485 | -0.1858989 | 633.0048 | 0.487004 | 633.0068 | 0.999 | 0.1097029 |
| **All-cause mortality** | | | | | | | | |
| A G | -0.0916433 | 0.1032927 | -1.761271 | 1.566857 | 1.669628 | 1.570258 | 0.288 | 5.85E-08 |
| A H | -1.636018 | 1.553477 | 0.0343249 | 0.2311986 | -1.670342 | 1.570587 | 0.288 | 5.69E-08 |
| B G | -0.0426824 | 0.0971923 | -0.566486 | 0.4865941 | 0.5238036 | 0.4962058 | 0.291 | 1.49E-08 |
| B H | -0.3550502 | 0.4277624 | 0.1687559 | 0.2514771 | -0.5238061 | 0.496207 | 0.291 | 4.49E-08 |
| C H | 0.0675908 | 0.0886585 | -0.0146811 | 388.6271 | 0.0822719 | 388.6271 | 1 | 0.0000394 |
| D H | 0.0114944 | 2.005739 | -0.0152603 | 632.2459 | 0.0267547 | 632.2554 | 1 | 2.05E-06 |
| E G | -1.025489 | 1.119692 | 0.6471354 | 1.030997 | -1.672625 | 1.522062 | 0.272 | 2.79E-08 |
| E H | 0.7132152 | 1.010017 | -0.959406 | 1.138652 | 1.672621 | 1.52206 | 0.272 | 9.44E-07 |
| F G | 0.5753641 | 0.6351035 | -0.7730033 | 633.8681 | 1.348367 | 633.869 | 0.998 | 3.04E-06 |
| G H | 0.2711205 | 0.2661996 | -0.1569989 | 0.3215088 | 0.4281193 | 0.4174087 | 0.305 | 5.55E-10 |
| G I | -0.010701 | 0.3525993 | 0.1112435 | 0.4484801 | -0.1219445 | 0.5704914 | 0.831 | 2.58E-07 |
| H I | -0.0043384 | 0.3892736 | -0.1262827 | 0.4170455 | 0.1219443 | 0.5704918 | 0.831 | 6.55E-06 |

Abbreviations: A=Roxadustat; B=Daprodustatt; C=Vadadustate; D=Enarodustat; E=Molidustat; F=Desidustat; G=rhEPO; H=DPO; I=MPG-rhEPO.

**Table S4: Meta-regressions and sensitivity analyses**

| **Hb response** | | | | | | | | | | | | |
| --- | --- | --- | --- | --- | --- | --- | --- | --- | --- | --- | --- | --- |
| Meta-regression Number of obs = 19 | | | | | | | | | | | | |
| REML estimate of between-study variance tau2 = .036 | | | | | | | | | | | | |
| % residual variation due to heterogeneity I-squared_res = 41.95% | | | | | | | | | | | | |
| Proportion of between-study variance explained Adj R-squared = 79.11% | | | | | | | | | | | | |
| Joint test for all covariates Model F(8,10) = 4.00 | | | | | | | | | | | | |
| With Knapp-Hartung modification Prob > F = 0.0221 | | | | | | | | | | | | |
| _y | Coef. | Std. Err. | t | P>t | [95% Conf.Interval] | | | | | | | |
| Duration | -0.1002123 | 0.2734459 | -0.37 | 0.722 | -0.7094877 | | | | 0.509063 | | | |
| **Mean_age** | 1.450901 | 0.3407323 | 4.26 | 0.002 | 0.6917022 | | | | 2.2101 | | | |
| Hb_baseline | -0.3179474 | 0.3113946 | -1.02 | 0.331 | -1.011778 | | | | 0.3758831 | | | |
| Dialysis_method | 0.2049621 | 0.3552243 | 0.58 | 0.577 | -0.5865268 | | | | 0.9964511 | | | |
| Sample_size | 0.0026335 | 0.2557932 | 0.01 | 0.992 | -0.5673092 | | | | 0.5725762 | | | |
| Fellow_up | 0.0372593 | 0.1997577 | 0.19 | 0.856 | -0.4078287 | | | | 0.4823472 | | | |
| Target_hemoglobin_levels | 0.0715853 | 0.2179788 | 0.33 | 0.749 | -0.4141017 | | | | 0.5572724 | | | |
| Race | -0.4044848 | 0.2436537 | -1.66 | 0.128 | -0.947379 | | | | 0.1384094 | | | |
| _cons | -1.060297 | 0.8550086 | -1.24 | 0.243 | -2.965375 | | | | 0.8447809 | | | |
| **RBC transfusion** | | | | | | | | | | | | |
| Meta-regression Number of obs = 15 | | | | | | | | | | | | |
| REML estimate of between-study variance tau2 = .09829 | | | | | | | | | | | | |
| % residual variation due to heterogeneity I-squared_res = 36.76% | | | | | | | | | | | | |
| Proportion of between-study variance explained Adj R-squared = -302.20% | | | | | | | | | | | | |
| Joint test for all covariates Model F(8,6) = 0.18 | | | | | | | | | | | | |
| With Knapp-Hartung modification Prob > F = 0.9860 | | | | | | | | | | | | |
| _y | Coef. | Std. Err. | t | P>t | [95% Conf.Interval] | | | | | | | |
| Duration | 0.0664989 | 0.9126755 | 0.07 | 0.944 | -2.166738 | | | | 2.299735 | | | |
| Mean_age | 0.1984772 | 0.6675672 | 0.3 | 0.776 | -1.435001 | | | | 1.831955 | | | |
| Hb_baseline | -0.1424837 | 0.6519194 | -0.22 | 0.834 | -1.737673 | | | | 1.452706 | | | |
| Dialysis_method | -0.1301748 | 0.7319416 | -0.18 | 0.865 | -1.921171 | | | | 1.660822 | | | |
| Sample_size | -0.1050272 | 0.383867 | -0.27 | 0.794 | -1.044316 | | | | 0.8342615 | | | |
| Fellow_up | 0.0744888 | 0.3733033 | 0.2 | 0.848 | -0.8389514 | | | | 0.9879289 | | | |
| Target_hemoglobin_levels | -0.2684129 | 0.4243306 | -0.63 | 0.550 | -1.306712 | | | | 0.7698868 | | | |
| Race | -0.5436928 | 0.7884343 | -0.69 | 0.516 | -2.472922 | | | | 1.385536 | | | |
| _cons | 1.223158 | 1.953359 | 0.63 | 0.554 | -3.556538 | | | | 6.002854 | | | |
| **Discontinuation** | | | | | | | | | | | | |
| Meta-regression Number of obs = 24 | | | | | | | | | | | | |
| REML estimate of between-study variance tau2 = .05399 | | | | | | | | | | | | |
| % residual variation due to heterogeneity I-squared_res = 50.57% | | | | | | | | | | | | |
| Proportion of between-study variance explained Adj R-squared = -13.03% | | | | | | | | | | | | |
| Joint test for all covariates Model F(8,15) = 1.05 | | | | | | | | | | | | |
| With Knapp-Hartung modification Prob > F = 0.4436 | | | | | | | | | | | | |
| _y | Coef. | Std. Err. | t | P>t | [95% Conf.Interval] | | | | | | | |
| Duration | -0.2542572 | 0.2865442 | -0.89 | 0.389 | -0.8650117 | | | | 0.3564973 | | | |
| Mean_age | -0.365933 | 0.2754367 | -1.33 | 0.204 | -0.9530125 | | | | 0.2211464 | | | |
| Hb_baseline | 0.455888 | 0.2935584 | 1.55 | 0.141 | -0.1698169 | | | | 1.081593 | | | |
| Dialysis_method | 0.5023993 | 0.3082318 | 1.63 | 0.124 | -0.1545813 | | | | 1.15938 | | | |
| Sample_size | 0.0684142 | 0.19642 | 0.35 | 0.732 | -0.3502451 | | | | 0.4870735 | | | |
| Fellow_up | -0.3730135 | 0.1890323 | -1.97 | 0.067 | -0.7759262 | | | | 0.0298992 | | | |
| Target_hemoglobin_levels | -0.2449344 | 0.2012196 | -1.22 | 0.242 | -0.6738239 | | | | 0.1839551 | | | |
| Race | 0.2173783 | 0.2545569 | 0.85 | 0.407 | -0.3251969 | | | | 0.7599534 | | | |
| _cons | -0.4434209 | 0.8597317 | -0.52 | 0.614 | -2.275896 | | | | 1.389054 | | | |
| **AEs in discontinuation** | | | | | | | | | | | | |
| Meta-regression Number of obs = 20 | | | | | | | | | | | | |
| REML estimate of between-study variance tau2 = .1049 | | | | | | | | | | | | |
| % residual variation due to heterogeneity I-squared_res = 47.44% | | | | | | | | | | | | |
| Proportion of between-study variance explained Adj R-squared = -53.39% | | | | | | | | | | | | |
| Joint test for all covariates Model F(8,11) = 0.91 | | | | | | | | | | | | |
| With Knapp-Hartung modification Prob > F = 0.5396 | | | | | | | | | | | | |
| _y | Coef. | Std. Err. | t | P>t | [95% Conf.Interval] | | | | | | | |
| Duration | -1.041843 | 0.5338354 | -1.95 | 0.077 | -2.216807 | | | | 0.1331205 | | | |
| Mean_age | -0.1333728 | 0.642062 | -0.21 | 0.839 | -1.546542 | | | | 1.279796 | | | |
| Hb_baseline | 1.038864 | 0.6274923 | 1.66 | 0.126 | -0.342237 | | | | 2.419965 | | | |
| Dialysis_method | 0.7646075 | 0.7226515 | 1.06 | 0.313 | -0.8259377 | | | | 2.355153 | | | |
| Sample_size | 0.2611104 | 0.3425228 | 0.76 | 0.462 | -0.4927772 | | | | 1.014998 | | | |
| Fellow_up | -0.3176234 | 0.3018767 | -1.05 | 0.315 | -0.9820496 | | | | 0.3468029 | | | |
| Target_hemoglobin_levels | -0.0033475 | 0.3623099 | -0.01 | 0.993 | -0.8007861 | | | | 0.7940912 | | | |
| Race | -0.4860984 | 0.6597199 | -0.74 | 0.477 | -1.938132 | | | | 0.9659354 | | | |
| _cons | -0.3621012 | 1.835058 | -0.2 | 0.847 | -4.401037 | | | | 3.676835 | | | |
| **Death in discontinuation** | | | | | | | | | | | | |
| Meta-regression Number of obs = 13 | | | | | | | | | | | | |
| REML estimate of between-study variance tau2 = 0 | | | | | | | | | | | | |
| % residual variation due to heterogeneity I-squared_res = 2.90% | | | | | | | | | | | | |
| Proportion of between-study variance explained Adj R-squared = 100.00% | | | | | | | | | | | | |
| Joint test for all covariates Model F(8,4) = 0.66 | | | | | | | | | | | | |
| With Knapp-Hartung modification Prob > F = 0.7157 | | | | | | | | | | | | |
| _y | Coef. | Std. Err. | t | P>t | | [95% Conf.Interval] | | | | | | |
| Duration | 0.5509656 | 1.210888 | 0.46 | 0.673 | | -2.810997 | | | | | 3.912928 | |
| Mean_age | 0.2480127 | 0.7751721 | 0.32 | 0.765 | | -1.90421 | | | | | 2.400236 | |
| Hb_baseline | -1.154182 | 0.802786 | -1.44 | 0.224 | | -3.383073 | | | | | 1.07471 | |
| Dialysis_method | -0.9404835 | 0.8223301 | -1.14 | 0.317 | | -3.223638 | | | | | 1.342671 | |
| Sample_size | 1.313555 | 1.147358 | 1.14 | 0.316 | | -1.872022 | | | | | 4.499131 | |
| Fellow_up | 0.174063 | 0.1806761 | 0.96 | 0.390 | | -0.3275744 | | | | | 0.6757004 | |
| Target_hemoglobin_levels | 1.348518 | 1.123043 | 1.2 | 0.296 | | -1.76955 | | | | | 4.466585 | |
| Race | 1.353735 | 1.140347 | 1.19 | 0.301 | | -1.812376 | | | | | 4.519845 | |
| _cons | -3.763629 | 4.249155 | -0.89 | 0.426 | | -15.56118 | | | | | 8.033918 | |
| **Refusal of treatment in discontinuation** | | | | | | | | | | | | |
| Meta-regression Number of obs = 14 | | | | | | | | | | | | |
| REML estimate of between-study variance tau2 = .1191 | | | | | | | | | | | | |
| % residual variation due to heterogeneity I-squared_res = 63.39% | | | | | | | | | | | | |
| Proportion of between-study variance explained Adj R-squared = -11.98% | | | | | | | | | | | | |
| Joint test for all covariates Model F(8,5) = 0.50 | | | | | | | | | | | | |
| With Knapp-Hartung modification Prob > F = 0.8155 | | | | | | | | | | | | |
| _y | Coef. | Std. Err. | t | P>t | | [95% Conf.Interval] | | | | | | |
| Duration | -0.0352796 | 0.6695763 | -0.05 | 0.960 | | -1.75648 | | | 1.685921 | | | |
| Mean_age | 0.2340014 | 0.7116286 | 0.33 | 0.756 | | -1.595298 | | | 2.063301 | | | |
| Hb_baseline | 0.4498116 | 0.6879524 | 0.65 | 0.542 | | -1.318626 | | | 2.21825 | | | |
| Dialysis_method | 0.079484 | 0.9425719 | 0.08 | 0.936 | | -2.343474 | | | 2.502442 | | | |
| Sample_size | 0.2816607 | 0.5906949 | 0.48 | 0.654 | | -1.236769 | | | 1.80009 | | | |
| Fellow_up | -0.4127888 | 0.4092095 | -1.01 | 0.359 | | -1.464695 | | | 0.6391176 | | | |
| Target_hemoglobin_levels | 0.1289135 | 0.5901872 | 0.22 | 0.836 | | -1.388211 | | | 1.646038 | | | |
| Race | -0.0080208 | 0.6168867 | -0.01 | 0.990 | | -1.593779 | | | 1.577737 | | | |
| _cons | -1.295067 | 2.109101 | -0.61 | 0.566 | | -6.716684 | | | 4.126551 | | | |
| **Content withdrawn in discontinuation** | | | | | | | | | | | | |
| Meta-regression Number of obs = 19 | | | | | | | | | | | | |
| REML estimate of between-study variance tau2 = .06117 | | | | | | | | | | | | |
| % residual variation due to heterogeneity I-squared_res = 23.55% | | | | | | | | | | | | |
| Proportion of between-study variance explained Adj R-squared = 59.30% | | | | | | | | | | | | |
| Joint test for all covariates Model F(8,10) = 1.71 | | | | | | | | | | | | |
| With Knapp-Hartung modification Prob > F = 0.2099 | | | | | | | | | | | | |
| _y | Coef. | Std. Err. | t | P>t | | [95% Conf.Interval] | | | | | | |
| Duration | 1.211869 | 0.6867551 | 1.76 | 0.108 | | -0.318317 | | | | 2.742055 | | |
| Mean_age | 0.0121117 | 0.5117756 | 0.02 | 0.982 | | -1.128195 | | | | 1.152419 | | |
| Hb_baseline | -0.3892878 | 0.5532337 | -0.7 | 0.498 | | -1.621969 | | | | 0.8433937 | | |
| Dialysis_method | -1.180185 | 0.7580103 | -1.56 | 0.151 | | -2.869137 | | | | 0.5087673 | | |
| Sample_size | 0.8006213 | 0.4608804 | 1.74 | 0.113 | | -0.2262843 | | | | 1.827527 | | |
| Fellow_up | -0.4545597 | 0.324249 | -1.4 | 0.191 | | -1.177031 | | | | 0.267912 | | |
| Target_hemoglobin_levels | 0.3687023 | 0.3998903 | 0.92 | 0.378 | | -0.5223088 | | | | 1.259713 | | |
| Race | 1.024911 | 0.5382489 | 1.9 | 0.086 | | -0.174382 | | | | 2.224205 | | |
| _cons | -2.394336 | 1.551392 | -1.54 | 0.154 | | -5.851051 | | | | 1.06238 | | |
| **Kidney transplantation in discontinuation** | | | | | | | | | | | | |
| Meta-regression Number of obs = 11 | | | | | | | | | | | | |
| REML estimate of between-study variance tau2 = .06016 | | | | | | | | | | | | |
| % residual variation due to heterogeneity I-squared_res = 44.50% | | | | | | | | | | | | |
| Proportion of between-study variance explained Adj R-squared = -74.87% | | | | | | | | | | | | |
| Joint test for all covariates Model F(8,2) = 0.46 | | | | | | | | | | | | |
| With Knapp-Hartung modification Prob > F = 0.8228 | | | | | | | | | | | | |
| _y | Coef. | Std. Err. | t | P>t | | | | [95% Conf.Interval] | | | | |
| Duration | 0.2611937 | 2.041531 | 0.13 | 0.910 | | | | -8.522805 | | 9.045192 | | |
| Mean_age | 0.4919726 | 1.231515 | 0.4 | 0.728 | | | | -4.80681 | | 5.790755 | | |
| Hb_baseline | -0.7746456 | 0.978064 | -0.79 | 0.511 | | | | -4.982915 | | 3.433624 | | |
| Dialysis_method | -0.0652514 | 1.451825 | -0.04 | 0.968 | | | | -6.311952 | | 6.181449 | | |
| Sample_size | -0.1232515 | 0.4725303 | -0.26 | 0.819 | | | | -2.156385 | | 1.909882 | | |
| Fellow_up | -1.222472 | 1.257882 | -0.97 | 0.434 | | | | -6.634702 | | 4.189757 | | |
| Target_hemoglobin_levels | -1.002417 | 1.187129 | -0.84 | 0.487 | | | | -6.110219 | | 4.105385 | | |
| Race | -0.0555638 | 1.187129 | -0.05 | 0.967 | | | | -5.163366 | | 5.052238 | | |
| _cons | 3.666091 | 5.424785 | 0.68 | 0.569 | | | | -19.67487 | | 27.00706 | | |
| **AEs** | | | | | | | | | | | | |
| Meta-regression Number of obs = 23 | | | | | | | | | | | | |
| REML estimate of between-study variance tau2 = 0.004036 | | | | | | | | | | | | |
| % residual variation due to heterogeneity I-squared_res = 11.78% | | | | | | | | | | | | |
| Proportion of between-study variance explained Adj R-squared = 61.78% | | | | | | | | | | | | |
| Joint test for all covariates Model F(8,16) = 1.63 | | | | | | | | | | | | |
| With Knapp-Hartung modification Prob > F = 0.2037 | | | | | | | | | | | | |
| _y | Coef. | Std. Err. | t | P>t | | | | [95% Conf.Interval] | | | | |
| Duration | 0.2998221 | 0.2555437 | 1.17 | 0.26 | | | | -0.2482646 | | 0.8479089 | | |
| Mean_age | -0.1694151 | 0.2751866 | -0.62 | 0.548 | | | | -0.7596316 | | 0.4208014 | | |
| Hb_baseline | -0.4864149 | 0.2908298 | -1.67 | 0.117 | | | | -1.110183 | | 0.137353 | | |
| Dialysis_method | -1.054833 | 0.3094032 | -3.41 | 0.004 | | | | -1.718436 | | -0.3912286 | | |
| Sample_size | 0.1957182 | 0.213083 | 0.92 | 0.374 | | | | -0.2612994 | | 0.6527357 | | |
| Fellow_up | 0.1687432 | 0.1272034 | 1.33 | 0.206 | | | | -0.1040811 | | 0.4415674 | | |
| Target_hemoglobin_levels | 0.0222078 | 0.1506559 | 0.15 | 0.885 | | | | -0.3009169 | | 0.3453325 | | |
| Race | -0.0859038 | 0.2156479 | -0.4 | 0.696 | | | | -0.5484227 | | 0.376615 | | |
| _cons | 1.520716 | 0.8201361 | 1.85 | 0.085 | | | | -0.2383015 | | 3.279733 | | |
| **SAEs** | | | | | | | | | | | | |
| Meta-regression Number of obs = 22 | | | | | | | | | | | | |
| REML estimate of between-study variance tau2 = 0.0001 | | | | | | | | | | | | |
| % residual variation due to heterogeneity I-squared_res = 26.96% | | | | | | | | | | | | |
| Proportion of between-study variance explained Adj R-squared = 100% | | | | | | | | | | | | |
| Joint test for all covariates Model F(8,13) = 0.67 | | | | | | | | | | | | |
| With Knapp-Hartung modification Prob > F = 0.7101 | | | | | | | | | | | | |
| _y | Coef. | Std. Err. | t | P>t | | | [95% Conf.Interval] | | | | | |
| Duration | 0.1617646 | 0.280203 | 0.58 | 0.574 | | | -0.4435772 | | | | | 0.7671064 |
| Mean_age | -0.1264151 | 0.2705415 | -0.47 | 0.648 | | | -0.7108844 | | | | | 0.4580542 |
| Hb_baseline | -0.0624249 | 0.2526584 | -0.25 | 0.809 | | | -0.6082603 | | | | | 0.4834104 |
| Dialysis_method | -0.2921349 | 0.2832445 | -1.03 | 0.321 | | | -0.9040475 | | | | | 0.3197777 |
| Sample_size | -0.0162259 | 0.2182796 | -0.07 | 0.942 | | | -0.4877904 | | | | | 0.4553386 |
| Fellow_up | 0.1477655 | 0.0902116 | 1.64 | 0.125 | | | -0.0471248 | | | | | 0.3426558 |
| Target_hemoglobin_levels | -0.0943405 | 0.1338724 | -0.7 | 0.493 | | | -0.3835542 | | | | | 0.1948733 |
| Race | -0.1568381 | 0.2323198 | -0.68 | 0.511 | | | -0.6587345 | | | | | 0.3450584 |
| _cons | 0.1617646 | 0.280203 | 0.58 | 0.574 | | | -0.4435772 | | | | | 0.7671064 |
| Duration | -0.1264151 | 0.2705415 | -0.47 | 0.648 | | | -0.7108844 | | | | | 0.4580542 |
| Mean_age | 0.5636684 | 0.8313027 | 0.68 | 0.51 | | | -1.232252 | | | | | 2.359589 |
| **MACE** | | | | | | | | | | | | |
| Meta-regression Number of obs = 13 | | | | | | | | | | | | |
| REML estimate of between-study variance tau2 = 0 | | | | | | | | | | | | |
| % residual variation due to heterogeneity I-squared_res = 0.00% | | | | | | | | | | | | |
| Proportion of between-study variance explained Adj R-squared = .% | | | | | | | | | | | | |
| Joint test for all covariates Model F(8,4) = 0.79 | | | | | | | | | | | | |
| With Knapp-Hartung modification Prob > F = 0.6399 | | | | | | | | | | | | |
| _y | Coef. | Std. Err. | t | P>t | [95% Conf.Interval] | | | | | | | |
| Duration | 0.4217164 | 0.7724521 | 0.55 | 0.614 | -1.722954 | | | | 2.566387 | | | |
| Mean_age | 1.753928 | 1.243026 | 1.41 | 0.231 | -1.697264 | | | | 5.20512 | | | |
| Hb_baseline | 0.6900379 | 1.691714 | 0.41 | 0.704 | -4.006914 | | | | 5.38699 | | | |
| Dialysis_method | 0.9806026 | 1.677676 | 0.58 | 0.59 | -3.677373 | | | | 5.638579 | | | |
| Sample_size | -0.0754659 | 0.2277564 | -0.33 | 0.757 | -0.707819 | | | | 0.5568872 | | | |
| Fellow_up | -0.0434231 | 0.113988 | -0.38 | 0.723 | -0.3599045 | | | | 0.2730582 | | | |
| Target_hemoglobin_levels | 0.3740163 | 0.2004715 | 1.87 | 0.136 | -0.1825818 | | | | 0.9306145 | | | |
| Race | -1.362725 | 1.145533 | -1.19 | 0.3 | -4.543233 | | | | 1.817784 | | | |
| _cons | -4.020128 | 4.470112 | -0.9 | 0.419 | -16.43115 | | | | 8.390892 | | | |
| **All-cause mortality** | | | | | | | | | | | | |
| Meta-regression Number of obs = 23 | | | | | | | | | | | | |
| REML estimate of between-study variance tau2 = 0 | | | | | | | | | | | | |
| % residual variation due to heterogeneity I-squared_res = 0.00% | | | | | | | | | | | | |
| Proportion of between-study variance explained Adj R-squared = .% | | | | | | | | | | | | |
| Joint test for all covariates Model F(9,13) = 0.72 | | | | | | | | | | | | |
| With Knapp-Hartung modification Prob > F = 0.6836 | | | | | | | | | | | | |
| _y | Coef. | Std. Err. | t | P>t | [95% Conf.Interval] | | | | | | | |
| Duration | 0.1136953 | 0.4743311 | 0.24 | 0.814 | -0.9110347 | | | | 1.138425 | | | |
| Mean_age | 0.0158746 | 0.4643986 | 0.03 | 0.973 | -0.9873975 | | | | 1.019147 | | | |
| Hb_baseline | 0.2377829 | 0.4258635 | 0.56 | 0.586 | -0.6822393 | | | | 1.157805 | | | |
| Dialysis_method | 0.2848435 | 0.4605435 | 0.62 | 0.547 | -0.7101002 | | | | 1.279787 | | | |
| Sample_size | 0.0575946 | 0.2369594 | 0.24 | 0.812 | -0.454325 | | | | 0.5695143 | | | |
| Fellow_up | 0.3532082 | 0.2277149 | 1.55 | 0.145 | -0.13874 | | | | 0.8451565 | | | |
| Target_hemoglobin_levels | -0.0459881 | 0.3680804 | -0.12 | 0.902 | -0.8411775 | | | | 0.7492013 | | | |
| Race | 0.5741074 | 0.5697142 | 1.01 | 0.332 | -0.6566853 | | | | 1.8049 | | | |
| _cons | -1.931522 | 1.519357 | -1.27 | 0.226 | -5.213894 | | | | 1.35085 | | | |

**Table S5: Risk of bias assessment in individual trials**

| **Study ID** | | **Treatment** | **Comparison** | **Random sequence generation** | **Allocation concealment** | **Blinding of participants and personnel** | **Blinding of outcome assessment** | **Incomplete outcome data** | **Selective reporting** | **Other bias** |
| --- | --- | --- | --- | --- | --- | --- | --- | --- | --- | --- |
| 1 | Provenzano 2016 | Roxadustat | rhEPO | Unclear Risk | Unclear Risk | Unclear Risk | Unclear Risk | Unclear Risk | Low Risk | Low Risk |
| 2 | Chen 2019 |  |  | Unclear Risk | Unclear Risk | Low Risk | Unclear Risk | Unclear Risk | Low Risk | Low Risk |
| 3 | CRisky 2021(a) |  |  | Low Risk | Low Risk | Unclear Risk | Unclear Risk | Unclear Risk | Low Risk | Low Risk |
| 4 | Provenzano 2021 |  |  | Low Risk | Low Risk | Unclear Risk | Unclear Risk | Low Risk | Unclear Risk | Low Risk |
| 5 | Charytan 2021 |  |  | Low Risk | Unclear Risk | Unclear Risk | Unclear Risk | Unclear Risk | Unclear Risk | Low Risk |
| 6 | Fishbane S 2022 |  |  | Low Risk | Unclear RIsk | Unclear RIsk | Unclear RIsk | Unclear RIsk | Unclear RIsk | Low Risk |
| 7 | Akizawa 2020 |  | DPO | Low Risk | Low Risk | Low Risk | Low Risk | Low Risk | Low Risk | Low Risk |
| 8 | CRisky 2021(b) |  |  | Low Risk | Low Risk | Unclear Risk | Unclear Risk | Unclear Risk | Low Risk | Low Risk |
| 9 | Meadowcroft2019 | Daprodustat | rhEPO | Low Risk | Low Risk | Low Risk | Low Risk | Low Risk | Low Risk | Low Risk |
| 10 | Singh AK 2021 |  |  | Low Risk | Low Risk | Low Risk | Low Risk | Low Risk | Low Risk | Unclear Risk |
| 11 | Coyne DW2022 |  |  | Low Risk | Low Risk | Low Risk | Low Risk | Low Risk | Low Risk | Low Risk |
| 12 | Akizawa T 2020 |  | DPO | Low Risk | Low Risk | Low Risk | Low Risk | Low Risk | Low Risk | Low Risk |
| 13 | Singh AK 2022 |  |  | Low Risk | Low Risk | Unclear RIsk | Low Risk | Low Risk | Low Risk | Low Risk |
| 14 | Nangaku2021 | Vadadustat | DPO | Unclear Risk | Unclear Risk | Low Risk | Unclear Risk | Low Risk | Low Risk | Low Risk |
| 15 | Eckardt 2021(a) |  |  | Low Risk | Unclear Risk | Unclear Risk | High Risk | Unclear Risk | Low Risk | High Risk |
| 16 | Eckardt 2021(b) |  |  | Low Risk | Unclear Risk | Unclear Risk | High Risk | Unclear Risk | Low Risk | Low Risk |
| 17 | Akizawa 2021 | Enarodustat | DPO | Low Risk | Low Risk | Low Risk | Low Risk | Low Risk | Low Risk | Low Risk |
| 18 | Akizawa 2019 | Molidustat | rhEPO | High Risk | High Risk | Unclear Risk | Unclear Risk | Low Risk | Low Risk | Low Risk |
| 19 | Akizawa 2021 |  | DPO | Low Risk | Low Risk | Low Risk | Low Risk | Low Risk | Low Risk | Low Risk |
| 20 | Gang2022 | Desidustat | rhEPO | Low Risk | Low Risk | Unclear RIsk | Low Risk | Low Risk | Low Risk | Low Risk |
| 21 | Vanrenterghem 2002 | rhEPO | DPO | Low Risk | Unclear Risk | Unclear Risk | Unclear Risk | Low Risk | Unclear Risk | Low Risk |
| 22 | Carrera 2010 |  |  | Low Risk | Low Risk | Unclear Risk | Unclear Risk | Low Risk | Low Risk | Low Risk |
| 23 | Bernieh 2014 |  |  | Unclear Risk | Unclear Risk | Unclear Risk | Unclear Risk | Low Risk | Unclear Risk | Low Risk |
| 24 | Sinha 2019 |  |  | Unclear Risk | Unclear Risk | Low Risk | Low Risk | Unclear Risk | Low Risk | Low Risk |
| 25 | Kinger M(2007) | MPG-EPO | rhEPO | Low Risk | Unclear Risk | Unclear Risk | Unclear Risk | Low Risk | Low Risk | Low Risk |
| 26 | LevinNW(2007) |  |  | Low Risk | Low Risk | Low Risk | Low Risk | Low Risk | Low Risk | Low Risk |
| 27 | Allen 2002 |  |  | Low Risk | Unclear Risk | Low Risk | Low Risk | Unclear Risk | Unclear Risk | Low Risk |

**Table S6: Summary of confidence in the evidence using the GRADE process**

| **Comparison** | **No. of head-to-head comparisons** | | **Across studies bias** | **Indirectness** | **Imprecision** | **Inconsisitency** | **Publication bias** | **Confidence rating** |
| --- | --- | --- | --- | --- | --- | --- | --- | --- |
| **Hb response** | | | | | | | | |
| Roxadustat VS rhEPO | 6 | | Downgrade | Downgrade | No downgrade | No downgrade | No downgrade | Low |
| Roxadustat VS DPO | 2 | | Downgrade | Downgrade | No downgrade | No downgrade | No downgrade | Low |
| DaprodustatVS rhEPO | 3 | | Downgrade | Downgrade | No downgrade | No downgrade | No downgrade | Low |
| DaprodustatVS DPO | 2 | | Downgrade | Downgrade | No downgrade | No downgrade | No downgrade | Low |
| Vadadustat VS DPO | 3 | | Downgrade | Downgrade | No downgrade | No downgrade | No downgrade | Low |
| Enarodustat VS DPO | 1 | | No downgrade | Downgrade | No downgrade | No downgrade | No downgrade | Moderate |
| Molidustat VS rhEPO | 1 | | No downgrade | Downgrade | No downgrade | No downgrade | No downgrade | Moderate |
| Molidustat VS DPO | 1 | | Downgrade | Downgrade | No downgrade | No downgrade | No downgrade | Low |
| Desidustat VS rhEPO | 1 | | Downgrade | Downgrade | No downgrade | No downgrade | No downgrade | Low |
| rhEPO VS DPO | 3 | | Downgrade | Downgrade | No downgrade | No downgrade | No downgrade | Low |
| rhEPO VS MPG-EPO | 2 | | Downgrade | Downgrade | No downgrade | No downgrade | No downgrade | Low |
| Roxadustat VS Daprodustat | - | | Downgrade | Downgrade | No downgrade | No downgrade | No downgrade | Very low |
| Roxadustat VS Vadadustat | - | | Downgrade | Downgrade | No downgrade | No downgrade | No downgrade | Very low |
| Roxadustat VS Enarodustat | - | | No downgrade | Downgrade | No downgrade | No downgrade | No downgrade | Low |
| Roxadustat VS Molidustat | - | | No downgrade | Downgrade | No downgrade | No downgrade | No downgrade | Low |
| Roxadustat VS Desidustat | - | | Downgrade | Downgrade | No downgrade | No downgrade | No downgrade | Very low |
| Roxadustat VS MPG-EPO | - | | Downgrade | Downgrade | No downgrade | No downgrade | No downgrade | Very low |
| DaprodustatVS Vadadustat | - | | Downgrade | Downgrade | No downgrade | No downgrade | No downgrade | Very low |
| DaprodustatVS Enarodustat | - | | No downgrade | Downgrade | No downgrade | No downgrade | No downgrade | Low |
| DaprodustatVS Molidustat | - | | No downgrade | Downgrade | No downgrade | No downgrade | No downgrade | Low |
| DaprodustatVS Desidustat | - | | Downgrade | Downgrade | No downgrade | No downgrade | No downgrade | Very low |
| DaprodustatVS MPG-EPO | - | | Downgrade | Downgrade | No downgrade | No downgrade | No downgrade | Very low |
| Vadadustat VS Enarodustat | - | | No downgrade | Downgrade | No downgrade | No downgrade | No downgrade | Low |
| Vadadustat VS Molidustat | - | | No downgrade | Downgrade | No downgrade | No downgrade | No downgrade | Low |
| Vadadustat VS Desidustat | - | | Downgrade | Downgrade | No downgrade | No downgrade | No downgrade | Very low |
| Vadadustat VS rhEPO | - | | Downgrade | Downgrade | No downgrade | No downgrade | No downgrade | Very low |
| Vadadustat VS MPG-EPO | - | | Downgrade | Downgrade | No downgrade | No downgrade | No downgrade | Very low |
| Enarodustat VS Molidustat | - | | No downgrade | Downgrade | No downgrade | No downgrade | No downgrade | Low |
| Enarodustat VS Desidustat | - | | No downgrade | Downgrade | No downgrade | No downgrade | No downgrade | Low |
| Enarodustat VS rhEPO | - | | No downgrade | Downgrade | No downgrade | No downgrade | No downgrade | Low |
| Enarodustat VS MPG-EPO | - | | No downgrade | Downgrade | No downgrade | No downgrade | No downgrade | Low |
| Molidustat VS Desidustat | - | | No downgrade | Downgrade | No downgrade | No downgrade | No downgrade | Low |
| Molidustat VS rhEPO | - | | No downgrade | Downgrade | No downgrade | No downgrade | No downgrade | Low |
| Molidustat VS MPG-EPO | - | | No downgrade | Downgrade | No downgrade | No downgrade | No downgrade | Low |
| Desidustat VS DPO | - | | Downgrade | Downgrade | No downgrade | No downgrade | No downgrade | Very low |
| Desidustat VS MPG-EPO | - | | Downgrade | Downgrade | No downgrade | No downgrade | No downgrade | Very low |
| **RBC transfusion** | | | | | | | | |
| Roxadustat VS rhEPO | 4 | | Downgrade | No downgrade | No Downgrade | Downgrade | No downgrade | Low |
| DaprodustatVS rhEPO | 2 | | No Downgrade | No downgrade | Downgrade | No downgrade | No downgrade | Moderate |
| DaprodustatVS DPO | 1 | | No Downgrade | No downgrade | Downgrade | No downgrade | No downgrade | Moderate |
| Vadadustat VS DPO | 3 | | Downgrade | No downgrade | Downgrade | No downgrade | No downgrade | Low |
| Molidustat VS DPO | 1 | | Downgrade | No downgrade | Downgrade | No downgrade | No downgrade | Low |
| rhEPO VS DPO | 1 | | Downgrade | No downgrade | Downgrade | No downgrade | No downgrade | Low |
| rhEPO VS MPG-EPO | 2 | | Downgrade | No downgrade | Downgrade | No downgrade | No downgrade | Low |
| DPO VS MPG-EPO | 1 | | Downgrade | No downgrade | Downgrade | No downgrade | No downgrade | Low |
| Roxadustat VS Daprodustat | - | | No Downgrade | No downgrade | Downgrade | No downgrade | No downgrade | Low |
| Roxadustat VS Vadadustat | - | | Downgrade | No downgrade | Downgrade | No downgrade | No downgrade | Very low |
| Roxadustat VS Molidustat | - | | Downgrade | No downgrade | Downgrade | No downgrade | No downgrade | Very low |
| Roxadustat VS DPO | - | | Downgrade | No downgrade | Downgrade | No downgrade | No downgrade | Very low |
| Roxadustat VS MPG-EPO | - | | Downgrade | No downgrade | Downgrade | No downgrade | No downgrade | Very low |
| DaprodustatVS Vadadustat | - | | No Downgrade | No downgrade | Downgrade | No downgrade | No downgrade | Low |
| DaprodustatVS Molidustat | - | | No Downgrade | No downgrade | Downgrade | No downgrade | No downgrade | Low |
| DaprodustatVS MPG-EPO | - | | No Downgrade | No downgrade | Downgrade | No downgrade | No downgrade | Low |
| Vadadustat VS Molidustat | - | | Downgrade | No downgrade | Downgrade | No downgrade | No downgrade | Very low |
| Vadadustat VS rhEPO | - | | Downgrade | No downgrade | Downgrade | No downgrade | No downgrade | Very low |
| Vadadustat VS MPG-EPO | - | | Downgrade | No downgrade | Downgrade | No downgrade | No downgrade | Very low |
| Molidustat VS rhEPO | - | | Downgrade | No downgrade | Downgrade | No downgrade | No downgrade | Very low |
| Molidustat VS MPG-EPO | - | | Downgrade | No downgrade | Downgrade | No downgrade | No downgrade | Very low |
| **Discontinuation** | | | | | | | | |
| Roxadustat VS rhEPO | 5 | | Downgrade | No downgrade | No Downgrade | Downgrade | No downgrade | Low |
| Roxadustat VS DPO | 1 | | Downgrade | No downgrade | No Downgrade | Downgrade | No downgrade | Low |
| DaprodustatVS rhEPO | 3 | | No Downgrade | No downgrade | Downgrade | No downgrade | No downgrade | Moderate |
| DaprodustatVS DPO | 2 | | Downgrade | No downgrade | Downgrade | No downgrade | No downgrade | Low |
| Vadadustat VS DPO | 3 | | Downgrade | No downgrade | No Downgrade | No downgrade | No downgrade | Moderate |
| Enarodustat VS DPO | 1 | | No Downgrade | No downgrade | Downgrade | No downgrade | No downgrade | Moderate |
| Molidustat VS rhEPO | 1 | | Downgrade | No downgrade | Downgrade | No downgrade | No downgrade | Low |
| Molidustat VS DPO | 1 | | Downgrade | No downgrade | Downgrade | No downgrade | No downgrade | Low |
| Desidustat VS rhEPO | 1 | | Downgrade | No downgrade | Downgrade | No downgrade | No downgrade | Low |
| rhEPO VS DPO | 3 | | Downgrade | No downgrade | Downgrade | No downgrade | No downgrade | Low |
| rhEPO VS MPG-EPO | 3 | | Downgrade | No downgrade | No Downgrade | Downgrade | No downgrade | Low |
| Roxadustat VS Daprodustat | - | | No Downgrade | No downgrade | Downgrade | No downgrade | No downgrade | Low |
| Roxadustat VS Vadadustat | - | | Downgrade | No downgrade | Downgrade | No downgrade | No downgrade | Very low |
| Roxadustat VS Enarodustat | - | | No Downgrade | No downgrade | Downgrade | No downgrade | No downgrade | Low |
| Roxadustat VS Molidustat | - | | Downgrade | No downgrade | Downgrade | No downgrade | No downgrade | Very low |
| Roxadustat VS Desidustat | - | | Downgrade | No downgrade | Downgrade | No downgrade | No downgrade | Very low |
| Roxadustat VS MPG-EPO | - | | Downgrade | No downgrade | No Downgrade | No downgrade | No downgrade | Low |
| DaprodustatVS Vadadustat | - | | Downgrade | No downgrade | Downgrade | No downgrade | No downgrade | Very low |
| DaprodustatVS Enarodustat | - | | No Downgrade | No downgrade | Downgrade | No downgrade | No downgrade | Low |
| DaprodustatVS Molidustat | - | | Downgrade | No downgrade | Downgrade | No downgrade | No downgrade | Very low |
| DaprodustatVS Desidustat | - | | Downgrade | No downgrade | Downgrade | No downgrade | No downgrade | Very low |
| DaprodustatVS MPG-EPO | - | | Downgrade | No downgrade | No Downgrade | No downgrade | No downgrade | Low |
| Vadadustat VS Enarodustat | - | | No Downgrade | No downgrade | Downgrade | No downgrade | No downgrade | Low |
| Vadadustat VS Molidustat | - | | Downgrade | No downgrade | Downgrade | No downgrade | No downgrade | Very low |
| Vadadustat VS Desidustat | - | | Downgrade | No downgrade | Downgrade | No downgrade | No downgrade | Very low |
| Vadadustat VS rhEPO | - | | Downgrade | No downgrade | No Downgrade | Downgrade | No downgrade | Very low |
| Vadadustat VS MPG-EPO | - | | Downgrade | No downgrade | No Downgrade | No downgrade | No downgrade | Low |
| Enarodustat VS Molidustat | - | | No Downgrade | No downgrade | Downgrade | No downgrade | No downgrade | Low |
| Enarodustat VS Desidustat | - | | No Downgrade | No downgrade | Downgrade | No downgrade | No downgrade | Low |
| Enarodustat VS rhEPO | - | | No Downgrade | No downgrade | Downgrade | No downgrade | No downgrade | Low |
| Enarodustat VS MPG-EPO | - | | No Downgrade | No downgrade | Downgrade | No downgrade | No downgrade | Low |
| Molidustat VS Desidustat | - | | Downgrade | No downgrade | Downgrade | No downgrade | No downgrade | Very low |
| Molidustat VS MPG-EPO | - | | Downgrade | No downgrade | No Downgrade | Downgrade | No downgrade | Very low |
| Desidustat VS DPO | - | | Downgrade | No downgrade | Downgrade | No downgrade | No downgrade | Very low |
| Desidustat VS MPG-EPO | - | | Downgrade | No downgrade | Downgrade | No downgrade | No downgrade | Very low |
| DPO VS MPG-EPO | - | | Downgrade | No downgrade | Downgrade | No downgrade | No downgrade | Very low |
| **AEs in discontinuation** | | | | | | | | |
| Roxadustat VS rhEPO | 4 | | Downgrade | No downgrade | No Downgrade | Downgrade | No downgrade | Low |
| Roxadustat VS DPO | 1 | | Downgrade | No downgrade | No Downgrade | Downgrade | No downgrade | Low |
| DaprodustatVS rhEPO | 3 | | No Downgrade | No downgrade | Downgrade | No downgrade | No downgrade | Moderate |
| DaprodustatVS DPO | 2 | | Downgrade | No downgrade | Downgrade | No downgrade | No downgrade | Low |
| Vadadustat VS DPO | 3 | | Downgrade | No downgrade | No Downgrade | No downgrade | No downgrade | Moderate |
| Enarodustat VS DPO | 1 | | No Downgrade | No downgrade | Downgrade | No downgrade | No downgrade | Moderate |
| Molidustat VS rhEPO | 1 | | Downgrade | No downgrade | No Downgrade | Downgrade | No downgrade | Low |
| Molidustat VS DPO | 1 | | Downgrade | No downgrade | No Downgrade | No downgrade | No downgrade | Moderate |
| rhEPO VS DPO | 1 | | Downgrade | No downgrade | Downgrade | No downgrade | No downgrade | Low |
| rhEPO VS MPG-EPO | 2 | | Downgrade | No downgrade | Downgrade | No downgrade | No downgrade | Low |
| DPO VS MPG-EPO | 1 | | Downgrade | No downgrade | Downgrade | No downgrade | No downgrade | Low |
| Roxadustat VS Daprodustat | - | | No Downgrade | No downgrade | Downgrade | No downgrade | No downgrade | Low |
| Roxadustat VS Vadadustat | - | | Downgrade | No downgrade | Downgrade | No downgrade | No downgrade | Very low |
| Roxadustat VS Enarodustat | - | | No Downgrade | No downgrade | Downgrade | No downgrade | No downgrade | Low |
| Roxadustat VS Molidustat | - | | Downgrade | No downgrade | Downgrade | No downgrade | No downgrade | Very low |
| Roxadustat VS MPG-EPO | - | | Downgrade | No downgrade | Downgrade | No downgrade | No downgrade | Very low |
| DaprodustatVS Vadadustat | - | | Downgrade | No downgrade | Downgrade | No downgrade | No downgrade | Very low |
| DaprodustatVS Enarodustat | - | | No Downgrade | No downgrade | Downgrade | No downgrade | No downgrade | Low |
| DaprodustatVS Molidustat | - | | No Downgrade | No downgrade | No Downgrade | Downgrade | No downgrade | Low |
| DaprodustatVS MPG-EPO | - | | Downgrade | No downgrade | Downgrade | No downgrade | No downgrade | Very low |
| Vadadustat VS Enarodustat | - | | No Downgrade | No downgrade | Downgrade | No downgrade | No downgrade | Low |
| Vadadustat VS Molidustat | - | | Downgrade | No downgrade | Downgrade | No downgrade | No downgrade | Very low |
| Vadadustat VS rhEPO | - | | Downgrade | No downgrade | Downgrade | No downgrade | No downgrade | Very low |
| Vadadustat VS MPG-EPO | - | | Downgrade | No downgrade | Downgrade | No downgrade | No downgrade | Very low |
| Enarodustat VS Molidustat | - | | No Downgrade | No downgrade | Downgrade | No downgrade | No downgrade | Low |
| Enarodustat VS rhEPO | - | | No Downgrade | No downgrade | Downgrade | No downgrade | No downgrade | Low |
| Enarodustat VS MPG-EPO | - | | No Downgrade | No downgrade | Downgrade | No downgrade | No downgrade | Low |
| Molidustat VS MPG-EPO | - | | Downgrade | No downgrade | No Downgrade | Downgrade | No downgrade | Very low |
| **Death in discontinuation** | | | | | | | | |
| Roxadustat VS rhEPO | 2 | | Downgrade | No downgrade | Downgrade | Downgrade | No downgrade | Very low |
| Roxadustat VS DPO | 1 | | Downgrade | No downgrade | Downgrade | Downgrade | No downgrade | Very low |
| DaprodustatVS rhEPO | 2 | | No downgrade | No downgrade | Downgrade | Downgrade | No downgrade | Low |
| Vadadustat VS DPO | 1 | | Downgrade | No downgrade | Downgrade | Downgrade | No downgrade | Very low |
| Molidustat VS rhEPO | 1 | | No downgrade | No downgrade | Downgrade | Downgrade | No downgrade | Low |
| Molidustat VS DPO | 1 | | No downgrade | No downgrade | Downgrade | Downgrade | No downgrade | Low |
| Desidustat VS rhEPO | 1 | | Downgrade | No downgrade | Downgrade | Downgrade | No downgrade | Very low |
| rhEPO VS DPO | 1 | | Downgrade | No downgrade | Downgrade | Downgrade | No downgrade | Very low |
| rhEPO VS MPG-EPO | 2 | | Downgrade | No downgrade | Downgrade | Downgrade | No downgrade | Very low |
| DPO VS MPG-EPO | 1 | | Downgrade | No downgrade | Downgrade | Downgrade | No downgrade | Very low |
| Roxadustat VS Daprodustat | - | | No downgrade | No downgrade | Downgrade | Downgrade | No downgrade | Very low |
| Roxadustat VS Vadadustat | - | | Downgrade | No downgrade | Downgrade | Downgrade | No downgrade | Very low |
| Roxadustat VS Molidustat | - | | Downgrade | No downgrade | Downgrade | Downgrade | No downgrade | Very low |
| Roxadustat VS Desidustat | - | | Downgrade | No downgrade | Downgrade | Downgrade | No downgrade | Very low |
| Roxadustat VS MPG-EPO | - | | Downgrade | No downgrade | Downgrade | Downgrade | No downgrade | Very low |
| DaprodustatVS Vadustate | - | | No downgrade | No downgrade | Downgrade | Downgrade | No downgrade | Very low |
| DaprodustatVS Molidustat | - | | No downgrade | No downgrade | Downgrade | Downgrade | No downgrade | Very low |
| DaprodustatVS Desidustat | - | | No downgrade | No downgrade | Downgrade | Downgrade | No downgrade | Very low |
| DaprodustatVS DPO | - | | No downgrade | No downgrade | Downgrade | Downgrade | No downgrade | Very low |
| DaprodustatVS MPG-EPO | - | | No downgrade | No downgrade | Downgrade | Downgrade | No downgrade | Very low |
| Vadadustat VS Molidustat | - | | Downgrade | No downgrade | Downgrade | Downgrade | No downgrade | Very low |
| Vadadustat VS Desidustat | - | | Downgrade | No downgrade | Downgrade | Downgrade | No downgrade | Very low |
| Vadadustat VS DPO | - | | Downgrade | No downgrade | Downgrade | Downgrade | No downgrade | Very low |
| Vadadustat VS MPG-EPO | - | | Downgrade | No downgrade | Downgrade | Downgrade | No downgrade | Very low |
| Molidustat VS Desidustat | - | | Downgrade | No downgrade | Downgrade | Downgrade | No downgrade | Very low |
| Molidustat VS MPG-EPO | - | | Downgrade | No downgrade | Downgrade | Downgrade | No downgrade | Very low |
| Desidustat VS DPO | - | | Downgrade | No downgrade | Downgrade | Downgrade | No downgrade | Very low |
| Desidustat VS MPG-EPO | - | | Downgrade | No downgrade | Downgrade | Downgrade | No downgrade | Very low |
| **Refusal of treatment in discontinuation** | | | | | | | | |
| Roxadustat VS rhEPO | 2 | | Downgrade | No downgrade | No downgrade | Downgrade | No downgrade | Low |
| Roxadustat VS DPO | 1 | | Downgrade | No downgrade | Downgrade | Downgrade | No downgrade | Very low |
| DaprodustatVS rhEPO | 3 | | No downgrade | No downgrade | Downgrade | Downgrade | No downgrade | Low |
| DaprodustatVS DPO | 1 | | No downgrade | No downgrade | Downgrade | Downgrade | No downgrade | Low |
| Vadadustat VS DPO | 2 | | Downgrade | No downgrade | No downgrade | Downgrade | No downgrade | Low |
| Molidustat VS rhEPO | 1 | | Downgrade | No downgrade | Downgrade | Downgrade | No downgrade | Very low |
| Molidustat VS DPO | 1 | | Downgrade | No downgrade | Downgrade | Downgrade | No downgrade | Very low |
| rhEPO VS DPO | 2 | | Downgrade | No downgrade | Downgrade | Downgrade | No downgrade | Very low |
| DPO VS MPG-EPO | 1 | | Downgrade | No downgrade | Downgrade | Downgrade | No downgrade | Very low |
| Roxadustat VS Daprodustat | - | | No downgrade | No downgrade | No downgrade | Downgrade | No downgrade | Low |
| Roxadustat VS Vadadustat | - | | Downgrade | No downgrade | Downgrade | Downgrade | No downgrade | Very low |
| Roxadustat VS Molidustat | - | | Downgrade | No downgrade | Downgrade | Downgrade | No downgrade | Very low |
| Roxadustat VS MPG-EPO | - | | Downgrade | No downgrade | Downgrade | Downgrade | No downgrade | Very low |
| DaprodustatVS Vadadustat | - | | Downgrade | No downgrade | Downgrade | Downgrade | No downgrade | Very low |
| DaprodustatVS Molidustat | - | | No downgrade | No downgrade | Downgrade | Downgrade | No downgrade | Very low |
| DaprodustatVS MPG-EPO | - | | No downgrade | No downgrade | Downgrade | Downgrade | No downgrade | Very low |
| Vadadustat VS Molidustat | - | | Downgrade | No downgrade | Downgrade | Downgrade | No downgrade | Very low |
| Vadadustat VS rhEPO | - | | Downgrade | No downgrade | Downgrade | Downgrade | No downgrade | Very low |
| Vadadustat VS MPG-EPO | - | | Downgrade | No downgrade | Downgrade | Downgrade | No downgrade | Very low |
| Molidustat VS MPG-EPO | - | | Downgrade | No downgrade | Downgrade | Downgrade | No downgrade | Very low |
| rhEPO VS MPG-EPO | - | | Downgrade | No downgrade | Downgrade | Downgrade | No downgrade | Very low |
| **Content withdrawn in discontinuation** | | | | | | | | |
| Roxadustat VS rhEPO | 5 | | Downgrade | No downgrade | Downgrade | Downgrade | No downgrade | Very low |
| Roxadustat VS DPO | 1 | | Downgrade | No downgrade | Downgrade | Downgrade | No downgrade | Very low |
| DaprodustatVS rhEPO | 3 | | No downgrade | No downgrade | Downgrade | Downgrade | No downgrade | Low |
| DaprodustatVS DPO | 1 | | No downgrade | No downgrade | Downgrade | Downgrade | No downgrade | Low |
| Vadadustat VS DPO | 3 | | Downgrade | No downgrade | No downgrade | Downgrade | No downgrade | Low |
| Enarodustat VS DPO | 1 | | No downgrade | No downgrade | Downgrade | Downgrade | No downgrade | Low |
| Molidustat VS rhEPO | 1 | | Downgrade | No downgrade | Downgrade | Downgrade | No downgrade | Very low |
| Molidustat VS DPO | 1 | | Downgrade | No downgrade | Downgrade | Downgrade | No downgrade | Very low |
| Desidustat VS DPO | 1 | | Downgrade | No downgrade | Downgrade | Downgrade | No downgrade | Very low |
| rhEPO VS DPO | 2 | | Downgrade | No downgrade | Downgrade | Downgrade | No downgrade | Very low |
| Roxadustat VS Daprodustat | - | | No downgrade | No downgrade | Downgrade | Downgrade | No downgrade | Very low |
| Roxadustat VS Vadadustat | - | | Downgrade | No downgrade | Downgrade | Downgrade | No downgrade | Very low |
| Roxadustat VS Enarodustat | - | | No downgrade | No downgrade | Downgrade | Downgrade | No downgrade | Very low |
| Roxadustat VS Molidustat | - | | Downgrade | No downgrade | Downgrade | Downgrade | No downgrade | Very low |
| Roxadustat VS Desidustat | - | | Downgrade | No downgrade | Downgrade | Downgrade | No downgrade | Very low |
| DaprodustatVS Vadadustat | - | | Downgrade | No downgrade | Downgrade | Downgrade | No downgrade | Very low |
| DaprodustatVS Enarodustat | - | | No downgrade | No downgrade | Downgrade | Downgrade | No downgrade | Very low |
| DaprodustatVS Molidustat | - | | No downgrade | No downgrade | Downgrade | Downgrade | No downgrade | Very low |
| DaprodustatVS Desidustat | - | | No downgrade | No downgrade | Downgrade | Downgrade | No downgrade | Very low |
| Vadadustat VS Enarodustat | - | | No downgrade | No downgrade | Downgrade | Downgrade | No downgrade | Very low |
| Vadadustat VS Molidustat | - | | Downgrade | No downgrade | No downgrade | Downgrade | No downgrade | Very low |
| Vadadustat VS Desidustat | - | | Downgrade | No downgrade | Downgrade | Downgrade | No downgrade | Very low |
| Vadadustat VS rhEPO | - | | Downgrade | No downgrade | No downgrade | Downgrade | No downgrade | Very low |
| Enarodustat VS Molidustat | - | | No downgrade | No downgrade | Downgrade | Downgrade | No downgrade | Very low |
| Enarodustat VS Desidustat | - | | No downgrade | No downgrade | Downgrade | Downgrade | No downgrade | Very low |
| Enarodustat VS rhEPO | - | | No downgrade | No downgrade | Downgrade | Downgrade | No downgrade | Very low |
| Molidustat VS Desidustat | - | | Downgrade | No downgrade | Downgrade | Downgrade | No downgrade | Very low |
| Desidustat VS DPO | - | | Downgrade | No downgrade | Downgrade | Downgrade | No downgrade | Very low |
| **Kidney transplantation in discontinuation** | | | | | | | | |
| Roxadustat VS rhEPO | 3 | | Downgrade | No downgrade | Downgrade | No downgrade | No downgrade | Low |
| DaprodustatVS rhEPO | 1 | | No downgrade | No downgrade | Downgrade | No downgrade | No downgrade | Moderate |
| DaprodustatVS DPO | 1 | | No downgrade | No downgrade | Downgrade | No downgrade | No downgrade | Moderate |
| Vadadustat VS DPO | 2 | | Downgrade | No downgrade | Downgrade | No downgrade | No downgrade | Low |
| rhEPO VS DPO | 2 | | Downgrade | No downgrade | Downgrade | No downgrade | No downgrade | Low |
| rhEPO VS MPG-EPO | 1 | | Downgrade | No downgrade | Downgrade | No downgrade | No downgrade | Low |
| DPO VS MPG-EPO | 1 | | Downgrade | No downgrade | Downgrade | No downgrade | No downgrade | Low |
| Roxadustat VS Daprodustat | - | | No downgrade | No downgrade | Downgrade | No downgrade | No downgrade | Low |
| Roxadustat VS Vadadustat | - | | Downgrade | No downgrade | Downgrade | No downgrade | No downgrade | Very low |
| Roxadustat VS DPO | - | | Downgrade | No downgrade | Downgrade | No downgrade | No downgrade | Very low |
| Roxadustat VS MPG-EPO | - | | Downgrade | No downgrade | Downgrade | No downgrade | No downgrade | Very low |
| Daprodustat VS Vadadustat | - | | Downgrade | No downgrade | Downgrade | No downgrade | No downgrade | Very low |
| Daprodustat VS MPG-EPO | - | | No downgrade | No downgrade | Downgrade | No downgrade | No downgrade | Low |
| Vadadustat VS rhEPO | - | | Downgrade | No downgrade | Downgrade | No downgrade | No downgrade | Very low |
| Vadadustat VS MPG-EPO | - | | Downgrade | No downgrade | Downgrade | No downgrade | No downgrade | Very low |
| **AEs** | | | | | | | | |
| Roxadustat VS rhEPO | 5 | Downgrade | | No downgrade | No downgrade | No downgrade | No downgrade | Moderate |
| Roxadustat VS DPO | 1 | Downgrade | | No downgrade | No downgrade | No downgrade | No downgrade | Moderate |
| DaprodustatVS rhEPO | 3 | Downgrade | | No downgrade | No downgrade | Downgrade | No downgrade | Low |
| DaprodustatVS DPO | 2 | Downgrade | | No downgrade | No downgrade | No downgrade | No downgrade | Moderate |
| Vadadustat VS DPO | 2 | Downgrade | | No downgrade | Downgrade | No downgrade | No downgrade | Low |
| Enarodustat VS DPO | 1 | Downgrade | | No downgrade | No downgrade | No downgrade | No downgrade | Moderate |
| Molidustat VS rhEPO | 1 | Downgrade | | No downgrade | No downgrade | No downgrade | No downgrade | Moderate |
| Molidustat VS DPO | 1 | No downgrade | | No downgrade | No downgrade | No downgrade | No downgrade | High |
| Desidustat VS rhEPO | 1 | Downgrade | | No downgrade | No downgrade | No downgrade | No downgrade | Moderate |
| rhEPO VS DPO | 3 | Downgrade | | No downgrade | No downgrade | No downgrade | No downgrade | Moderate |
| rhEPO VS MPG-EPO | 2 | Downgrade | | No downgrade | No downgrade | No downgrade | No downgrade | Moderate |
| DPO VS MPG-EPO | 1 | Downgrade | | No downgrade | No downgrade | No downgrade | No downgrade | Moderate |
| Roxadustat VS Daprodustat | - | Downgrade | | No downgrade | No downgrade | Downgrade | No downgrade | Low |
| Roxadustat VS Vadadustat | - | Downgrade | | No downgrade | No downgrade | No downgrade | No downgrade | Low |
| Roxadustat VS Enarodustat | - | Downgrade | | No downgrade | No downgrade | No downgrade | No downgrade | Low |
| Roxadustat VS Molidustat | - | No downgrade | | No downgrade | No downgrade | No downgrade | No downgrade | Moderate |
| Roxadustat VS Desidustat | - | Downgrade | | No downgrade | No downgrade | No downgrade | No downgrade | Low |
| Roxadustat VS MPG-EPO | - | Downgrade | | No downgrade | No downgrade | No downgrade | No downgrade | Low |
| DaprodustatVS Vadadustat | - | Downgrade | | No downgrade | No downgrade | No downgrade | No downgrade | Low |
| DaprodustatVS Enarodustat | - | Downgrade | | No downgrade | No downgrade | No downgrade | No downgrade | Low |
| DaprodustatVS Molidustat | - | No downgrade | | No downgrade | No downgrade | No downgrade | No downgrade | Moderate |
| DaprodustatVS Desidustat | - | Downgrade | | No downgrade | No downgrade | No downgrade | No downgrade | Low |
| DaprodustatVS MPG-EPO | - | Downgrade | | No downgrade | No downgrade | No downgrade | No downgrade | Low |
| Vadadustat VS Enarodustat | - | No downgrade | | No downgrade | No downgrade | No downgrade | No downgrade | Moderate |
| Vadadustat VS Molidustat | - | Downgrade | | No downgrade | No downgrade | No downgrade | No downgrade | Low |
| Vadadustat VS Desidustat | - | Downgrade | | No downgrade | No downgrade | No downgrade | No downgrade | Low |
| Vadadustat VS rhEPO | - | Downgrade | | No downgrade | No downgrade | No downgrade | No downgrade | Low |
| Vadadustat VS MPG-EPO | - | No downgrade | | No downgrade | No downgrade | No downgrade | No downgrade | Moderate |
| Enarodustat VS Molidustat | - | No downgrade | | No downgrade | No downgrade | No downgrade | No downgrade | Moderate |
| Enarodustat VS Desidustat | - | No downgrade | | No downgrade | No downgrade | No downgrade | No downgrade | Moderate |
| Enarodustat VS rhEPO | - | No downgrade | | No downgrade | No downgrade | No downgrade | No downgrade | Moderate |
| Enarodustat VS MPG-EPO | - | Downgrade | | No downgrade | No downgrade | No downgrade | No downgrade | Low |
| Molidustat VS Desidustat | - | Downgrade | | No downgrade | No downgrade | No downgrade | No downgrade | Low |
| Molidustat VS MPG-EPO | - | Downgrade | | No downgrade | No downgrade | No downgrade | No downgrade | Low |
| Desidustat VS DPO | - | Downgrade | | No downgrade | No downgrade | No downgrade | No downgrade | Low |
| Desidustat VS MPG-EPO | - | Downgrade | | No downgrade | No downgrade | No downgrade | No downgrade | Low |
| **SAEs** | | | | | | | | |
| Roxadustat VS rhEPO | 5 | Downgrade | | No downgrade | No downgrade | Downgrade | No downgrade | Low |
| Roxadustat VS DPO | 1 | Downgrade | | No downgrade | No downgrade | Downgrade | No downgrade | Low |
| DaprodustatVS rhEPO | 3 | No downgrade | | No downgrade | No downgrade | Downgrade | No downgrade | Moderate |
| DaprodustatVS DPO | 2 | No downgrade | | No downgrade | No downgrade | Downgrade | No downgrade | Moderate |
| Vadadustat VS DPO | 2 | Downgrade | | No downgrade | No downgrade | No downgrade | No downgrade | Moderate |
| Enarodustat VS DPO | 1 | Downgrade | | No downgrade | No downgrade | No downgrade | No downgrade | Moderate |
| Molidustat VS rhEPO | 1 | Downgrade | | No downgrade | No downgrade | No downgrade | No downgrade | Moderate |
| Molidustat VS DPO | 1 | No downgrade | | No downgrade | No downgrade | No downgrade | No downgrade | High |
| Desidustat VS rhEPO | 1 | Downgrade | | No downgrade | No downgrade | No downgrade | No downgrade | Moderate |
| rhEPO VS DPO | 2 | No downgrade | | No downgrade | No downgrade | No downgrade | No downgrade | High |
| rhEPO VS MPG-EPO | 2 | Downgrade | | No downgrade | No downgrade | No downgrade | No downgrade | Moderate |
| DPO VS MPG-EPO | 1 | Downgrade | | No downgrade | No downgrade | No downgrade | No downgrade | Moderate |
| Roxadustat VS Daprodustat | - | No downgrade | | No downgrade | No downgrade | No downgrade | No downgrade | Moderate |
| Roxadustat VS Vadadustat | - | Downgrade | | No downgrade | No downgrade | No downgrade | No downgrade | Low |
| Roxadustat VS Enarodustat | - | No downgrade | | No downgrade | No downgrade | No downgrade | No downgrade | Moderate |
| Roxadustat VS Molidustat | - | Downgrade | | No downgrade | No downgrade | No downgrade | No downgrade | Low |
| Roxadustat VS Desidustat | - | Downgrade | | No downgrade | No downgrade | No downgrade | No downgrade | Low |
| Roxadustat VS MPG-EPO | - | Downgrade | | No downgrade | No downgrade | No downgrade | No downgrade | Low |
| DaprodustatVS Vadadustat | - | Downgrade | | No downgrade | No downgrade | No downgrade | No downgrade | Low |
| DaprodustatVS Enarodustat | - | No downgrade | | No downgrade | No downgrade | No downgrade | No downgrade | Moderate |
| DaprodustatVS Molidustat | - | No downgrade | | No downgrade | No downgrade | No downgrade | No downgrade | Moderate |
| DaprodustatVS Desidustat | - | Downgrade | | No downgrade | No downgrade | No downgrade | No downgrade | Low |
| DaprodustatVS MPG-EPO | - | No downgrade | | No downgrade | No downgrade | No downgrade | No downgrade | Moderate |
| Vadadustat VS Enarodustat | - | Downgrade | | No downgrade | No downgrade | No downgrade | No downgrade | Low |
| Vadadustat VS Molidustat | - | Downgrade | | No downgrade | No downgrade | No downgrade | No downgrade | Low |
| Vadadustat VS Desidustat | - | Downgrade | | No downgrade | No downgrade | No downgrade | No downgrade | Low |
| Vadadustat VS rhEPO | - | No downgrade | | No downgrade | No downgrade | No downgrade | No downgrade | Moderate |
| Vadadustat VS MPG-EPO | - | No downgrade | | No downgrade | No downgrade | No downgrade | No downgrade | Moderate |
| Enarodustat VS Molidustat | - | No downgrade | | No downgrade | No downgrade | No downgrade | No downgrade | Moderate |
| Enarodustat VS Desidustat | - | No downgrade | | No downgrade | No downgrade | No downgrade | No downgrade | Moderate |
| Enarodustat VS rhEPO | - | Downgrade | | No downgrade | No downgrade | No downgrade | No downgrade | Low |
| Enarodustat VS MPG-EPO | - | No downgrade | | No downgrade | No downgrade | No downgrade | No downgrade | Moderate |
| Molidustat VS Desidustat | - | Downgrade | | No downgrade | No downgrade | No downgrade | No downgrade | Low |
| Molidustat VS MPG-EPO | - | Downgrade | | No downgrade | No downgrade | No downgrade | No downgrade | Low |
| Desidustat VS DPO | - | Downgrade | | No downgrade | No downgrade | No downgrade | No downgrade | Low |
| Desidustat VS MPG-EPO | - | Downgrade | | No downgrade | No downgrade | No downgrade | No downgrade | Low |
| **MACE** | | | | | | | | |
| Roxadustat VS rhEPO | 4 | | Downgrade | No downgrade | Downgrade | No downgrade | No downgrade | Low |
| Roxadustat VS DPO | 1 | | No downgrade | No downgrade | Downgrade | No downgrade | No downgrade | Moderate |
| DaprodustatVS rhEPO | 3 | | No downgrade | No downgrade | Downgrade | No downgrade | No downgrade | Moderate |
| DaprodustatVS DPO | 1 | | Downgrade | No downgrade | Downgrade | No downgrade | No downgrade | Low |
| Vadadustat VS DPO | 3 | | Downgrade | No downgrade | Downgrade | No downgrade | No downgrade | Low |
| Molidustat VS DPO | 1 | | No downgrade | No downgrade | Downgrade | No downgrade | No downgrade | Moderate |
| Roxadustat VS Daprodustat | - | | No downgrade | No downgrade | Downgrade | No downgrade | No downgrade | Low |
| Roxadustat VS Vadadustat | - | | Downgrade | No downgrade | Downgrade | No downgrade | No downgrade | Very low |
| Roxadustat VS Molidustat | - | | No downgrade | No downgrade | Downgrade | No downgrade | No downgrade | Low |
| DaprodustatVS Vadustate | - | | Downgrade | No downgrade | Downgrade | No downgrade | No downgrade | Very low |
| DaprodustatVS Molidustat | - | | No downgrade | No downgrade | Downgrade | No downgrade | No downgrade | Low |
| Vadadustat VS Molidustat | - | | No downgrade | No downgrade | Downgrade | No downgrade | No downgrade | Low |
| Vadadustat VS rhEPO | - | | Downgrade | No downgrade | Downgrade | No downgrade | No downgrade | Very low |
| Molidustat VS DPO | - | | No downgrade | No downgrade | Downgrade | No downgrade | No downgrade | Low |
| rhEPO VS DPO | - | | No downgrade | No downgrade | Downgrade | No downgrade | No downgrade | Low |
| **All-cause mortality** | | | | | | | | |
| Roxadustat VS rhEPO | 5 | | Downgrade | No downgrade | Downgrade | Downgrade | No downgrade | Very low |
| Roxadustat VS DPO | 1 | | Downgrade | No downgrade | Downgrade | Downgrade | No downgrade | Very low |
| DaprodustatVS rhEPO | 3 | | No downgrade | No downgrade | Downgrade | No downgrade | No downgrade | Moderate |
| DaprodustatVS DPO | 2 | | No downgrade | No downgrade | Downgrade | No downgrade | No downgrade | Moderate |
| Vadadustat VS DPO | 3 | | Downgrade | No downgrade | Downgrade | No downgrade | No downgrade | Low |
| Enarodustat VS DPO | 1 | | No downgrade | No downgrade | Downgrade | No downgrade | No downgrade | Moderate |
| Molidustat VS rhEPO | 1 | | No downgrade | No downgrade | Downgrade | No downgrade | No downgrade | Moderate |
| Molidustat VS DPO | 1 | | No downgrade | No downgrade | Downgrade | No downgrade | No downgrade | Moderate |
| Desidustat VS rhEPO | 1 | | Downgrade | No downgrade | Downgrade | No downgrade | No downgrade | Low |
| rhEPO VS DPO | 2 | | Downgrade | No downgrade | Downgrade | Downgrade | No downgrade | Very low |
| rhEPO VS MPG-EPO | 2 | | Downgrade | No downgrade | Downgrade | No downgrade | No downgrade | Low |
| DPO VS MPG-EPO | 1 | | Downgrade | No downgrade | Downgrade | No downgrade | No downgrade | Low |
| Roxadustat VS Daprodustat | - | | No downgrade | No downgrade | Downgrade | No downgrade | No downgrade | Low |
| Roxadustat VS Vadadustat | - | | Downgrade | No downgrade | Downgrade | No downgrade | No downgrade | Very low |
| Roxadustat VS Enarodustat | - | | No downgrade | No downgrade | Downgrade | No downgrade | No downgrade | Low |
| Roxadustat VS Molidustat | - | | Downgrade | No downgrade | Downgrade | No downgrade | No downgrade | Very low |
| Roxadustat VS Desidustat | - | | Downgrade | No downgrade | Downgrade | No downgrade | No downgrade | Very low |
| Roxadustat VS MPG-EPO | - | | Downgrade | No downgrade | Downgrade | No downgrade | No downgrade | Very low |
| DaprodustatVS Vadadustat | - | | Downgrade | No downgrade | Downgrade | No downgrade | No downgrade | Very low |
| DaprodustatVS Enarodustat | - | | No downgrade | No downgrade | Downgrade | No downgrade | No downgrade | Low |
| DaprodustatVS Molidustat | - | | No downgrade | No downgrade | Downgrade | No downgrade | No downgrade | Low |
| DaprodustatVS Desidustat | - | | No downgrade | No downgrade | Downgrade | No downgrade | No downgrade | Low |
| DaprodustatVS MPG-EPO | - | | No downgrade | No downgrade | Downgrade | No downgrade | No downgrade | Low |
| Vadadustat VS Enarodustat | - | | No downgrade | No downgrade | Downgrade | No downgrade | No downgrade | Low |
| Vadadustat VS Molidustat | - | | Downgrade | No downgrade | Downgrade | No downgrade | No downgrade | Very low |
| Vadadustat VS Desidustat | - | | Downgrade | No downgrade | Downgrade | No downgrade | No downgrade | Very low |
| Vadadustat VS rhEPO | - | | Downgrade | No downgrade | Downgrade | No downgrade | No downgrade | Very low |
| Vadadustat VS MPG-EPO | - | | Downgrade | No downgrade | Downgrade | No downgrade | No downgrade | Very low |
| Enarodustat VS Molidustat | - | | No downgrade | No downgrade | Downgrade | No downgrade | No downgrade | Low |
| Enarodustat VS Desidustat | - | | No downgrade | No downgrade | Downgrade | No downgrade | No downgrade | Low |
| Enarodustat VS rhEPO | - | | No downgrade | No downgrade | Downgrade | No downgrade | No downgrade | Low |
| Enarodustat VS MPG-EPO | - | | No downgrade | No downgrade | Downgrade | No downgrade | No downgrade | Low |
| Molidustat VS Desidustat | - | | Downgrade | No downgrade | Downgrade | No downgrade | No downgrade | Very low |
| Molidustat VS MPG-EPO | - | | Downgrade | No downgrade | Downgrade | No downgrade | No downgrade | Very low |
| Desidustat VS DPO | - | | Downgrade | No downgrade | Downgrade | No downgrade | No downgrade | Very low |
| Desidustat VS MPG-EPO | - | | Downgrade | No downgrade | Downgrade | No downgrade | No downgrade | Very low |
| **AEs** | | | | | | | | |
| Roxadustat VS rhEPO | 5 | | Downgrade | No downgrade | No downgrade | Downgrade | No downgrade | Low |
| Roxadustat VS DPO | 1 | | Downgrade | No downgrade | Downgrade | Downgrade | No downgrade | Very low |
| DaprodustatVS rhEPO | 2 | | No downgrade | No downgrade | No downgrade | Downgrade | No downgrade | Moderate |
| DaprodustatVS DPO | 2 | | Downgrade | No downgrade | Downgrade | Downgrade | No downgrade | Very low |
| Vadadustat VS DPO | 2 | | Downgrade | No downgrade | No downgrade | Downgrade | No downgrade | Low |
| Enarodustat VS DPO | 1 | | No downgrade | No downgrade | Downgrade | Downgrade | No downgrade | Low |
| Desidustat VS DPO | 1 | | Downgrade | No downgrade | Downgrade | Downgrade | No downgrade | Very low |
| Roxadustat VS Daprodustat | - | | Downgrade | No downgrade | Downgrade | Downgrade | No downgrade | Very low |
| Roxadustat VS Vadadustat | - | | Downgrade | No downgrade | Downgrade | Downgrade | No downgrade | Very low |
| Roxadustat VS Enarodustat | - | | No downgrade | No downgrade | Downgrade | Downgrade | No downgrade | Very low |
| Roxadustat VS Desidustat | - | | Downgrade | No downgrade | Downgrade | Downgrade | No downgrade | Very low |
| DaprodustatVS Vadadustat | - | | Downgrade | No downgrade | Downgrade | Downgrade | No downgrade | Very low |
| DaprodustatVS Enarodustat | - | | No downgrade | No downgrade | Downgrade | Downgrade | No downgrade | Very low |
| DaprodustatVS Desidustat | - | | No downgrade | No downgrade | Downgrade | Downgrade | No downgrade | Very low |
| Vadadustat VS Enarodustat | - | | No downgrade | No downgrade | Downgrade | Downgrade | No downgrade | Very low |
| Vadadustat VS Desidustat | - | | Downgrade | No downgrade | Downgrade | Downgrade | No downgrade | Very low |
| Vadadustat VS rhEPO | - | | Downgrade | No downgrade | No downgrade | Downgrade | No downgrade | Very low |
| Enarodustat VS rhEPO | - | | No downgrade | No downgrade | No downgrade | Downgrade | No downgrade | Low |
| Enarodustat VS Desidustat | - | | No downgrade | No downgrade | Downgrade | Downgrade | No downgrade | Very low |
| Desidustat VS DPO | - | | Downgrade | No downgrade | Downgrade | Downgrade | No downgrade | Very low |
| rhEPO VS DPO | - | | Downgrade | No downgrade | No downgrade | Downgrade | No downgrade | Very low |
| **SAEs** | | | | | | | | |
| Roxadustat VS rhEPO | 5 | | Downgrade | No downgrade | No downgrade | Downgrade | No downgrade | Low |
| Roxadustat VS DPO | 1 | | Downgrade | No downgrade | Downgrade | Downgrade | No downgrade | Very low |
| DaprodustatVS rhEPO | 2 | | Downgrade | No downgrade | Downgrade | Downgrade | No downgrade | Very low |
| DaprodustatVS DPO | 1 | | Downgrade | No downgrade | Downgrade | Downgrade | No downgrade | Very low |
| Vadadustat VS DPO | 2 | | Downgrade | No downgrade | Downgrade | Downgrade | No downgrade | Very low |
| Enarodustat VS DPO | 1 | | No downgrade | No downgrade | Downgrade | Downgrade | No downgrade | Low |
| rhEPO VS DPO | 1 | | Downgrade | No downgrade | Downgrade | Downgrade | No downgrade | Very low |
| Roxadustat VS Daprodustat | - | | Downgrade | No downgrade | Downgrade | Downgrade | No downgrade | Very low |
| Roxadustat VS Vadadustat | - | | Downgrade | No downgrade | Downgrade | Downgrade | No downgrade | Very low |
| Roxadustat VS Enarodustat | - | | No downgrade | No downgrade | Downgrade | Downgrade | No downgrade | Very low |
| DaprodustatVS Vadadustat | - | | Downgrade | No downgrade | Downgrade | Downgrade | No downgrade | Very low |
| DaprodustatVS Enarodustat | - | | No downgrade | No downgrade | Downgrade | Downgrade | No downgrade | Very low |
| Vadadustat VS Enarodustat | - | | No downgrade | No downgrade | Downgrade | Downgrade | No downgrade | Very low |
| Vadadustat VS rhEPO | - | | Downgrade | No downgrade | Downgrade | Downgrade | No downgrade | Very low |
| Enarodustat VS rhEPO | - | | No downgrade | No downgrade | Downgrade | Downgrade | No downgrade | Very low |

**Table S7: Network estimates of treatment comparisons for RBC transfusion**

**
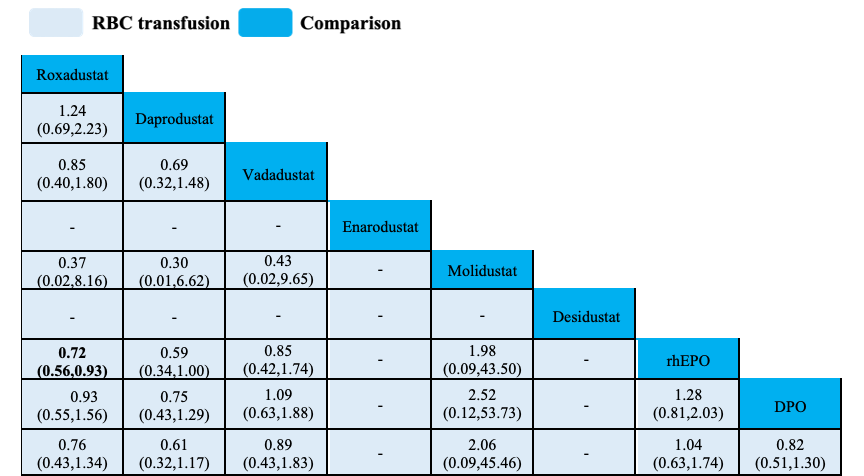
**

**Table S8: Network estimates of treatment comparisons for AEs and death in discontinuation**

**
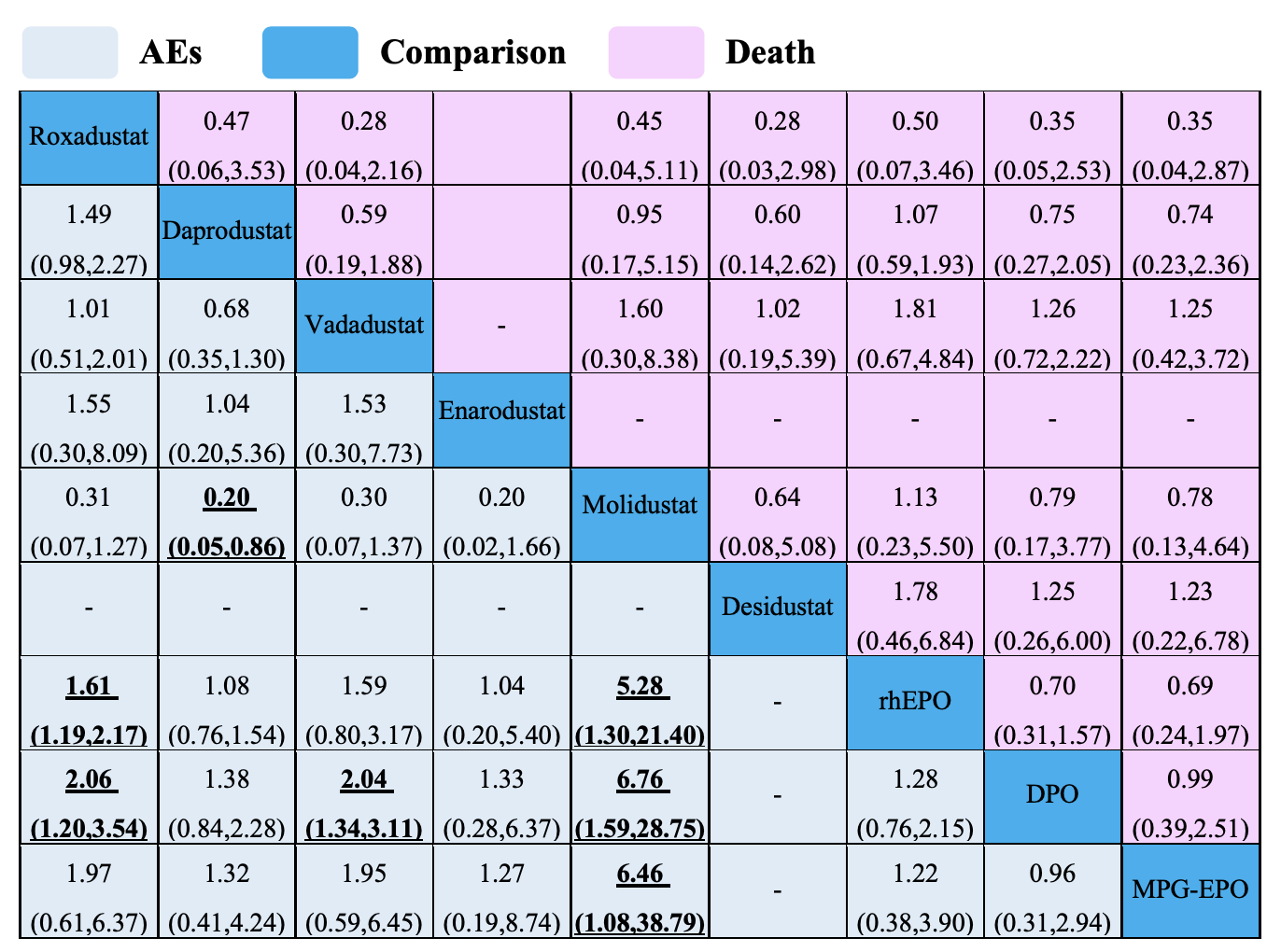
**

**Table S9: Refusal of treatment and kidney transplantation in discontinuation**

**
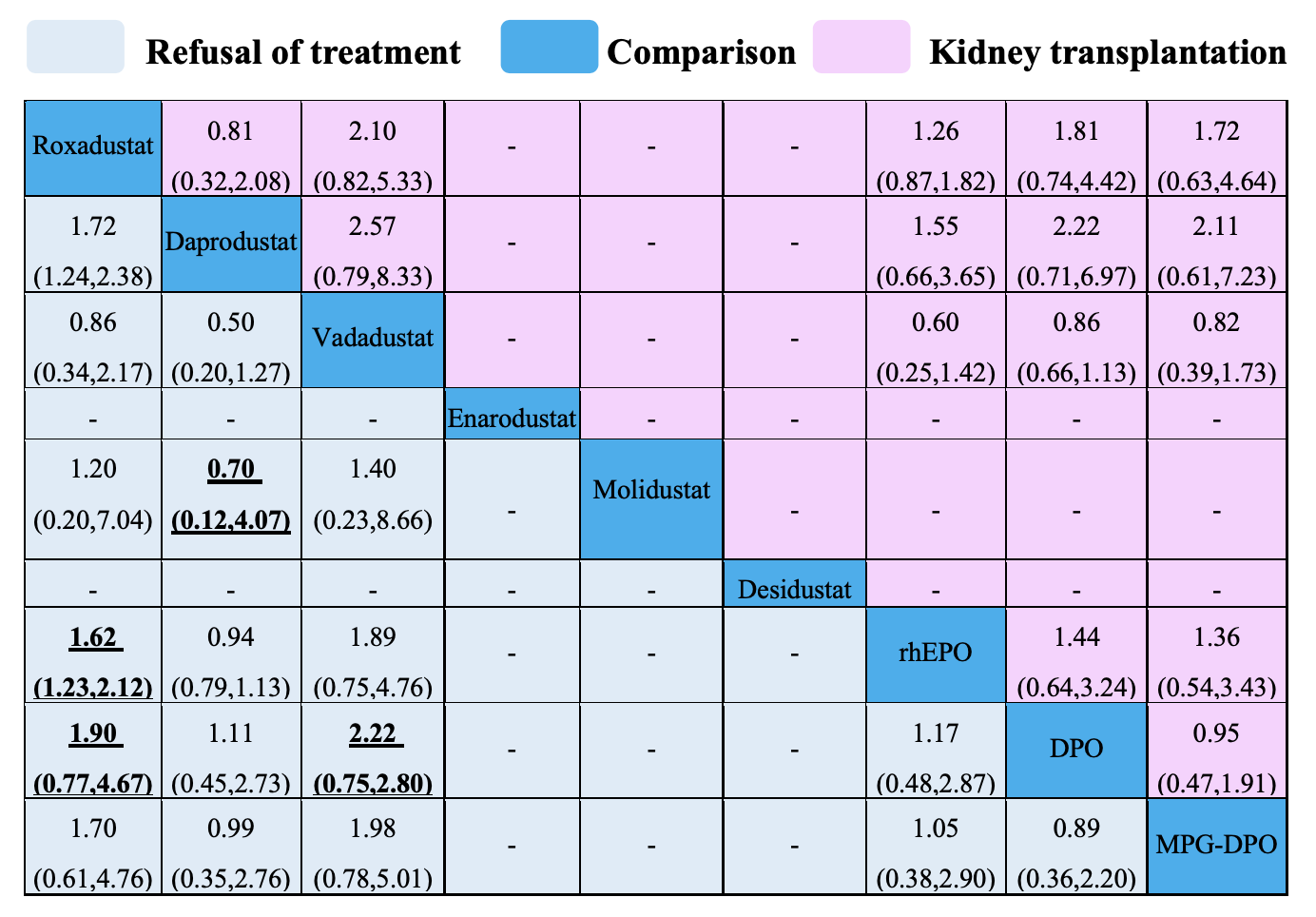
**

**Table S10: Network estimates of treatment comparisons for Content withdrawn in discontinuation and MACE**

**
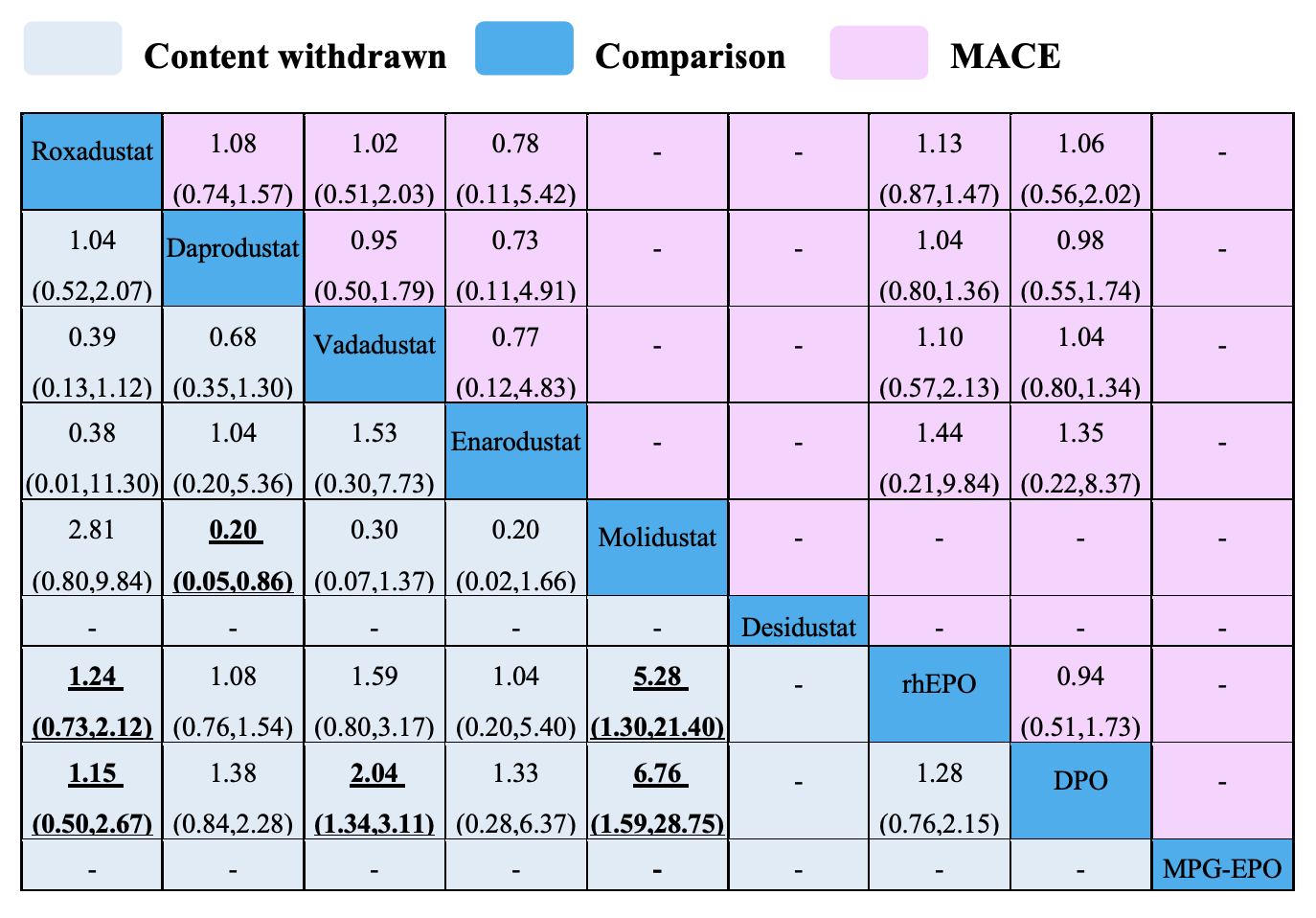
**

**Table S11: Network estimates of treatment comparisons for Hepcidin and Ferritin**


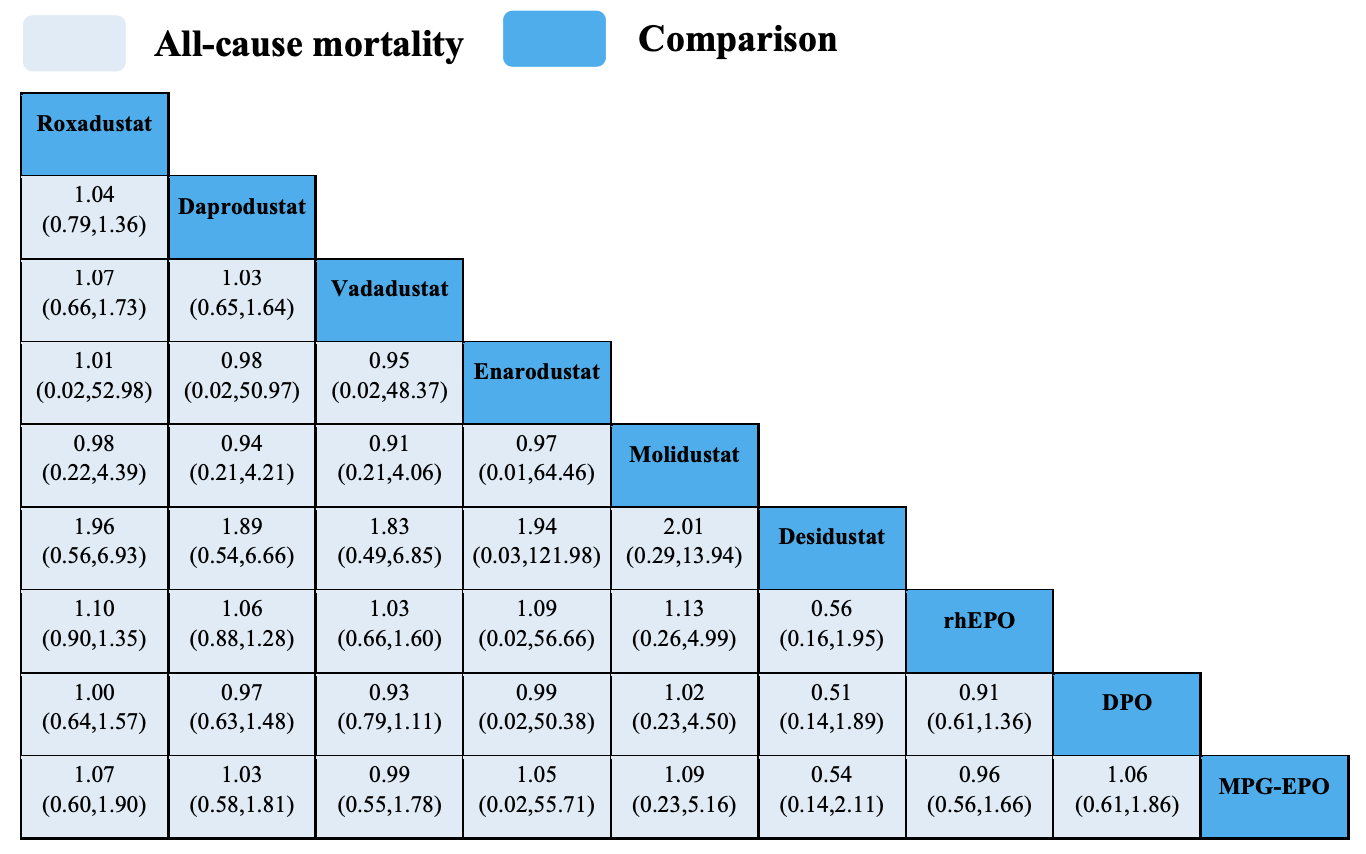

The summary estimates are odds ratios and 95% confidence intervals. For AEs , refusal of treatment, content withdrawn, hepcidin(ng/L)，TSAT（%）,All-cause mortality(% ) the odds ratio is for the row treatment compared to the column treatment. For death, kidney transplantation ,MACE, ferritin(ng/mL), TIBC（umol/L）the odds ratio is for the column treatment versus the row treatment.
